# Single-Cell Sequencing Identifies the Heterogeneity of CD8+ T Cells and Novel Biomarker Genes in Hepatocellular Carcinoma

**DOI:** 10.1155/2022/8256314

**Published:** 2022-04-12

**Authors:** Hailei Wang, Yang Fu, Bin-Bin Da, Geng Xiong

**Affiliations:** ^1^Kunming First People's Hospital, Kunming 650031, China; ^2^CT Room, Kunming First People's Hospital, Kunming 650000, China; ^3^Department of Minimally Invasive Interventional Medicine, Yunnan Cancer Hospital, Kunming 650118, China; ^4^Third Department of General Surgery, Changde First People's Hospital, Changde 415003, China

## Abstract

CD8+ T cells are required for the establishment of antitumor immunity, and their substantial infiltration is associated with a good prognosis. However, CD8+ T cell subsets in the tumor microenvironment may play distinct roles in tumor progression, prognosis, and immunotherapy. In this study, we used the scRNA-seq data of hepatocellular carcinoma (HCC) to reveal the heterogeneity of different CD8+ T cell subsets. The scRNA-seq data set GSE149614 was obtained from the GEO database, and the transcriptome and sample phenotypic data of TCGA-LIHC were obtained from the TCGA database. CD8+ T cell subtypes and metabolic gene sets were obtained from published reports. The data processing and analysis of CD8+ T cell groups was performed by *R* language. The PPI network was constructed to obtain the hub genes, and the KM survival curve of the hub genes was further plotted to determine the hub genes with differences in survival. CD8+ T cells in HCC were divided into 7 subsets, and the cytotoxic CD8 T cells 4 subset showed considerable differences between the TP53-mutant and nonmutant groups, as well as between different degrees of cirrhosis, HCC grades, stages, ages, and body weights. Cytotoxic CD8 T cells 4 differential genes were analyzed by TCGA-LIHC data and single-cell sequencing data set. 10 hub genes were found: FGA, ApoA1, ApoH, AHSG, FGB, HP, TTR, TF, HPX, and APOC3. Different subsets of CD8+ T cells were found to contribute to heterogeneous prognosis and pathway activity in HCC. Alterations in the cytotoxic and immune checkpoint gene expression during CD8+ T cell differentiation were also identified. We found that cytotoxic CD8 T cells 4 is closely associated with survival and prognosis of HCC and identified four differential genes that can be used as biological markers for survival, prognosis, and clinically relevant characteristics of HCC. Results of this study could help finding targets for immunotherapy of HCC and aid in the accelerated development of immunotherapy for HCC.

## 1. Introduction

Hepatocellular carcinoma (HCC) is a type of liver cancer that is extremely common [1]. Traditional treatment methods for HCC mainly include surgical treatment (liver resection, liver transplantation), radiofrequency, microwave ablation, embolization (transcatheter hepatic arterial chemoembolization, TACE), and sorafenib [2–6]. In the early stage of hepatocellular carcinoma, the symptoms are hidden without characteristics; hence, most of the patients have developed to the middle and late stage when diagnosed, most of them cannot accept radical treatment, and the prognosis is poor [7]. In recent years, immunotherapy has produced good therapeutic effects in the therapy of various malignant tumors, including melanoma and hematological malignancies, while the therapeutic effects in solid malignant tumors are not satisfactory, especially in HCC [8, 9]. On the one hand, this is related to the complex microenvironment in solid tumors, including low oxygen, high pH, nutritional deficiency, and immunosuppressive cells and factors [10–13]; and on the other hand, the disorder of the functional state of the relevant immune cells in the tumor is also an important factor leading to ineffective or inefficient immunotherapy [14].

Immune imbalance in the microenvironment of the tumor is one of the important characteristics of the tumor. The adaptive immune response, which is mediated by immune cells, is important in the occurrence and development of tumors [15]. CD8+ T cells are the main antitumor effector cells [16]. The weakened antitumor immunity characterized by CD8+ T cell function disorder plays an essential role in the occurrence and development of hepatocellular carcinoma [17, 18]. CD8+ T cells from the circulation migrate and infiltrate into tumor tissues and are stimulated by contact with tumor antigens to become effective CD8+ T cells with the killing effect of tumor microcytes [19, 20].

In the process of antitumor immune response in normal organisms, antigen-presenting cells present tumor-specific antigen (TSA) as major histocompatibility complex (MHC) and bind to T cell surface TCR (T cell receptor) and then, under the action of various costimulatory signal molecules, activate T cells; activated T cells, mainly cytotoxic CD8+ T cells, bind to tumor cells via recognition of TSA on their surface and kill tumor cells after the costimulatory signal is activated [21, 22]. The activation of costimulatory signal plays an essential role in the killing of tumor by T cells [23]. In tumor microenvironment (TME) T cells, the expressions of costimulatory signal molecules including CD137, CD28, and OX40 are often significantly decreased [24–26], while the expressions of costimulatory signal molecules such as cytotoxic T-lymphocyte-associated protein 4 (CTLA4), programmed cell death protein 1 (PD-1), and T-cell immunoglobulin mucin-3 (TIM-3) are significantly increased [27–29]. These costimulating/inhibiting molecules are known as immune checkpoints, and the treatment of these signaling molecules is also known as immune checkpoint therapy.

Immunocheckpoint therapy has achieved a series of successes in the treatment of solid tumors, breaking through the original view that tumor immunotherapy may only be effective for immunogenic melanoma and kidney cancer [30]. Recent clinical studies have shown that monotherapy has achieved good efficacy in solid tumors such as non-small-cell lung cancer (NSCLC), colorectal cancer, and gastric cancer, and the safety of most immunotherapies has also been recognized [31–33]. The Food and Drug Administration (FDA) and the European Union have approved several immune checkpoint inhibitors and monoclonal antibodies for clinical tumor treatment, such as CTLA4 monoclonal antibody ipilimumab, PD1 monoclonal antibody nivolumab, etc. [34].

Like NSCLC and colorectal cancer, HCC cells also express a large number of co-inhibitory components such as PD-L1 on the surface, but unfortunately, the efficacy of immune checkpoint inhibitors has not been adequately evaluated in early clinical trials [35]. The current scRNA-seq technique is widely used in the study of cell heterogeneity. However, due to the need for preamplification of cDNA before library construction, poor amplification may lead to the loss of some information. To more comprehensively identify the differences among hepatocellular carcinoma cells, Xiao et al. [36] for the first time implemented the Holo-Seq technique to obtain the transcriptome information of mRNAs and small RNAs from a single cell at the same time and combined the information to determine intercellular heterogeneity. In this study, Holo-Seq technology was applied to analyze single cells of human liver cancer, and it was found that mitochondrial activity was downregulated in the early stage of liver cancer [36]. Moreover, tumor suppressor miRNA and tumor-promoting miRNA were upregulated earlier than the activation of classical tumor-promoting signaling pathway [36]. These findings provide important information and references for diagnosing liver cancer. Additionally, they drew a cluster map based on double transcriptional profiles of single-cell mRNAs and miRNAs in HCC [36]. Compared with single scRNA-seq clustering analysis, this approach enables a more complete understanding of the tumor cell heterogeneity in HCC and discover new cell subpopulations.

In this study, we used the scRNA-seq data of hepatocellular carcinoma to reveal the heterogeneity of different CD8+ T cell subsets. The cellular components of the CD8+ T cell subpopulation in liver cancer patients under different states of liver cirrhosis, grade, stage, age, and body weight were determined, and the activity analysis of metabolic pathway and hallmark pathway was carried out based on the pipeline analysis of pathway activity based on single-cell sequencing data. Cell differentiation trajectory and cell-cell interaction network analysis was performed, and the hub genes' expression in different cancers was determined for prognosis/treatment marker identification. Different subsets of CD8+ T cells were found to contribute to heterogeneous prognosis and pathway activity in HCC. Alterations in an immune checkpoint and cytotoxic gene expression during CD8+ T cell differentiation were also identified.

## 2. Materials and Methods

### 2.1. scRNA-seq and RNA-seq Data

HCC scRNA-seq was downloaded from the Gene Expression Omnibus (GEO) database (GSE149614), and a total of 34,414 cells from 10 HCC tissue samples were screened. From the TCGA Xena database (https://xenabrowser.net/datapages/), the transcriptome and sample phenotypic data of TCGA-LIHC were downloaded; a total of 424 samples were obtained, of which 374 were tumor tissues (6 of the samples had no survival information and were excluded from subsequent analysis) and 50 were normal tissues. The expressed value from log2 (count +1) were converted to count for subsequent analysis. CD8+ T cell subtypes were obtained from the study by Deng et al. [37]. A total of 85 metabolic gene sets were obtained from Xiao et al. [38], and a total of 50 hallmark gene sets were downloaded from h.all.v7.3.symbols.gmt from MSigDB.

### 2.2. Identification of CD8+ T Cell Subsets

The R package “Seurat” was used to map the expression profile of CD8A and CD8B (CD8+ T cell markers) and determine the CD8+ T cell population. CD8+ T cells were selected, and UMAP cluster analysis was performed again, and each cell subpopulation was annotated according to the expression distribution of CD8+ T cell subtypes. The proportion of each subtype of CD8+ T cells was counted, and a bar chart was drawn using the R package “ggplot2” for display. Hypervariable genes of each subtype were examined with the help of the Seurat package. To find all markers functions, the min.pct and logfc.threshold parameters set at 0.25 were used. The top 10 highly variable genes of each subtype were selected, and the R package “pheatmap” was used to draw the heatmap of gene expression. Marker genes of previously reported studies were employed to annotate subpopulations of CD8+ T cells including naive/memory CD8+ T cells (CCR7, IL7R, TCF7, SELL, SATB1, GPR183, LTB, LEF1, and S100A10), exhausted CD8+ T cells (CXCL13, HSPB1, IRF4, LAYN, GIMAP6, HSPH1, CXCR6, CTLA4, PDCD1, LAG3, HAVCR2, and TIGIT), and cytotoxic CD8+ T cells (PRF1, GZMA, GZMK, and NKG7).

### 2.3. The Cellular Components of the CD8+ T Cell Subpopulation

The Seurat package was used to obtain the hypervariable genes of each subtype of CD8+ T cells, and they were screened according to the Bonferroni correction *P* value <0.05. For screening of gene expression (count), CIBERSORTx (https://cibersortx.stanford.edu/) tools were used following the default parameters of CD8+ T cell subtype of liver cancer signature matrix file. The transcriptome data of TCGA-LIHC were collated, and the count was converted into CPM as the input file of CIBERSORTx to estimate the content of CD8+ T cell subtypes in liver cancer. The surv_cutpoint function was used to analyze the cell content of each subtype, and the cell content of TCGA-LIHC samples was divided into groups of high and low levels, and the survival curve of KM was plotted using Survival packet and survminer packet. The phenotypic data of TCGA-LIHC were sorted. Boxplots were drawn to show the difference in cell content between TP53 mutation and gender, and line plots were drawn to show the content of CD8+ T cell subtypes in liver cancer patients under different states of liver cirrhosis, grade, stage, age, and body weight.

### 2.4. Pathway Activity Analysis

For scRNA-seq data, the FindMarkers function of the Seurat package was used to obtain the hypervariable genes of each subtype of CD8+ T cells and screen them according to the Bonferroni correction *P* value <0.05. The ClusterProfiler package was used for Gene Ontology (GO) and Kyoto Encyclopedia of Genes and Genomes (KEGG) enrichment analysis, and then using ggplot2, a bubble chart was drawn to display the results. According to the research report of Xiao et al. [38], the activity analysis of metabolic pathway and hallmark pathway was carried out based on the pipeline analysis of pathway activity based on single-cell sequencing data.

### 2.5. Cell Differentiation Trajectory and Cell-Cell Interaction Network Analysis

Using the package's default parameters, the Slingshot package was utilized to assess the cell differentiation trajectory and the distribution of marker gene expression. The single-cell sequencing data of CD8+ T cells were collated, and the interaction network analysis of CD8+ T cell subtypes was conducted using CellPhoneDB software developed based on Python. The R package SCENIC was used to construct the gene regulation network of CD8+ T cell subtypes.

### 2.6. Prognosis/Treatment Marker Identification

CD8+ T cell subtypes with significant differences in survival analysis were selected and grouped according to the cell content of the cell subtypes. Differential analysis was performed on the TCGA-LIHC expression data (R package DESeq2), following |log2(FoldChange)|>1 and corrected the *P* value <0.05 for differential genes screening. The protein-protein interaction (PPI) network was constructed using the String database (https://www.string-db.org/), and Cytoscape (v3.7.2) and cytoHubba plug-in were used to screen the hub genes. The hub genes were divided into two groups based on their expression levels: high and low according to the median number of hub genes. Kaplan–Meier (KM) survival curves were plotted using the survival package R and Survminer. Hub genes with survival differences were selected and single-gene gene set enrichment analysis (GSEA) was performed using the clusterProfiler package. According to different clinical features, ggstatsplot was used to plot boxplots to show hub gene expression levels with significant differences in survival. The TCGA pan-cancer data was downloaded from the TCGA Xena database, and hub gene expression in various tumors was shown in a bar graph.

### 2.7. Statistical Analysis

Genes differently expressed in different subsets, KEGG, GO, and GSEA analyses were analyzed statistically using the corresponding software packages or default methods in the software. Random arrangement tests exhibited statistical pathway activity. Student's test was used to assess the frequency of different cell types in normal and tumor samples; statistical analysis was also used to examine the levels of expression in tumors and normal tissue samples. The significance of the KM curves was tested using the log-rank test. The Kruskal–Wallis test was used to determine dynamic changes in cell proportion and levels of gene expression at various stages of pathology.

## 3. Results

### 3.1. Data Download and Preprocessing

For liver cancer, the scRNA-seq data set GSE149614 was downloaded from the GEO database (https://www.ncbi.nlm.nih.gov/geo/) and 10 liver cancer tissue samples were screened with a total of 34,414 cells. From the TCGA Xena database (https://xenabrowser.net/datapages/), the transcriptome and sample phenotypic data of the TCGA-LIHC were downloaded; a total of 424 samples were obtained, of which 374 were tumor tissues (6 samples had no survival information and were excluded from subsequent analysis) and 50 were normal tissues.

**Figure 1:**
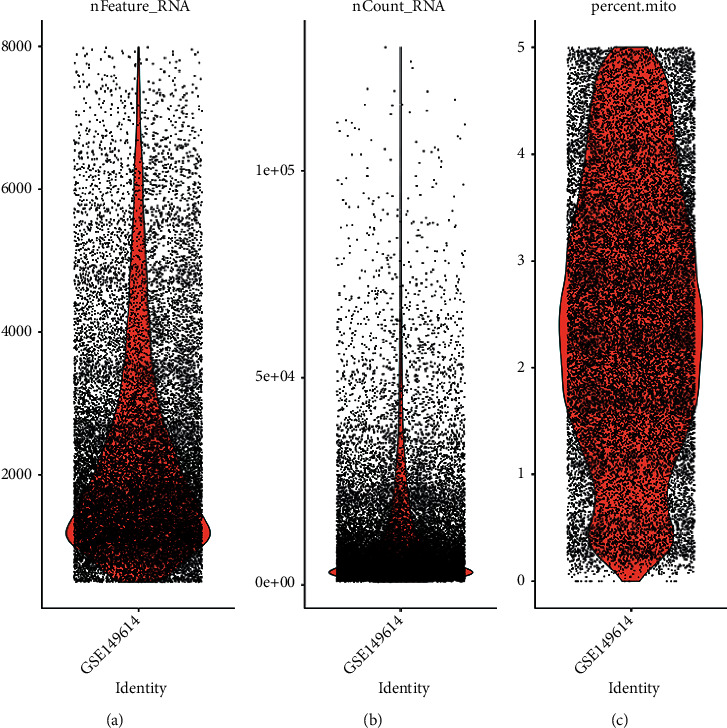
Quality control chart of GSE149614 single-cell data set. (a) The number of genes in a cell. (b) Count distribution of genes. (c) Figure: mitochondria percentage.

**Figure 2:**
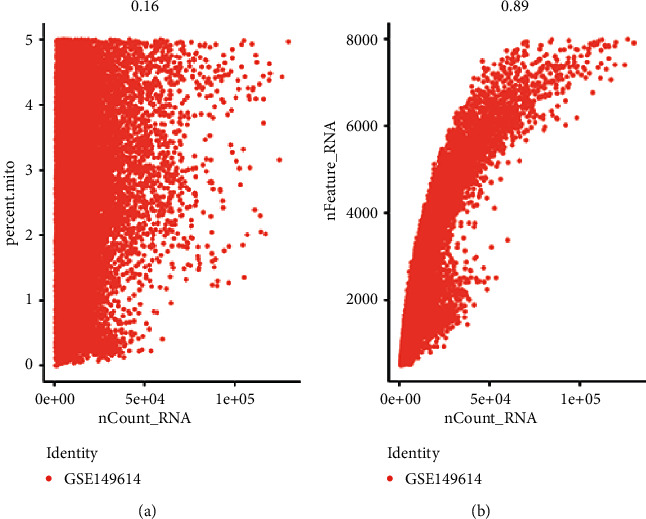
GSE149614 single-cell data quality control chart. (a) Correlation between mitochondrial percentage and gene count. (b) Correlation between the number of genes and count.

**Figure 3:**
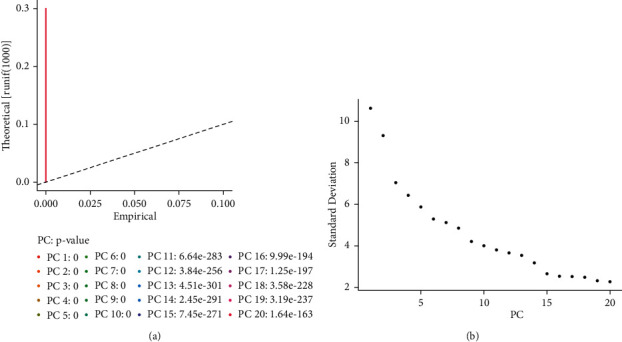
PCA analysis. (a) The difference between the theoretical and actual values of the first 20 PCs. (b) Elbow plot.

Single-cell sequencing results showed that the gene number of the samples was mainly distributed between 1000 and 8000, the gene count was mainly distributed between 100 and 50,000, and the mitochondrial proportion was mainly distributed between 0 and 5% (Figure 1). The correlation between the depth of the sequencing and the number of genes detected was 0.89, and between the sequencing depth and mitochondria was 0.16, indicating a positive correlation of sequencing depth with the number of measured genes (Figure 2). 2000 differentially expressed genes that are highly expressed were selected for principal component analysis (PCA), and the differences between the first 15 PCs were all highly significant, suggesting a considerable difference between theoretical and actual values, which was used for additional analysis (Figure 3).

### 3.2. CD8+ T Cell Extraction and Subpopulation Recognition

**Figure 4:**
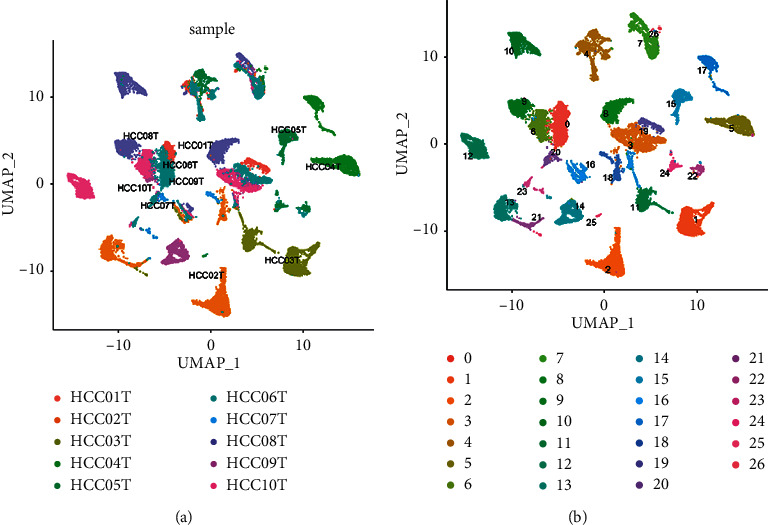
Clustering diagram of cells. (a) Labeled according to the sample name. (b) Labeled according to cluster ID.

**Figure 5:**
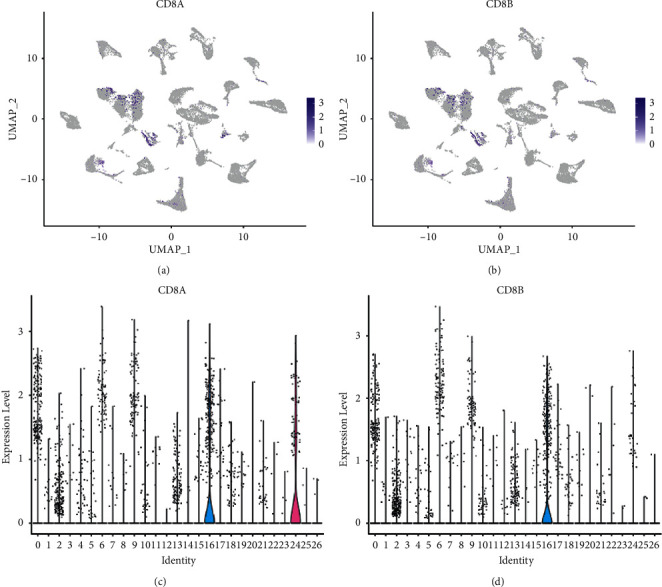
Expression and distribution of CD8+ T cell markers. (a, c) Expression distribution of CD8A. The darker the color, the higher the expression. (b, d) Expression distribution of CD8B.

**Figure 6:**
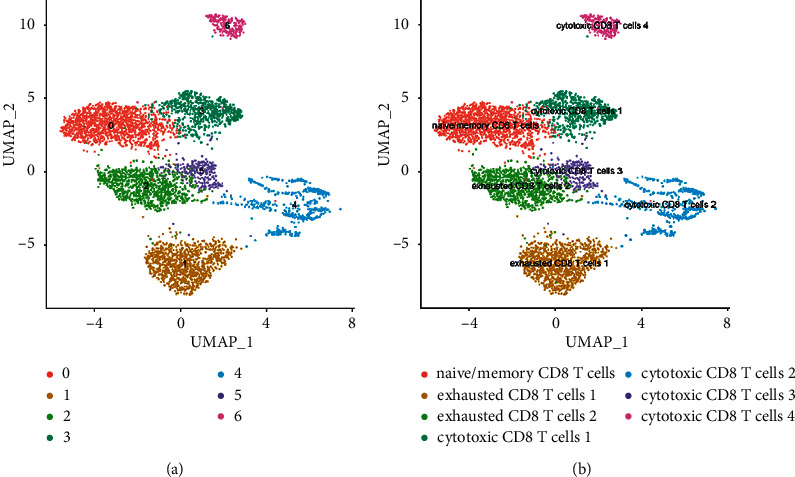
Cluster of CD8+ T cells. (a) An unannotated cluster diagram. (b) A cluster diagram annotated according to CD8+ T subtype markers.

The UMAP method was used for clustering, and 26 clusters were obtained (Figure 4(b)). Because it was a tumor tissue sample, the heterogeneity was high (Figure 4(a)). CD8A and CD8B were mainly distributed in cluster 0, 6, 9, 16, and 24, with a total of 5062 cells (Figure 5). The cells of these groups were pulled out, and UMAP clustering was performed again to obtain 7 CD8+ T subgroups (Figure 6). CD8+ T cell subtype markers were obtained from the research report of Deng et al. [37], that is, markers of naive/memory CD8 T cells: CCR7, IL7R, TCF7, SELL, SATB1, GPR183, LTB, LEF1, and S100A10; the markers of cytotoxic CD8 T cells: PRF1, GZMA, GZMK, and NKG7; and the markers of exhausted CD8 T cells: CXCL13, HSPB1, IRF4, LAYN, GIMAP6, HSPH1, CXCR6, CTLA4, PDCD1, LAG3, HAVCR2, and TIGIT. Based on the expression of the above markers, 7 cell subgroups were annotated as follows: naive/memory CD8 T cells, exhausted CD8 T cells 1, exhausted CD8 T cells 2, cytotoxic CD8 T cells 1, cytotoxic CD8 T cells 2, cytotoxic CD8 T cells 3, and cytotoxic CD8 T cells 4.

### 3.3. Cell Proportion and Cell Marker Expression

The proportion of cells in each subpopulation was shown to be naive/memory CD8 T cells in the HCC TME. The proportion of the medium is the highest, followed by exhausted CD8 T cells 1, and the lowest is cytotoxic CD8 T cells 4 (Figure S1A of the supplementary information file). At the same time, the expression of top 10 differentially expressed genes in seven CD8+ T subsets was analyzed (Figure S1B). According to the transcriptome data of TCGA-LIHC, CIBERSORTx calculated that cytotoxic CD8 T cells 4 and exhausted CD8 T cells 2 accounted for the highest proportion (Figure S8, Table S1).

### 3.4. Analysis of Prognosis and Clinical Correlation

The survival analysis was performed by log-rank test. The survival difference of the overall immune cell proportion group was statistically significant, and there was a significant survival difference between the groups with a high and low proportion of cytotoxic CD8 T cells 4. The survival difference between the exhausted CD8 T cells 2 and naive/memory CD8 T cells was not statistically significant (Figure S2, Table S2). Cytotoxic CD8 T cells 4 differed significantly between the TP53 mutant and nonmutant groups (nonparametric Wilcox rank-sum test) and between sex (Figures S9A and S9B). Among cirrhosis groups of different degrees, the proportion of cytotoxic CD8 T cells 4 increased first and was the lowest in nodular formation and incomplete cirrhosis (Figure S9C). In the grading of liver cancer, the ratio of cytotoxic CD8 T cells 4 was highest in G2 and then decreased progressively (Figure S9D). The proportion of cytotoxic CD8 T cells 4 decreased first and then increased in the stage of liver cancer (Figure S9E). The proportion of cytotoxic CD8 T cells 4 was lowest in the 40–60 years age group (Figure S9F). The proportion of cytotoxic CD8 T cells 4 increased with increasing body weight (Figure S9G).

### 3.5. The Landscape of Heterogeneous Pathway Activity

**Figure 7:**
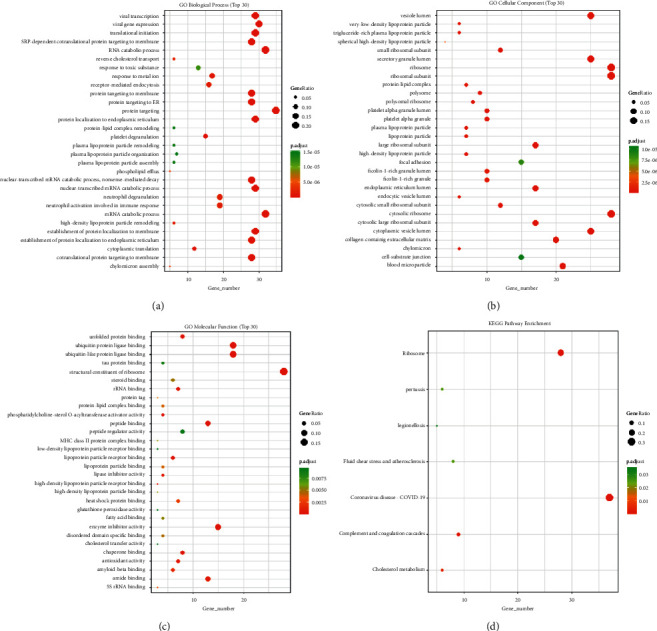
GO and KEGG enrichment analysis of the cytotoxic CD8 T cells 4 subgroup. (a) GO enrichment of biological process. (b) GO enrichment of cellular component. (c) GO enrichment of molecular function. (d) Enrichment of KEGG pathway. The vertical axis represents the enrichment items, the horizontal axis represents the number of different genes in the enrichment items, the size of the dot represents the ratio of the number of different genes in the enrichment items to the number of background genes, and the color represents the BH-corrected *P* value. The red color is directly proportional to enrichment.

**Figure 8:**
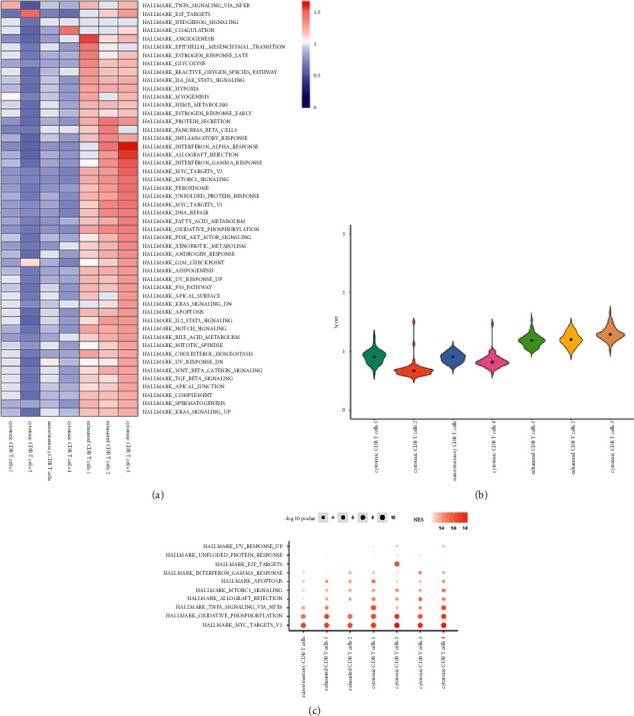
Hallmark pathway activity analysis of CD8+ T subsets. (a) Heatmap of hallmark pathway activity of CD8+ T subsets. (b) Active fiddle diagram of the hallmark pathway in CD8+ T subsets. (c) GSEA enrichment fractional point diagram of the CD8+ T subgroup.

To investigate the presence of heterogeneous pathways in CD8+ T cell subsets, we used GO and KEGG analysis. GO analysis showed that cytotoxic CD8 T cells 4 had an obvious biological process, cellular component, and molecular function (Figures 7(a)–7(c), Table 1). KEGG results showed that cytotoxic CD8 T cells 4 were related to ribosomes (Figure 7(d), Table 1). To elucidate the heterogeneity of various subgroups further, we also performed the analysis of cell subgroup metabolic pathway activity and hallmark immune checkpoint pathway activity. The analysis of the metabolic pathway activity of cell subsets revealed that exhausted CD8 T cells 1, exhausted CD8 T cells 2, and cytotoxic CD8 T cells 3 had strong metabolic pathway activity, and cytotoxic CD8 T cells 2 had the lowest metabolic pathway activity (Figures S3A and S3B). Oxidative phosphorylation was significantly enriched in 7 CD8+ T cell subgroups (Figure S3C). Hallmark pathway activity analysis revealed that exhausted CD8 T cells 1, exhausted CD8 T cells 2, and cytotoxic CD8 T cells 3 had strong hallmark pathway activity, and cytotoxic CD8 T cells 2 had the lowest hallmark pathway activity (Figures 8(a) and 8(b)). HALLMARK_MYC_TARGETS_V1 was significantly enriched in 7 CD8+ T cell subgroups (Figure 8(c)).

### 3.6. Analysis of Cell Differentiation Trajectories and Cell Interaction Networks

The initial differentiation group is not specified and a lineage is obtained from exhausted CD8 T cells 1 to cytotoxic CD8 T cells 4, as shown in Figure S4A. The PRF1, GZMA, and NKG7 genes were first downregulated, then upregulated, and finally downregulated during the development of lineage (Figures S4B–S4E).The PDCD1, HAVCR2, LAG3, CD27, CTLA4, TIGIT, and TNFRSF9 genes remained unchanged during the predifferentiation process and significantly differentiated in the mid-term and then remained unchanged (Figures S4F–S4L).

Considering the heterogeneity of CD8+ T cell subsets, we analyzed their communication networks to identify the key ligand-receptor pairs and cell subsets that dominate the interactions. The results showed that cytotoxic CD8 T cells 2, cytotoxic CD8 T cells 1, and cytotoxic CD8 T cells 4 had the highest number of ligand receptors (Figure S5A). The ligand-receptor logarithm between exhausted CD8 T cells 2 and the other 6 subtypes was less (Figure S5B), and the regulatory factors JUND, EGR1, FOSB, IRF1, IRF8, and REL were highly expressed in cytotoxic CD8 T cells 1 (Figure S5C).

### 3.7. Prognosis/Treatment Marker Identification

**Figure 9:**
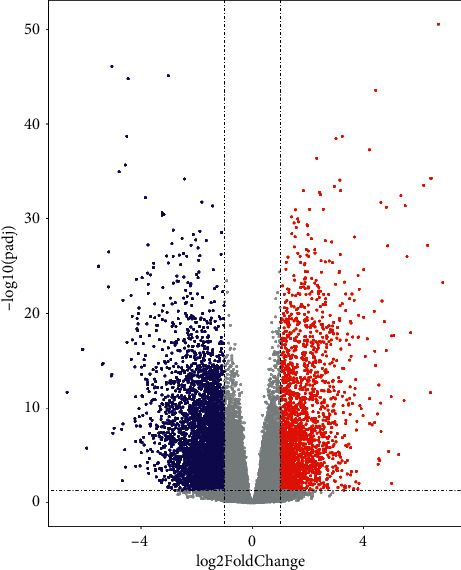
Differential analysis of TCGA-LIHC. (a) Volcanic map for TCGA-LIHC differential analysis. (b) Heatmap of differential gene expression. Differential gene screening criteria: |log2(FoldChange)|>1 and corrected *P* value <0.05.

**Figure 10:**
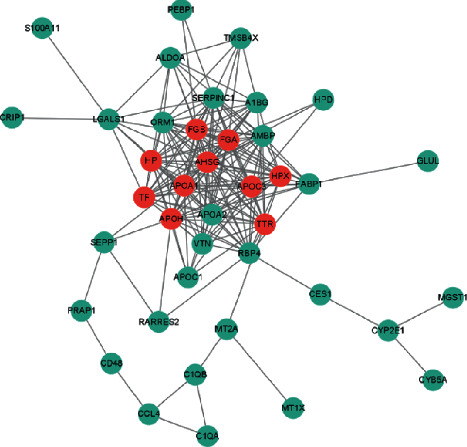
PPI network diagram.

**Figure 11:**
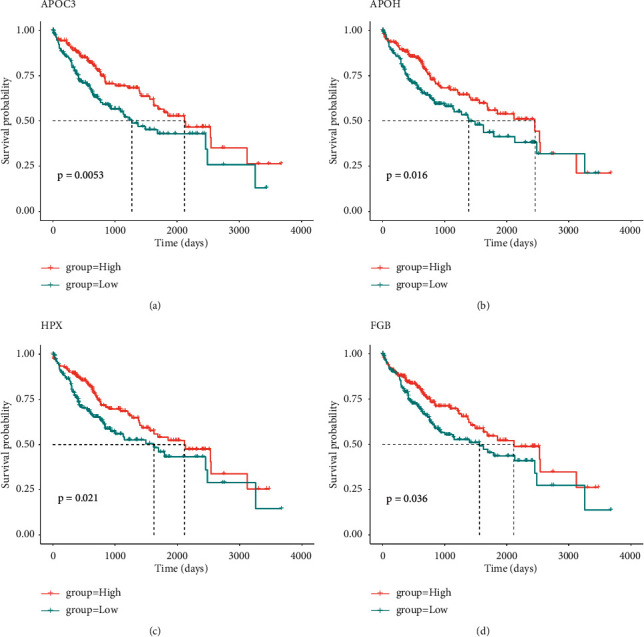
Hub gene survival analysis. (a) KM curve of APOC3 high- and low-expression groups. (b) KM curve of APOH high- and low-expression groups. (c) KM curve of HPX high- and low-expression groups. (d) KM curve of FGB high- and low-expression groups.

Based on the results of significant differences in survival, the differentially expressed genes were calculated first by the TCGA database, and a total of 6813 differentially expressed genes were obtained, of which 2378 were upregulated and 4435 were downregulated (Table S3). The expression of the first 50 differential genes is shown in Figures 9(a) and 9(b). Then, according to the results of significant differences in survival, the corresponding cell subsets and differentially expressed genes were selected, and the ligand receptors and transcription factors among them were given priority. If the subsequent analysis was not supported, only differentially expressed genes were selected, and a total of 168 differentially expressed genes were found. Since the survival analysis of 4 cases of cytotoxic CD8 T cells in the high and low groups showed significant survival differences, the expression of the first 50 differentially expressed genes of cytotoxic CD8 T cells 4 was selected for display (Figure S10). According to the obtained two groups of different genes, the PPI network was constructed and hub genes were identified. cytoHubba found 10 hub genes: FGA, APOA1, APOH, AHSG, FGB, HP, TTR, TF, HPX, and APOC3 (Figure 10). The log-rank test showed that APOC3 among the above 10 hub genes had statistically significant survival differences, while APOH, HPX, and FGB had significant survival differences (Figures 11(a)–11(d)). The Cox test showed that the *P*-values of APOC3, APOH, HPX, and FGB were 0.02, 0.14, 0.00067, and 0.034, respectively. Genetic GSEA of APOC3, APOH, HPX, and FGB was performed, and the first five enrichment items are shown in Figures 11(a)–11(d) and Table S4 in the supplementary file. Table S4 in the supplementary file shows the analysis results of single-gene GSEA of APOC3, APOH, HPX, and FGB. The expression levels of different genes in different clinical features were further analyzed, and the expression levels of genes APOC3, APOH, HPX, and FGB were significantly different in normal tissues and tumors , and were further analyzed whether TP53 mutation was present or not. There was a significant difference (*P*=0.0053) in the expression of APOH among the sex groups. The expressions of APOC3, APOH, HPX, and FGB were not significantly different in different cirrhosis degrees and different ages (*P* > 0.05). There were significant differences in the grading and staging of liver cancer. The gene APOC3 had no significant difference among different body weights (Figures S6 and S11). Finally, the expression of different genes in different cancers was analyzed, and the results showed that the expression of genes APOC3, APOH, HPX, and FGB was the highest in liver hepatocellular carcinoma (LIHC), and the expression in normal tissues was higher than that in cancer tissues. Genes APOC3, ApoH, HPX, and FGB were also highly expressed in cholangiocarcinoma (CHOL), and their expression levels were higher in normal tissues than in cancer tissues (Figure S12).

## 4. Discussion

The role of tumor-infiltrating immune cells, particularly T cells, in tumor development has been revolutionized by a deeper understanding that has opened up new avenues for immunotherapeutic strategies. Previous studies have indicated that immune cells infiltrating tumors exhibit various levels of infiltration depending on the type of tumor and stages [39]. Immune-associated cells, including T cells and mast cells, have been shown to be novel prognostic markers in patients with HCC, further suggesting that the combination of immunoinfiltrating cells in tumor tissue can even predict the effects of chemotherapy and immunotherapy [40]. Due to the fact that CD8+ T cells are the most significant effector T cells in current tumor immunotherapy [41], CD8+ T cells detect tumor-associated antigens on the surface of cancer cells as major histocompatibility complex class I molecules [42].

It has been found that four coexpression genes (GZMA, C1QC, CD3D, and PSMB9) have been identified as CD8+ T cell coexpression genes that promote CD8+ T cell infiltration in HCC, and these coexpressed genes are favorably associated with the infiltration of CD8+ T lymphocytes during antigen presentation. This biological process may provide new directions for patients with stem cell cancers that are not sensitive to immunotherapy [43]. Thus, CD8+ T cells are essential for the formation of antitumor immunity, and their increased invasion is related with a favorable prognosis. CD8+ T cell subsets in the microenvironment of the tumor, on the other hand, may play distinct roles in tumor progression, prognosis, and immunotherapy. Cytotoxic CD8+ T cells have been reported to be associated with lymph node metastasis and other prognostic factors in breast cancer [44]. We found that cytotoxic CD8 T cells 4 differed significantly between the TP53-mutated and nonmutated groups, as well as with different degrees of cirrhosis, HCC grade, stage, age, and body weight. Cytotoxic CD8 T cells 4 differential genes were analyzed by the TCGA-LIHC data and single-cell sequencing data set. Finally, 10 hub genes were found: FGA, ApoA1, ApoH, AHSG, FGB, HP, TTR, TF, HPX, and APOC3. There were significant survival differences among APOC3, APOH, HPX, and FGB genes. Further analysis showed that APOC3, APOH, HPX, and FGB were significantly different in normal tissues and tumors irrespective of TP53 mutation, liver cancer grade, and stage. There was a significant difference in the expression of APOH among the sex groups. APOC3, APOH, HPX, and FGB expression levels were highest in HCC and were greater in normal tissues than that in cancer tissues. Additionally, it is significantly expressed in CHOL, and its level of expression is higher in normal tissues than that in cancer tissues. Apoprotein C3 (APOC3) is a key regulator of the metabolism of lipoprotein and has been demonstrated to be closely associated with hypertriglyceridemia [45]. *β*-2-glycoprotein 1 (APOH) has been shown to be associated with liver metastasis from colorectal cancer [46]. Hemopexin (HPX), which acts as a scavenger of toxic plasma heme and a transporter of it to the liver, has been demonstrated to be closely associated to the occurrence and development of breast cancer [47, 48]. Similarly, the fibrinogen *β* chain (FGB) gene has been revealed to be related with renal cell carcinoma invasion and metastasis [49]. All of these provide strong evidence that APOC3, APOH, HPX, and FGB can be used as biomarkers for hepatocellular carcinoma.

Tumor immunotherapy is a new method to treat cancer in recent years, which has greatly changed the prospect of cancer treatment [50, 51]. Although significant advances can be made in treatments such as immune checkpoint blockade, their efficacy varies greatly among different patients or cancer types [52]. A detailed understanding of the internal immune microenvironment of cancer tissue is of great reference value for the development of new immunotherapy. Single-cell sequencing technology can be used as an effective tool to study the immune microenvironment of liver cancer and plays an essential role in the process of immune cell therapy and antibody drug development of liver cancer.

The incidence and mortality of liver cancer are high [53, 54]. In order to understand the immune microenvironment of liver cancer and to find new targets and effective biomarkers for the immunotherapy of liver cancer, Zheng et al. [17] performed sRNA-seq on 5063 human T cells using the SMART Seq2 technique. Subpopulation classification of T cells based on single-cell transcriptional map showed that there were a large number of dysfunctional lethal CD8+ T cells and inhibitory T cells in tumor tissues. The gene Layilin was found to inhibit the killing function of CD8+ T cells by targeting the genes specifically expressed in these two types of cells, which may be a new potential target for immunotherapy.

## 5. Conclusion

Immune-associated cells, including T cells, have been shown to be novel prognostic markers in patients with HCC, suggesting that the combination of immunoinfiltrating cells in tumor tissue can even predict the effects of chemotherapy and immunotherapy. Because CD8+ T cells are the most important effector T cells in the current tumor immunotherapy and they also recognize tumor-associated antigens as major histocompatibility complex class I molecules on the surface of cancer cells, we used scRNA-seq data of hepatocellular carcinoma (HCC) to reveal the heterogeneity of different CD8+ T cell subsets.CD8+ T cells in HCC were divided into 7 subsets, and the subset cytotoxic CD8 T cells 4 showed significant differences between the TP53 mutant group and the nonmutant group, as well as between different degrees of cirrhosis, HCC grades, stages, ages, and body weights. Hub genes were identified by TCGA-LIHC and single-cell sequencing data set analysis, and the genes APOC3, APOH, HPX, and FGB were identified as biological marker genes by the Cox test. The expression of APOC3, APOH, HPX, and FGB in normal tissues and tumors and TP53 mutation were significantly different. There was a significant difference in the expression of APOH among the sex groups. There were significant differences in the grading and staging of liver cancer. The gene APOC3 had no significant difference among different body weights. The expression levels of APOC3, APOH, HPX, and FGB were the highest in HCC and were higher in normal tissues than in cancer tissues. Moreover, it is also highly expressed in CHOL, and the expression level in normal tissues is higher than that in cancer tissues. We found that cytotoxic CD8 T cells 4 is closely associated with survival and prognosis of HCC and identified four differential genes that can be used as biological markers for survival, prognosis, and clinically relevant characteristics of HCC. This study could help to find effective targets for immunotherapy of HCC and accelerate the development of immunotherapy for HCC. At the same time, this work also outlines the map of the tumor-immune environment, which provides a basis for the future study of other tumor-immune microenvironments.

## Figures and Tables

**Table 1:** GO analysis and genomic encyclopedia (KEGG) analysis of cytotoxic CD8 T cells 4.

ID	Description	Gene ratio	Bg ratio	*P* value	p.adjust	*Q* value	Gene ID	Count
hsa05171	Coronavirus disease—COVID-19	37/113	232/8105	5.79*E* − 30	1.28*E* − 27	1.11*E* − 27	2243/2244/718/2266/6189/712/4792/713/6173/6206/6158/6137/6171/6224/6143/6156/6181/6187/6139/5295/6202/7311/6161/3725/6192/6134/6135/6130/6159/6167/6228/6218/6133/6205/3921/6125/51065	37
hsa03010	Ribosome	28/113	158/8105	7.35*E* − 24	8.12*E* − 22	7.08*E* − 22	6189/6173/6206/6158/6137/6171/6224/6143/6156/6181/6187/6139/6202/7311/6161/6192/6134/6135/6130/6159/6167/6228/6218/6133/6205/3921/6125/51065	28
hsa04610	Complement and coagulation cascades	9/113	85/8105	2.45*E* − 06	0.000181	0.000158	2243/462/2244/7448/718/2266/712/713/1191	9
hsa04979	Cholesterol metabolism	6/113	50/8105	6.20*E* − 05	0.003425	0.002985	350/345/335/341/348/336	6
hsa05133	Pertussis	6/113	76/8105	0.000636	0.025371	0.022114	718/712/713/805/3725/3659	6
hsa05418	Fluid shear stress and atherosclerosis	8/113	139/8105	0.000689	0.025371	0.022114	2938/4257/1843/805/5295/3725/3326/3320	8
hsa05134	Legionellosis	5/113	57/8105	0.001133	0.035775	0.031183	718/4792/1915/3329/3312	5

## Data Availability

All data generated or analyzed during this study are included in this article and provided in the supplementary file.

## Abbreviations

CHOL: Cholangiocarcinoma
CTLA4: Cytotoxic T-lymphocyte-associated protein 4
FDA: Food and Drug Administration
GO: Gene Ontology
GSEA: Gene set enrichment analysis
HCC: Hepatocellular carcinoma
KEGG: Kyoto Encyclopedia of Genes and Genomes
LIHC: Liver hepatocellular carcinoma
NSCLC: Non-small-cell lung cancer
TACE: Transcatheter hepatic arterial chemoembolization
TSA: Tumor-specific antigen
MHC: Major histocompatibility complex
PD1: Programmed cell death protein 1
PCA: Principal component analysis
PPI: Protein-protein interaction
TIM-3: T-cell immunoglobulin mucin-3
TME: Tumor microenvironment
TCR: T cell receptor.

## Supplementary Materials

Supplementary Materials Table S1: cell proportion and cell marker expression. Table S2: analysis of prognosis and clinical correlation. Table S3: prognosis/treatment marker identification. Table S4: genetic GSEA of APOC3, APOH, HPX, and FGB. Figure S1: the proportion and marker expression of CD8+ T subtypes. (A) Proportion bar chart of 7 CD8+ T subtypes. (B) Heat maps of 7 types of CD8+ T subtype markers. Figure S2: cell subpopulation survival analysis. (A) The KM curve of the whole immune cell proportion grouping. (B) The KM curves of cytotoxic CD8 T cells 4. (C) The KM curves of exhausted CD8 T cells 2. (D) The KM curves of naive/memory CD8 T cells. Figure S3: metabolic pathway activity analysis of CD8+ T subsets. (A) Heatmap of metabolic pathway activity of CD8+ T subsets. (B) Violin diagram of metabolic pathway activity of CD8+ T subsets. (C) GSEA enrichment fractional point diagram of CD8+ T subgroups. Figure S4: cell differentiation trajectory analysis and marker gene distribution. (A) Cell differentiation trajectories of seven CD8+ T subtypes. (B)–(L) Expression profile of marker genes in Lineage 1. Figure S5: cell-cell interaction network analysis. (A) Cell interaction network diagram. (B) Heatmap of ligand-receptor pairs between seven subgroups. (C) Regulators of seven subgroups. Figure S6: expression of genes HPX and FGB under different clinical characteristics. (A)–(H) Violin diagrams showing the expression differences of genes HPX (left) and FGB (right) in normal and tumor tissues, TP53-mutant and nonmutant patients, gender, degree of liver cirrhosis, cancer grade, cancer stage, different ages, and different body weights; the degree of liver cirrhosis was divided into no fibrosis, portal fibrosis, nodular formation and incomplete cirrhosis, and established cirrhosis; age: 0–20, 20–40, 40–60, 60–80 and 80–100; body weight: 40–60, 60–80, 80–100, 100–150, and 150–200. Figure S7: single-gene GSEA of hub genes. (A) APOC3 single-gene GSEA, showing the first 5 enrichment pathways. (B) ApoH single-gene GSEA, showing the first 5 enrichment pathways. (C) HPX single-gene GSEA, showing the first 5 enrichment pathways. (D) FGB single-gene GSEA, showing the first 5 enrichment pathways. Figure S8: proportion of TCGA-LIHC immune cells. (A) Boxplot of the proportion of cells of the seven CD8+ T subtypes. (B) Heatmap of the proportion of the seven CD8+ T subtypes. Figure S9: proportion of cytotoxic CD8 T cells 4 in different clinical features. (A), (B) Boxplots showing the ratio of CD8 T cells 4 with or without TP53 mutation and sex, respectively. (C)–(G) Line plots showing the proportion of cytotoxic CD8 T cells 4 in cirrhosis degree, grade, stage, age, and body weight. Figure S10: differential analysis of CD8+ T subsets. Figure S11: expression of genes APOC3 and APOH in different clinical characteristics. (A)–(H) Violin diagrams showing the expression differences of APOC3 (left) and APOH (right) in normal and tumor tissues, TP53-mutant and nonmutant patients, gender, degree of liver cirrhosis, cancer grade, cancer stage, different ages, and different body weights; the degree of liver cirrhosis was divided into no fibrosis, portal fibrosis, nodular formation and incomplete cirrhosis, and established cirrhosis; age: 0–20, 20–40, 40–60, 60–80 and 80–100; body weight: 40–60, 60–80, 80–100, 100–150, and 150–200. Figure S12: hub gene expression in different cancer types. (A) Expression of APOC3 in different cancer types. (B) Expression of ApoH in different types of cancer. (C) Expression of HPX in different types of cancer. (D) Expression of FGB in different cancer types.
