# Supplementary material for: Single-Cell Sequencing Identifies the Heterogeneity of CD8+ T Cells and Novel Biomarker Genes in Hepatocellular Carcinoma
Source: J Healthc Eng. 2022 Apr 12;2022:8256314. doi: 10.1155/2022/8256314 (PMC9018173; doi:10.1155/2022/8256314)
Supplement: Supplementary Materials — Table S1: cell proportion and cell marker expression. Table S2: analysis of prognosis and clinical correlation. Table S3: prognosis/treatment marker identification. Table S4: genetic GSEA of APOC3, APOH, HPX, and FGB. Figure S1: the proportion and marker expression of CD8+ T subtypes. (A) Proportion bar chart of 7 CD8+ T subtypes. (B) Heat maps of 7 types of CD8+ T subtype markers. Figure S2: cell subpopulation survival analysis. (A) The KM curve of the whole immune cell proportion grouping. (B) The KM curves of cytotoxic CD8 T cells 4. (C) The KM curves of exhausted CD8 T cells 2. (D) The KM curves of naive/memory CD8 T cells. Figure S3: metabolic pathway activity analysis of CD8+ T subsets. (A) Heatmap of metabolic pathway activity of CD8+ T subsets. (B) Violin diagram of metabolic pathway activity of CD8+ T subsets. (C) GSEA enrichment fractional point diagram of CD8+ T subgroups. Figure S4: cell differentiation trajectory analysis and marker gene distribution. (A) Cell differentiation trajectories of seven CD8+ T subtypes. (B)–(L) Expression profile of marker genes in Lineage 1. Figure S5: cell-cell interaction network analysis. (A) Cell interaction network diagram. (B) Heatmap of ligand-receptor pairs between seven subgroups. (C) Regulators of seven subgroups. Figure S6: expression of genes HPX and FGB under different clinical characteristics. (A)–(H) Violin diagrams showing the expression differences of genes HPX (left) and FGB (right) in normal and tumor tissues, TP53-mutant and nonmutant patients, gender, degree of liver cirrhosis, cancer grade, cancer stage, different ages, and different body weights; the degree of liver cirrhosis was divided into no fibrosis, portal fibrosis, nodular formation and incomplete cirrhosis, and established cirrhosis; age: 0–20, 20–40, 40–60, 60–80 and 80–100; body weight: 40–60, 60–80, 80–100, 100–150, and 150–200. Figure S7: single-gene GSEA of hub genes. (A) APOC3 single-gene GSEA, showing the first 5 enrichment pathways. (B) [file 8256314.f1.docx]

**Single-Cell Sequencing Identify** **the Heterogeneity of CD8+T Cells and Novel Biomarker Genes in Hepatocellular Carcinoma**

Hailei Wang^1^, Yang Fu^2^, Bin-Bin Da^3^, Geng Xiong^4*^

***Supplementary Materials***

**Table S1.** Cell proportion and cell marker expression

| Mixture | naive/memory CD8 T cells | exhausted CD8 T cells 1 | exhausted CD8 T cells 2 | cytotoxic CD8 T cells 1 | cytotoxic CD8 T cells 2 |  | cytotoxic CD8 T cells 3 | cytotoxic CD8 T cells 4 | P-value | Correlation | RMSE |
| --- | --- | --- | --- | --- | --- | --- | --- | --- | --- | --- | --- |
| TCGA-DD-A4NG-01A | 0 | 0 | 0.291202 | 0 | 0 |  | 0 | 0.708798 | 0.378 | 0.071275 | 1.066314 |
| TCGA-G3-AAV4-01A | 0 | 0 | 0.025874 | 0 | 0 |  | 0.000895 | 0.973231 | 0.366 | 0.081085 | 1.085854 |
| TCGA-2Y-A9H1-01A | 0 | 0 | 0.298497 | 0 | 0 |  | 0 | 0.701503 | 0.342 | 0.097389 | 1.054657 |
| TCGA-CC-A3M9-01A | 0.285245 | 0 | 0.339022 | 0.052287 | 0.308209 |  | 0.015237 | 0 | 0.015 | 0.690601 | 0.754421 |
| TCGA-K7-AAU7-01A | 0 | 0 | 0.308202 | 0 | 0.047132 |  | 0.003005 | 0.641661 | 0.411 | 0.049703 | 1.073196 |
| TCGA-BC-A10W-01A | 0 | 0 | 0.362017 | 0 | 0.152848 |  | 0 | 0.485135 | 0.505 | 0.01087 | 1.084913 |
| TCGA-DD-AACV-01A | 0 | 0 | 0.292337 | 0 | 0.034779 |  | 0 | 0.672883 | 0.35 | 0.092291 | 1.05545 |
| TCGA-DD-AAD3-01A | 0 | 0 | 0.343982 | 0 | 0 |  | 0.002931 | 0.653087 | 0.288 | 0.13481 | 1.037393 |
| TCGA-DD-AACA-01A | 0 | 0 | 0.104664 | 0 | 0 |  | 0.009218 | 0.886118 | 0.377 | 0.071661 | 1.083793 |
| TCGA-G3-AAV2-01A | 0 | 0 | 0.293725 | 0 | 0 |  | 0 | 0.706275 | 0.405 | 0.052234 | 1.074324 |
| TCGA-DD-AACT-01A | 0 | 0 | 0.35542 | 0 | 0 |  | 0 | 0.64458 | 0.394 | 0.05817 | 1.068722 |
| TCGA-GJ-A6C0-01A | 0 | 0 | 0.290391 | 0 | 0.018195 |  | 0.005852 | 0.685562 | 0.291 | 0.13275 | 1.040094 |
| TCGA-CC-5258-01A | 0 | 0 | 0.311317 | 0 | 0.05142 |  | 0 | 0.637263 | 0.368 | 0.077733 | 1.060165 |
| TCGA-QA-A7B7-01A | 0 | 0 | 0.410457 | 0 | 0.120389 |  | 0 | 0.469154 | 0.449 | 0.034949 | 1.073874 |
| TCGA-2Y-A9H3-01A | 0.437199 | 0 | 0.462452 | 0.016014 | 0.006116 |  | 0 | 0.078219 | 0.271 | 0.150154 | 1.026613 |
| TCGA-DD-AACW-01A | 0 | 0 | 0.317237 | 0 | 0.002594 |  | 0 | 0.680169 | 0.249 | 0.17008 | 1.021974 |
| TCGA-ZS-A9CD-01A | 0 | 0 | 0.294892 | 0 | 0 |  | 0.012982 | 0.692126 | 0.382 | 0.067744 | 1.071412 |
| TCGA-UB-A7MF-01A | 0.032998 | 0 | 0.564665 | 0 | 0.020618 |  | 0 | 0.381718 | 0.369 | 0.075094 | 1.056491 |
| TCGA-G3-A5SI-01A | 0 | 0 | 0.366619 | 0 | 0 |  | 0 | 0.633381 | 0.32 | 0.109858 | 1.046694 |
| TCGA-CC-A8HV-01A | 0 | 0 | 0.285917 | 0 | 0.006113 |  | 0.00265 | 0.70532 | 0.24 | 0.180414 | 1.018708 |
| TCGA-DD-AADQ-01A | 0 | 0 | 0.311743 | 0 | 0 |  | 0 | 0.688257 | 0.434 | 0.040561 | 1.078227 |
| TCGA-2Y-A9H6-01A | 0 | 0 | 0.298705 | 0 | 0 |  | 0.002553 | 0.698743 | 0.308 | 0.119156 | 1.045797 |
| TCGA-DD-A1EA-01A | 0 | 0 | 0.382742 | 0 | 0 |  | 0 | 0.617258 | 0.54 | -0.00289 | 1.092453 |
| TCGA-5R-AA1C-01A | 0 | 0 | 0.094897 | 0 | 0 |  | 0 | 0.905103 | 0.475 | 0.02429 | 1.104335 |
| TCGA-DD-A113-01A | 0 | 0 | 0.373941 | 0 | 0 |  | 0 | 0.626059 | 0.306 | 0.120947 | 1.04182 |
| TCGA-2Y-A9H9-01A | 0 | 0 | 0.390385 | 0 | 0 |  | 0 | 0.609615 | 0.371 | 0.074219 | 1.060852 |
| TCGA-CC-A7IE-01A | 0 | 0 | 0.377745 | 0 | 0 |  | 0.008946 | 0.613309 | 0.372 | 0.073415 | 1.064096 |
| TCGA-XR-A8TD-01A | 0.265799 | 0 | 0.449566 | 0 | 0.051591 |  | 0 | 0.233045 | 0.479 | 0.022502 | 1.06556 |
| TCGA-DD-AAD2-01A | 0 | 0 | 0.308475 | 0 | 0 |  | 0 | 0.691525 | 0.388 | 0.062903 | 1.068953 |
| TCGA-DD-AACY-01A | 0 | 0 | 0.292336 | 0 | 0 |  | 0.001919 | 0.705745 | 0.372 | 0.072709 | 1.066164 |
| TCGA-ED-A4XI-01A | 0 | 0 | 0.358899 | 0 | 0 |  | 0 | 0.641101 | 0.31 | 0.115883 | 1.044369 |
| TCGA-CC-A9FW-01A | 0 | 0 | 0.302769 | 0 | 0 |  | 0.002603 | 0.694627 | 0.36 | 0.083619 | 1.06109 |
| TCGA-DD-A4NP-01A | 0 | 0 | 0.316652 | 0 | 0 |  | 0 | 0.683348 | 0.367 | 0.078016 | 1.062105 |
| TCGA-G3-A5SJ-01A | 0.453438 | 0 | 0.352594 | 0 | 0.193968 |  | 0 | 0 | 0.385 | 0.063848 | 1.062362 |
| TCGA-DD-AAE2-01A | 0 | 0 | 0.427113 | 0 | 0.021911 |  | 0 | 0.550976 | 0.404 | 0.05292 | 1.067713 |
| TCGA-DD-AAEK-01A | 0 | 0 | 0.280896 | 0 | 0 |  | 0 | 0.719104 | 0.387 | 0.063204 | 1.070389 |
| TCGA-EP-A2KC-01A | 0 | 0 | 0.328115 | 0 | 0 |  | 0 | 0.671885 | 0.346 | 0.095557 | 1.05411 |
| TCGA-2Y-A9GS-01A | 0 | 0 | 0.379422 | 0 | 0.052754 |  | 0 | 0.567824 | 0.309 | 0.118152 | 1.041077 |
| TCGA-DD-A4NE-01A | 0 | 0 | 0.393096 | 0 | 0.038174 |  | 0 | 0.56873 | 0.393 | 0.058595 | 1.065699 |
| TCGA-DD-AADC-01A | 0 | 0 | 0.305899 | 0 | 0.019782 |  | 0 | 0.674318 | 0.271 | 0.152028 | 1.029546 |
| TCGA-DD-AAVZ-01A | 0 | 0 | 0.290111 | 0 | 0 |  | 0.00349 | 0.706399 | 0.288 | 0.135689 | 1.039096 |
| TCGA-CC-A3MA-01A | 0 | 0 | 0.311874 | 0 | 0.058832 |  | 0 | 0.629294 | 0.329 | 0.105256 | 1.048195 |
| TCGA-2Y-A9GY-01A | 0.744956 | 0 | 0.123773 | 0 | 0.102259 |  | 0.029012 | 0 | 0.276 | 0.142103 | 1.058643 |
| TCGA-ED-A627-01A | 0.051891 | 0 | 0.326634 | 0 | 0 |  | 0 | 0.621476 | 0.49 | 0.017179 | 1.081461 |
| TCGA-2Y-A9GW-01A | 0 | 0 | 0.278889 | 0 | 0 |  | 0.017376 | 0.703735 | 0.383 | 0.067107 | 1.07399 |
| TCGA-DD-AADO-01A | 0 | 0 | 0.339702 | 0 | 0 |  | 0 | 0.660298 | 0.319 | 0.110454 | 1.047302 |
| TCGA-DD-AADP-01A | 0 | 0 | 0.30397 | 0 | 0 |  | 0.004271 | 0.691759 | 0.303 | 0.122943 | 1.044331 |
| TCGA-DD-AADK-01A | 0 | 0 | 0.330555 | 0 | 0 |  | 0 | 0.669445 | 0.272 | 0.147942 | 1.031425 |
| TCGA-G3-AAV7-01A | 0 | 0 | 0.320761 | 0 | 0.286442 |  | 0 | 0.392798 | 0.412 | 0.049239 | 1.075418 |
| TCGA-DD-A1EI-01A | 0 | 0 | 0.325252 | 0 | 0 |  | 0 | 0.674748 | 0.247 | 0.174896 | 1.019742 |
| TCGA-DD-AACA-02A | 0 | 0 | 0.279657 | 0 | 0 |  | 0 | 0.720343 | 0.349 | 0.092918 | 1.057574 |
| TCGA-KR-A7K0-01A | 0 | 0 | 0.17021 | 0 | 0 |  | 0 | 0.82979 | 0.368 | 0.077728 | 1.072075 |
| TCGA-DD-AACC-01A | 0 | 0 | 0.466229 | 0 | 0 |  | 0.00781 | 0.525961 | 0.632 | -0.03047 | 1.103078 |
| TCGA-BC-A3KG-01A | 0 | 0 | 0.33077 | 0 | 0.078345 |  | 0 | 0.590885 | 0.475 | 0.024373 | 1.080767 |
| TCGA-G3-AAV1-01A | 0 | 0 | 0.203383 | 0 | 0 |  | 0 | 0.796617 | 0.372 | 0.072691 | 1.071614 |
| TCGA-CC-A7IK-01A | 0 | 0 | 0.278153 | 0 | 0 |  | 0 | 0.721847 | 0.322 | 0.108818 | 1.050689 |
| TCGA-G3-A25S-01A | 0 | 0 | 0.19414 | 0 | 0.003794 |  | 0.009441 | 0.792625 | 0.283 | 0.138944 | 1.044141 |
| TCGA-DD-AADR-01A | 0 | 0 | 0.290406 | 0 | 0 |  | 0 | 0.709594 | 0.314 | 0.111904 | 1.048726 |
| TCGA-UB-A7MD-01A | 0 | 0 | 0.287507 | 0 | 0 |  | 0 | 0.712493 | 0.384 | 0.065251 | 1.069115 |
| TCGA-DD-A4NS-01A | 0 | 0 | 0.307399 | 0 | 0 |  | 0 | 0.692601 | 0.36 | 0.084021 | 1.059985 |
| TCGA-DD-AAE3-01A | 0 | 0 | 0.236681 | 0 | 0 |  | 0.008282 | 0.755037 | 0.366 | 0.081173 | 1.067687 |
| TCGA-DD-AACP-01A | 0 | 0 | 0.311006 | 0 | 0.007382 |  | 0 | 0.681612 | 0.272 | 0.148045 | 1.031667 |
| TCGA-DD-A115-01A | 0 | 0 | 0.311843 | 0 | 0.076691 |  | 0 | 0.611466 | 0.391 | 0.060409 | 1.066683 |
| TCGA-2Y-A9H8-01A | 0 | 0 | 0.300977 | 0 | 0.045004 |  | 0 | 0.654019 | 0.303 | 0.123173 | 1.041342 |
| TCGA-FV-A3I1-01A | 0 | 0 | 0.237779 | 0 | 0.003097 |  | 0.005613 | 0.753512 | 0.275 | 0.143957 | 1.03826 |
| TCGA-2Y-A9H7-01A | 0 | 0 | 0.290947 | 0 | 0.016069 |  | 0 | 0.692983 | 0.292 | 0.131964 | 1.039092 |
| TCGA-DD-AAVU-01A | 0 | 0 | 0.307141 | 0 | 0 |  | 0.009438 | 0.683421 | 0.306 | 0.121275 | 1.046245 |
| TCGA-BC-A110-01A | 0 | 0 | 0.284017 | 0 | 0 |  | 0 | 0.715983 | 0.389 | 0.061847 | 1.070785 |
| TCGA-ZP-A9CZ-01A | 0.104375 | 0 | 0.26103 | 0 | 0.055735 |  | 0 | 0.57886 | 0.433 | 0.04098 | 1.068397 |
| TCGA-DD-AAE1-01A | 0 | 0 | 0.310895 | 0 | 0 |  | 0 | 0.689105 | 0.271 | 0.150971 | 1.030731 |
| TCGA-GJ-A3OU-01A | 0.032367 | 0 | 0.256584 | 0 | 0.006832 |  | 0 | 0.704217 | 0.382 | 0.067714 | 1.066087 |
| TCGA-DD-A4NK-01A | 0 | 0 | 0.313175 | 0 | 0 |  | 0 | 0.686825 | 0.353 | 0.088923 | 1.057606 |
| TCGA-RC-A7SB-01A | 0 | 0 | 0.313713 | 0 | 0 |  | 0 | 0.686287 | 0.322 | 0.108835 | 1.049009 |
| TCGA-FV-A3I0-01A | 0 | 0 | 0.316658 | 0 | 0.503335 |  | 0 | 0.180007 | 0.299 | 0.126566 | 1.062486 |
| TCGA-ED-A66X-01A | 0 | 0 | 0.342296 | 0 | 0 |  | 0 | 0.657704 | 0.27 | 0.153099 | 1.028852 |
| TCGA-2Y-A9GT-01A | 0 | 0 | 0.352534 | 0 | 0 |  | 0 | 0.647466 | 0.354 | 0.0881 | 1.05632 |
| TCGA-FV-A2QQ-01A | 0 | 0 | 0.291658 | 0 | 0 |  | 0 | 0.708342 | 0.301 | 0.126025 | 1.042472 |
| TCGA-DD-AAEH-01A | 0 | 0 | 0.194775 | 0 | 0 |  | 0 | 0.805225 | 0.303 | 0.124449 | 1.048719 |
| TCGA-G3-A3CK-01A | 0 | 0 | 0.276668 | 0 | 0 |  | 0 | 0.723332 | 0.449 | 0.034405 | 1.083009 |
| TCGA-EP-A26S-01A | 0 | 0 | 0.293799 | 0 | 0 |  | 0 | 0.706201 | 0.372 | 0.073505 | 1.065211 |
| TCGA-DD-A11A-01A | 0 | 0 | 0.308494 | 0 | 0 |  | 0 | 0.691506 | 0.294 | 0.13009 | 1.039983 |
| TCGA-DD-AAE6-01A | 0 | 0 | 0.309524 | 0 | 0 |  | 0 | 0.690476 | 0.293 | 0.130481 | 1.039772 |
| TCGA-XR-A8TE-01A | 0 | 0 | 0.316629 | 0 | 0 |  | 0.003677 | 0.679694 | 0.4 | 0.055595 | 1.072685 |
| TCGA-DD-A73B-01A | 0 | 0 | 0.28176 | 0 | 0 |  | 0.014159 | 0.704081 | 0.323 | 0.107848 | 1.054692 |
| TCGA-EP-A3JL-01A | 0 | 0 | 0.298158 | 0 | 0 |  | 0.01335 | 0.688492 | 0.368 | 0.075959 | 1.067744 |
| TCGA-CC-A3MB-01A | 0 | 0 | 0.294402 | 0 | 0.023906 |  | 0 | 0.681693 | 0.288 | 0.135579 | 1.037014 |
| TCGA-DD-AAD0-01A | 0 | 0 | 0.274854 | 0 | 0 |  | 0 | 0.725146 | 0.368 | 0.076719 | 1.064902 |
| TCGA-MI-A75I-01A | 0 | 0 | 0.281337 | 0 | 0 |  | 0.015152 | 0.70351 | 0.271 | 0.149107 | 1.036288 |
| TCGA-CC-A8HU-01A | 0 | 0 | 0.307355 | 0 | 0.062689 |  | 0 | 0.629955 | 0.313 | 0.112941 | 1.044952 |
| TCGA-RC-A6M5-01A | 0 | 0 | 0.433093 | 0 | 0 |  | 0 | 0.566907 | 0.478 | 0.022721 | 1.080435 |
| TCGA-2Y-A9GZ-01A | 0 | 0 | 0.379453 | 0 | 0.030323 |  | 0 | 0.590223 | 0.384 | 0.064878 | 1.063778 |
| TCGA-DD-AACO-01A | 0 | 0 | 0.372937 | 0 | 0 |  | 0 | 0.627063 | 0.33 | 0.104769 | 1.048657 |
| TCGA-LG-A6GG-01A | 0 | 0 | 0.181009 | 0 | 0.039776 |  | 0 | 0.779215 | 0.408 | 0.050315 | 1.080877 |
| TCGA-ED-A7PZ-01A | 0 | 0 | 0.281692 | 0 | 0 |  | 0 | 0.718308 | 0.236 | 0.184229 | 1.016872 |
| TCGA-DD-A4NV-01A | 0 | 0 | 0.294274 | 0 | 0 |  | 0 | 0.705726 | 0.291 | 0.133333 | 1.03914 |
| TCGA-DD-A73G-01A | 0 | 0 | 0.31763 | 0 | 0 |  | 0 | 0.68237 | 0.294 | 0.130461 | 1.039473 |
| TCGA-G3-AAV3-01A | 0 | 0 | 0.322792 | 0 | 0 |  | 0.017846 | 0.659362 | 0.834 | -0.08581 | 1.136447 |
| TCGA-BC-A5W4-01A | 0 | 0 | 0.300568 | 0 | 0 |  | 0 | 0.699432 | 0.247 | 0.173773 | 1.020954 |
| TCGA-UB-AA0U-01A | 0 | 0 | 0.338424 | 0 | 0 |  | 0 | 0.661576 | 0.289 | 0.134483 | 1.037024 |
| TCGA-EP-A12J-01A | 0 | 0 | 0.285501 | 0 | 0 |  | 0 | 0.714499 | 0.357 | 0.08582 | 1.060344 |
| TCGA-WJ-A86L-01A | 0 | 0 | 0.208912 | 0 | 0.005368 |  | 0.008963 | 0.776756 | 0.261 | 0.160272 | 1.03299 |
| TCGA-DD-AACA-02B | 0 | 0 | 0.278571 | 0 | 0 |  | 0.015973 | 0.705455 | 0.391 | 0.060245 | 1.076602 |
| TCGA-FV-A4ZQ-01A | 0.207967 | 0 | 0.435944 | 0 | 0 |  | 0 | 0.356089 | 0.575 | -0.01549 | 1.080056 |
| TCGA-WX-AA46-01A | 0 | 0 | 0.288415 | 0 | 0 |  | 0 | 0.711585 | 0.337 | 0.101207 | 1.053497 |
| TCGA-DD-A4ND-01A | 0 | 0 | 0.378332 | 0 | 0 |  | 0 | 0.621668 | 0.331 | 0.103873 | 1.048885 |
| TCGA-ED-A8O5-01A | 0 | 0 | 0.313925 | 0 | 0.073674 |  | 0 | 0.612401 | 0.331 | 0.10345 | 1.048487 |
| TCGA-UB-A7ME-01A | 0 | 0 | 0.236687 | 0 | 0.024239 |  | 0.012437 | 0.726637 | 0.271 | 0.150811 | 1.035798 |
| TCGA-CC-5264-01A | 0 | 0 | 0.299396 | 0 | 0.101008 |  | 0 | 0.599596 | 0.308 | 0.119873 | 1.041397 |
| TCGA-G3-A7M7-01A | 0 | 0 | 0.303682 | 0 | 0 |  | 0 | 0.696318 | 0.368 | 0.075556 | 1.063803 |
| TCGA-DD-AACS-01A | 0 | 0 | 0.267329 | 0 | 0.013879 |  | 0.011428 | 0.707364 | 0.256 | 0.164241 | 1.028391 |
| TCGA-DD-AAE0-01A | 0.088398 | 0 | 0.397498 | 0 | 0.080676 |  | 0 | 0.433429 | 0.303 | 0.123521 | 1.032324 |
| TCGA-G3-A5SK-01A | 0 | 0 | 0.295849 | 0 | 0 |  | 0.004092 | 0.700059 | 0.441 | 0.038465 | 1.081283 |
| TCGA-BC-A69I-01A | 0 | 0 | 0.262456 | 0 | 0 |  | 0 | 0.737544 | 0.351 | 0.090051 | 1.059812 |
| TCGA-BC-A10R-01A | 0 | 0 | 0.309159 | 0 | 0 |  | 0 | 0.690841 | 0.383 | 0.066565 | 1.067358 |
| TCGA-5C-AAPD-01A | 0.502448 | 0 | 0.329964 | 0 | 0.16193 |  | 0.005658 | 0 | 0.245 | 0.176245 | 1.018741 |
| TCGA-BC-A10S-01A | 0 | 0 | 0.29811 | 0 | 0 |  | 0.012328 | 0.689561 | 0.354 | 0.08787 | 1.062209 |
| TCGA-DD-AAVX-01A | 0 | 0 | 0.287874 | 0 | 0 |  | 0.004545 | 0.707582 | 0.403 | 0.054313 | 1.07511 |
| TCGA-DD-AAVQ-01A | 0.268485 | 0 | 0.212736 | 0 | 0.043392 |  | 0.005144 | 0.470242 | 0.379 | 0.070584 | 1.05351 |
| TCGA-G3-A3CJ-01A | 0 | 0 | 0.220843 | 0 | 0 |  | 0 | 0.779157 | 0.393 | 0.058748 | 1.076465 |
| TCGA-DD-A4NF-01A | 0 | 0 | 0.154228 | 0 | 0 |  | 0 | 0.845772 | 0.412 | 0.049203 | 1.086543 |
| TCGA-BC-A10Q-01A | 0 | 0 | 0.484149 | 0.181175 | 0.270349 |  | 0 | 0.064328 | 0.569 | -0.01369 | 1.134234 |
| TCGA-DD-A1EE-01A | 0 | 0 | 0.215759 | 0 | 0 |  | 0 | 0.784241 | 0.394 | 0.058248 | 1.077083 |
| TCGA-ZP-A9CY-01A | 0 | 0 | 0.350998 | 0 | 0 |  | 0 | 0.649002 | 0.369 | 0.07487 | 1.061934 |
| TCGA-FV-A23B-01A | 0 | 0 | 0.283095 | 0 | 0 |  | 0.006261 | 0.710645 | 0.295 | 0.12975 | 1.042732 |
| TCGA-HP-A5MZ-01A | 0 | 0 | 0.312631 | 0 | 0 |  | 0.007562 | 0.679808 | 0.396 | 0.057614 | 1.073189 |
| TCGA-DD-AADB-01A | 0.15155 | 0 | 0.443038 | 0 | 0.041602 |  | 0 | 0.36381 | 0.549 | -0.00736 | 1.078415 |
| TCGA-CC-A1HT-01A | 0.365539 | 0 | 0.384087 | 0 | 0.231888 |  | 0.018486 | 0 | 0.407 | 0.051321 | 1.06899 |
| TCGA-DD-A11B-01A | 0 | 0 | 0.311356 | 0 | 0 |  | 0 | 0.688644 | 0.275 | 0.14406 | 1.033755 |
| TCGA-ED-A7XP-01A | 0 | 0 | 0.330423 | 0 | 0 |  | 0 | 0.669577 | 0.367 | 0.078233 | 1.061378 |
| TCGA-FV-A3R3-01A | 0 | 0 | 0.291699 | 0 | 0 |  | 0.007937 | 0.700364 | 0.359 | 0.084539 | 1.062746 |
| TCGA-HP-A5N0-01A | 0 | 0 | 0.344798 | 0 | 0.004246 |  | 0 | 0.650956 | 0.314 | 0.111701 | 1.046392 |
| TCGA-DD-A4NI-01A | 0 | 0 | 0.29524 | 0 | 0 |  | 0 | 0.70476 | 0.387 | 0.063364 | 1.069482 |
| TCGA-EP-A2KB-01A | 0 | 0 | 0.298294 | 0 | 0 |  | 0 | 0.701706 | 0.269 | 0.155267 | 1.029278 |
| TCGA-CC-A7IH-01A | 0 | 0 | 0.306858 | 0 | 0 |  | 0 | 0.693142 | 0.372 | 0.073569 | 1.06449 |
| TCGA-ES-A2HS-01A | 0 | 0 | 0.315024 | 0 | 0 |  | 0 | 0.684976 | 0.249 | 0.171708 | 1.021433 |
| TCGA-G3-A3CG-01A | 0 | 0 | 0.30506 | 0 | 0 |  | 0 | 0.69494 | 0.503 | 0.011433 | 1.090842 |
| TCGA-ED-A5KG-01A | 0.472149 | 0 | 0.484631 | 0 | 0.006274 |  | 0.036311 | 0.000636 | 0.227 | 0.193063 | 1.015236 |
| TCGA-CC-A123-01A | 0 | 0 | 0.321267 | 0 | 0 |  | 0.000547 | 0.678185 | 0.275 | 0.143905 | 1.033603 |
| TCGA-DD-AAD1-01A | 0.002674 | 0 | 0.332079 | 0 | 0.034711 |  | 0 | 0.630536 | 0.297 | 0.127078 | 1.038737 |
| TCGA-DD-A73D-01A | 0 | 0 | 0.310095 | 0 | 0 |  | 0 | 0.689905 | 0.3 | 0.126328 | 1.041562 |
| TCGA-DD-AAVW-01A | 0 | 0 | 0.296247 | 0 | 0 |  | 0 | 0.703753 | 0.248 | 0.173472 | 1.021229 |
| TCGA-DD-AAVS-01A | 0 | 0 | 0.304722 | 0 | 0 |  | 0.011299 | 0.683979 | 0.249 | 0.170281 | 1.024823 |
| TCGA-DD-AACN-01A | 0 | 0 | 0.301895 | 0 | 0 |  | 0 | 0.698105 | 0.337 | 0.100794 | 1.053018 |
| TCGA-DD-A3A2-01A | 0 | 0 | 0.211387 | 0 | 0.013242 |  | 0.0044 | 0.770971 | 0.272 | 0.147027 | 1.037504 |
| TCGA-K7-A5RF-01A | 0 | 0 | 0.262054 | 0 | 0 |  | 0.000155 | 0.737792 | 0.355 | 0.086029 | 1.061644 |
| TCGA-DD-A116-01A | 0 | 0 | 0.219101 | 0 | 0 |  | 0.018612 | 0.762287 | 0.35 | 0.091313 | 1.067356 |
| TCGA-K7-A5RG-01A | 0.43495 | 0 | 0.335186 | 0 | 0.088952 |  | 0 | 0.140912 | 0.24 | 0.179695 | 1.011205 |
| TCGA-BC-A10T-01A | 0 | 0 | 0.325357 | 0 | 0 |  | 0 | 0.674643 | 0.382 | 0.067651 | 1.066089 |
| TCGA-DD-AADJ-01A | 0 | 0 | 0.317476 | 0 | 0 |  | 0 | 0.682524 | 0.279 | 0.140387 | 1.035148 |
| TCGA-DD-AACE-01A | 0 | 0 | 0.278882 | 0 | 0 |  | 0.015963 | 0.705154 | 0.369 | 0.075482 | 1.06983 |
| TCGA-FV-A3R2-01A | 0 | 0 | 0.367522 | 0 | 0.002849 |  | 0 | 0.629629 | 0.308 | 0.120126 | 1.042204 |
| TCGA-DD-AADD-01A | 0 | 0 | 0.302353 | 0 | 0 |  | 0.009319 | 0.688328 | 0.284 | 0.138621 | 1.038697 |
| TCGA-DD-AACX-01A | 0 | 0 | 0.269752 | 0 | 0 |  | 0 | 0.730248 | 0.404 | 0.05367 | 1.0752 |
| TCGA-4R-AA8I-01A | 0 | 0 | 0.255158 | 0 | 0 |  | 0 | 0.744842 | 0.312 | 0.114307 | 1.049502 |
| TCGA-FV-A496-01A | 0 | 0 | 0.32627 | 0 | 0.019421 |  | 0 | 0.65431 | 0.263 | 0.157779 | 1.026426 |
| TCGA-CC-A7IF-01A | 0 | 0 | 0.325155 | 0 | 0 |  | 0 | 0.674845 | 0.309 | 0.117316 | 1.044888 |
| TCGA-DD-AADS-01A | 0 | 0 | 0.244596 | 0 | 0 |  | 0.004404 | 0.751 | 0.274 | 0.144467 | 1.037542 |
| TCGA-DD-AAE9-01A | 0 | 0 | 0.055898 | 0 | 0 |  | 0 | 0.944102 | 0.304 | 0.122581 | 1.061809 |
| TCGA-ZS-A9CF-02A | 0 | 0 | 0.267867 | 0 | 0.004732 |  | 0.012834 | 0.714566 | 0.426 | 0.042883 | 1.083643 |
| TCGA-NI-A4U2-01A | 0 | 0 | 0.324688 | 0 | 0.052136 |  | 0 | 0.623175 | 0.293 | 0.130711 | 1.037061 |
| TCGA-DD-AACI-01A | 0 | 0 | 0.35014 | 0 | 0 |  | 0 | 0.64986 | 0.29 | 0.134386 | 1.036722 |
| TCGA-ZP-A9D1-01A | 0 | 0 | 0.317684 | 0 | 0 |  | 0.003152 | 0.679164 | 0.37 | 0.074839 | 1.064277 |
| TCGA-UB-A7MB-01A | 0 | 0 | 0.344816 | 0 | 0.106267 |  | 0 | 0.548917 | 0.369 | 0.075326 | 1.058816 |
| TCGA-2Y-A9HA-01A | 0 | 0 | 0.21805 | 0 | 0 |  | 0.018542 | 0.763408 | 0.34 | 0.098845 | 1.063931 |
| TCGA-DD-A4NR-01A | 0 | 0 | 0.326976 | 0 | 0 |  | 0.011821 | 0.661203 | 0.404 | 0.052986 | 1.075741 |
| TCGA-RG-A7D4-01A | 0 | 0 | 0.442286 | 0 | 0 |  | 0 | 0.557714 | 0.896 | -0.10388 | 1.129029 |
| TCGA-5R-AAAM-01A | 0 | 0 | 0.367333 | 0 | 0 |  | 0 | 0.632667 | 0.338 | 0.100074 | 1.050791 |
| TCGA-ED-A97K-01A | 0.078283 | 0 | 0.502699 | 0 | 0 |  | 0 | 0.419018 | 0.547 | -0.00691 | 1.083179 |
| TCGA-DD-A3A4-01A | 0 | 0 | 0.285561 | 0 | 0 |  | 0.00581 | 0.708629 | 0.511 | 0.008919 | 1.095075 |
| TCGA-BC-A217-01A | 0.088025 | 0 | 0.432564 | 0 | 0.099665 |  | 0 | 0.379746 | 0.351 | 0.090012 | 1.045433 |
| TCGA-DD-A4NQ-01A | 0 | 0 | 0.301115 | 0 | 0 |  | 0 | 0.698885 | 0.372 | 0.073336 | 1.064889 |
| TCGA-G3-A25T-01A | 0 | 0 | 0.326219 | 0 | 0.102512 |  | 0 | 0.571269 | 0.354 | 0.087556 | 1.054285 |
| TCGA-UB-A7MC-01A | 0 | 0 | 0.300079 | 0 | 0 |  | 0.000741 | 0.69918 | 0.363 | 0.082291 | 1.061296 |
| TCGA-MR-A8JO-01A | 0 | 0 | 0.338088 | 0 | 0.025622 |  | 0.001668 | 0.634622 | 0.535 | -0.00128 | 1.093285 |
| TCGA-DD-AADI-01A | 0 | 0 | 0.401499 | 0 | 0 |  | 0 | 0.598501 | 0.248 | 0.17358 | 1.019059 |
| TCGA-WX-AA47-01A | 0 | 0 | 0.2676 | 0 | 0 |  | 0.014626 | 0.717774 | 0.486 | 0.018175 | 1.095337 |
| TCGA-DD-AADA-01A | 0 | 0 | 0.348459 | 0 | 0 |  | 0.002019 | 0.649522 | 0.325 | 0.10711 | 1.048929 |
| TCGA-WQ-AB4B-01A | 0 | 0 | 0.194319 | 0 | 0 |  | 0.012815 | 0.792866 | 0.337 | 0.101429 | 1.062786 |
| TCGA-YA-A8S7-01A | 0 | 0 | 0.415298 | 0 | 0.158549 |  | 0 | 0.426152 | 0.656 | -0.0368 | 1.102856 |
| TCGA-2Y-A9H2-01A | 0 | 0 | 0.464769 | 0 | 0 |  | 0 | 0.535231 | 0.357 | 0.085834 | 1.054775 |
| TCGA-5C-A9VG-01A | 0 | 0 | 0.281986 | 0 | 0 |  | 0.006505 | 0.711509 | 0.471 | 0.025784 | 1.088351 |
| TCGA-BC-A10Y-01A | 0 | 0 | 0.328749 | 0 | 0.050145 |  | 0 | 0.621105 | 0.363 | 0.082149 | 1.057642 |
| TCGA-EP-A2KA-01A | 0.005791 | 0 | 0.30295 | 0 | 0.041135 |  | 0 | 0.650125 | 0.303 | 0.12354 | 1.040723 |
| TCGA-BC-A10X-01A | 0 | 0 | 0.356575 | 0 | 0 |  | 0 | 0.643425 | 0.363 | 0.082607 | 1.05848 |
| TCGA-DD-AADW-01A | 0 | 0 | 0.390752 | 0 | 0.043893 |  | 0 | 0.565355 | 0.27 | 0.15471 | 1.025794 |
| TCGA-DD-AAED-01A | 0 | 0 | 0.214213 | 0 | 0 |  | 0.008988 | 0.776799 | 0.228 | 0.190205 | 1.018828 |
| TCGA-LG-A9QD-01A | 0 | 0 | 0.292462 | 0 | 0 |  | 0.004235 | 0.703303 | 0.413 | 0.048473 | 1.077252 |
| TCGA-DD-AADV-01A | 0 | 0 | 0.336505 | 0 | 0.014764 |  | 0 | 0.648732 | 0.274 | 0.144246 | 1.032215 |
| TCGA-G3-A3CH-01A | 0 | 0 | 0.363425 | 0 | 0 |  | 0 | 0.636575 | 0.319 | 0.110457 | 1.046534 |
| TCGA-DD-AAW0-01A | 0 | 0 | 0.318462 | 0 | 0 |  | 0.002344 | 0.679194 | 0.291 | 0.133762 | 1.038551 |
| TCGA-DD-AAW3-01A | 0 | 0 | 0.351923 | 0 | 0 |  | 0 | 0.648077 | 0.379 | 0.070344 | 1.063791 |
| TCGA-ED-A7PY-01A | 0 | 0 | 0.270093 | 0 | 0 |  | 0.019132 | 0.710775 | 0.249 | 0.170051 | 1.028081 |
| TCGA-ZP-A9D2-01A | 0.005622 | 0 | 0.512697 | 0 | 0.203332 |  | 0 | 0.27835 | 0.368 | 0.075627 | 1.060464 |
| TCGA-G3-A25Y-01A | 0 | 0 | 0.375452 | 0 | 0.001234 |  | 0 | 0.623314 | 0.247 | 0.173892 | 1.019149 |
| TCGA-G3-A3CI-01A | 0 | 0 | 0.402774 | 0 | 0 |  | 0 | 0.597226 | 0.293 | 0.130609 | 1.037184 |
| TCGA-ZP-A9D0-01A | 0 | 0 | 0.319634 | 0 | 0 |  | 0 | 0.680366 | 0.365 | 0.081262 | 1.060581 |
| TCGA-EP-A3RK-01A | 0 | 0 | 0.32742 | 0 | 0 |  | 0.018435 | 0.654145 | 0.659 | -0.03795 | 1.116521 |
| TCGA-CC-A7IJ-01A | 0.072547 | 0 | 0.455669 | 0 | 0.439346 |  | 0 | 0.032438 | 0.171 | 0.262145 | 0.990619 |
| TCGA-MI-A75H-01A | 0 | 0 | 0.157925 | 0 | 0 |  | 0 | 0.842075 | 0.364 | 0.081886 | 1.071244 |
| TCGA-G3-AAV5-01A | 0 | 0 | 0.335598 | 0 | 0 |  | 0 | 0.664402 | 0.39 | 0.061006 | 1.068407 |
| TCGA-ZP-A9D4-01A | 0 | 0 | 0.305506 | 0 | 0 |  | 0 | 0.694494 | 0.386 | 0.063583 | 1.068822 |
| TCGA-DD-A1ED-01A | 0 | 0 | 0.287561 | 0 | 0 |  | 0.00754 | 0.704899 | 0.45 | 0.033129 | 1.085162 |
| TCGA-DD-A39X-01A | 0 | 0 | 0.246052 | 0 | 0 |  | 0 | 0.753948 | 0.302 | 0.124871 | 1.045289 |
| TCGA-ED-A459-01A | 0 | 0 | 0.279407 | 0 | 0.00469 |  | 0.007402 | 0.708501 | 0.418 | 0.046589 | 1.079557 |
| TCGA-2Y-A9GX-01A | 0 | 0 | 0.361264 | 0 | 0 |  | 0 | 0.638736 | 0.447 | 0.036792 | 1.077304 |
| TCGA-DD-A73E-01A | 0 | 0 | 0.266837 | 0 | 0 |  | 0 | 0.733163 | 0.409 | 0.050158 | 1.076907 |
| TCGA-DD-AACL-01A | 0.066502 | 0 | 0.499355 | 0 | 0.008191 |  | 0.02659 | 0.399361 | 0.855 | -0.09037 | 1.124814 |
| TCGA-MR-A520-01A | 0 | 0 | 0.352594 | 0 | 0 |  | 0 | 0.647406 | 0.291 | 0.132779 | 1.037343 |
| TCGA-DD-A1EF-01A | 0 | 0 | 0.296334 | 0 | 0 |  | 0 | 0.703666 | 0.329 | 0.105185 | 1.051375 |
| TCGA-FV-A4ZP-01A | 0 | 0 | 0.37035 | 0 | 0.087156 |  | 0 | 0.542494 | 0.251 | 0.166502 | 1.020166 |
| TCGA-WX-AA44-01A | 0 | 0 | 0.286348 | 0 | 0.055852 |  | 0 | 0.657799 | 0.285 | 0.137894 | 1.035102 |
| TCGA-ED-A7PX-01A | 0.19066 | 0 | 0.282064 | 0 | 0.440283 |  | 0 | 0.086993 | 0.49 | 0.01702 | 1.096762 |
| TCGA-CC-A7IG-01A | 0 | 0 | 0.368122 | 0 | 0.13927 |  | 0 | 0.492608 | 0.296 | 0.128646 | 1.036051 |
| TCGA-G3-A25X-01A | 0.045587 | 0 | 0.549465 | 0 | 0.092228 |  | 0 | 0.31272 | 0.529 | 0.00107 | 1.082821 |
| TCGA-G3-A25U-01A | 0 | 0 | 0.387288 | 0 | 0.010735 |  | 0 | 0.601977 | 0.451 | 0.032706 | 1.077405 |
| TCGA-BC-4072-01B | 0.121159 | 0 | 0.537289 | 0 | 0.09535 |  | 0 | 0.246201 | 0.456 | 0.031085 | 1.067277 |
| TCGA-XR-A8TC-01A | 0 | 0 | 0.294937 | 0 | 0 |  | 0.012298 | 0.692764 | 0.296 | 0.128322 | 1.044376 |
| TCGA-BC-A3KF-01A | 0 | 0 | 0.256591 | 0 | 0 |  | 0 | 0.743409 | 0.27 | 0.153402 | 1.031868 |
| TCGA-O8-A75V-01A | 0 | 0 | 0.274407 | 0 | 0 |  | 0.018285 | 0.707308 | 0.339 | 0.099373 | 1.060087 |
| TCGA-BC-A8YO-01A | 0.029233 | 0 | 0.374357 | 0 | 0.128287 |  | 0 | 0.468123 | 0.367 | 0.078436 | 1.054344 |
| TCGA-DD-A39V-01A | 0 | 0 | 0.207615 | 0 | 0 |  | 0 | 0.792385 | 0.362 | 0.083195 | 1.066567 |
| TCGA-DD-A1EC-01A | 0.257147 | 0 | 0.502738 | 0 | 0 |  | 0.039626 | 0.200489 | 0.744 | -0.06351 | 1.10783 |
| TCGA-DD-AACQ-01A | 0 | 0 | 0.273835 | 0 | 0.01175 |  | 0 | 0.714414 | 0.25 | 0.167077 | 1.024359 |
| TCGA-DD-A1EH-01A | 0 | 0 | 0.305059 | 0 | 0 |  | 0 | 0.694941 | 0.245 | 0.176501 | 1.019594 |
| TCGA-KR-A7K7-01A | 0 | 0 | 0.283466 | 0 | 0 |  | 0 | 0.716534 | 0.403 | 0.054109 | 1.074142 |
| TCGA-DD-AAVV-01A | 0 | 0 | 0.393519 | 0 | 0 |  | 0 | 0.606481 | 0.384 | 0.064873 | 1.064595 |
| TCGA-DD-AADN-01A | 0.673967 | 0 | 0.27673 | 0 | 0 |  | 0.049303 | 0 | 0.246 | 0.175938 | 1.033489 |
| TCGA-LG-A9QC-01A | 0 | 0 | 0.265873 | 0 | 0 |  | 0.019117 | 0.715009 | 0.401 | 0.055403 | 1.080575 |
| TCGA-RC-A7S9-01A | 0 | 0 | 0.334546 | 0 | 0.015478 |  | 0.003363 | 0.646614 | 0.309 | 0.116867 | 1.044863 |
| TCGA-BC-A112-01A | 0 | 0 | 0.520093 | 0 | 0.027309 |  | 0 | 0.452598 | 0.283 | 0.139237 | 1.033048 |
| TCGA-2Y-A9H0-01A | 0 | 0 | 0.261859 | 0 | 0.076287 |  | 0 | 0.661854 | 0.341 | 0.098682 | 1.052643 |
| TCGA-DD-AAW2-01A | 0 | 0 | 0.199785 | 0 | 0 |  | 0 | 0.800215 | 0.33 | 0.104379 | 1.057557 |
| TCGA-KR-A7K2-01A | 0 | 0 | 0.297965 | 0 | 0 |  | 0.002554 | 0.699482 | 0.248 | 0.172714 | 1.022039 |
| TCGA-DD-AADU-01A | 0 | 0 | 0.221768 | 0 | 0 |  | 0 | 0.778232 | 0.368 | 0.077786 | 1.067939 |
| TCGA-ED-A66Y-01A | 0 | 0 | 0.282629 | 0 | 0.2791 |  | 0 | 0.438271 | 0.338 | 0.09965 | 1.053741 |
| TCGA-CC-A8HT-01A | 0 | 0 | 0.408655 | 0 | 0.040849 |  | 0 | 0.550495 | 0.328 | 0.105919 | 1.045981 |
| TCGA-DD-AADY-01A | 0 | 0 | 0.393123 | 0 | 0.035439 |  | 0 | 0.571439 | 0.35 | 0.092004 | 1.052123 |
| TCGA-BC-A216-01A | 0 | 0 | 0.373918 | 0 | 0.021397 |  | 0 | 0.604686 | 0.337 | 0.1006 | 1.049487 |
| TCGA-G3-AAUZ-01A | 0 | 0 | 0.207556 | 0 | 0 |  | 0.013196 | 0.779248 | 0.379 | 0.071176 | 1.075799 |
| TCGA-RC-A7SK-01A | 0 | 0 | 0.300157 | 0 | 0 |  | 0 | 0.699843 | 0.288 | 0.135885 | 1.037774 |
| TCGA-G3-A25Z-01A | 0 | 0 | 0.289269 | 0 | 0 |  | 0.013826 | 0.696905 | 0.324 | 0.107491 | 1.054379 |
| TCGA-DD-AAD8-01A | 0 | 0 | 0.265947 | 0 | 0 |  | 0.016951 | 0.717102 | 0.325 | 0.107138 | 1.056654 |
| TCGA-DD-AAEE-01A | 0 | 0 | 0.220793 | 0 | 0 |  | 0 | 0.779207 | 0.274 | 0.144378 | 1.037883 |
| TCGA-2Y-A9GU-01A | 0 | 0 | 0.315058 | 0 | 0.026414 |  | 0 | 0.658527 | 0.305 | 0.121397 | 1.042301 |
| TCGA-DD-A119-01A | 0 | 0 | 0.296121 | 0 | 0 |  | 0 | 0.703879 | 0.347 | 0.094729 | 1.055926 |
| TCGA-G3-AAV6-01A | 0 | 0 | 0.290902 | 0 | 0.032284 |  | 0 | 0.676814 | 0.312 | 0.114178 | 1.046151 |
| TCGA-DD-A73F-01A | 0 | 0 | 0.31769 | 0 | 0 |  | 0 | 0.68231 | 0.275 | 0.143726 | 1.03368 |
| TCGA-CC-A5UD-01A | 0 | 0 | 0.219755 | 0 | 0.187135 |  | 0 | 0.59311 | 0.444 | 0.037901 | 1.079972 |
| TCGA-DD-AACD-01A | 0 | 0 | 0.309713 | 0 | 0 |  | 0 | 0.690287 | 0.404 | 0.052844 | 1.073155 |
| TCGA-CC-A8HS-01A | 0 | 0 | 0.3968 | 0 | 0.163945 |  | 0 | 0.439255 | 0.309 | 0.117068 | 1.041047 |
| TCGA-DD-A11D-01A | 0 | 0 | 0.30012 | 0 | 0 |  | 0.011103 | 0.688777 | 0.277 | 0.141702 | 1.037849 |
| TCGA-DD-A1EG-01A | 0 | 0 | 0.380512 | 0 | 0 |  | 0 | 0.619488 | 0.416 | 0.047763 | 1.07203 |
| TCGA-PD-A5DF-01A | 0 | 0 | 0.330741 | 0 | 0.007295 |  | 0 | 0.661964 | 0.372 | 0.072877 | 1.06325 |
| TCGA-DD-A3A9-01A | 0 | 0 | 0.357844 | 0 | 0 |  | 0.002769 | 0.639387 | 0.247 | 0.174623 | 1.019708 |
| TCGA-2Y-A9H4-01A | 0 | 0 | 0.31461 | 0 | 0 |  | 0 | 0.68539 | 0.322 | 0.108358 | 1.049177 |
| TCGA-DD-AACK-01A | 0 | 0 | 0.320854 | 0 | 0 |  | 0 | 0.679146 | 0.351 | 0.090206 | 1.056707 |
| TCGA-DD-A118-01A | 0 | 0 | 0.37922 | 0 | 0.072303 |  | 0 | 0.548477 | 0.35 | 0.091122 | 1.051891 |
| TCGA-DD-A4NL-01A | 0 | 0 | 0.324647 | 0 | 0 |  | 0 | 0.675353 | 0.38 | 0.068738 | 1.065663 |
| TCGA-5C-A9VH-01A | 0 | 0 | 0.299367 | 0 | 0 |  | 0 | 0.700633 | 0.241 | 0.179443 | 1.018452 |
| TCGA-3K-AAZ8-01A | 0 | 0 | 0.354099 | 0 | 0 |  | 0 | 0.645901 | 0.309 | 0.116279 | 1.044344 |
| TCGA-MI-A75G-01A | 0 | 0 | 0.244486 | 0 | 0 |  | 0 | 0.755514 | 0.271 | 0.150828 | 1.033634 |
| TCGA-DD-A3A6-01A | 0.296199 | 0 | 0.53503 | 0.147562 | 0 |  | 0.021209 | 0 | 0.132 | 0.32692 | 0.960656 |
| TCGA-DD-AADM-01A | 0 | 0 | 0.202191 | 0 | 0 |  | 0.012858 | 0.78495 | 0.368 | 0.077405 | 1.073271 |
| TCGA-DD-AAC8-01A | 0 | 0 | 0.266292 | 0 | 0 |  | 0 | 0.733708 | 0.484 | 0.019307 | 1.090179 |
| TCGA-DD-A4NN-01A | 0 | 0 | 0.300911 | 0 | 0 |  | 0 | 0.699089 | 0.228 | 0.191877 | 1.012816 |
| TCGA-DD-A4NH-01A | 0 | 0 | 0.46879 | 0 | 0 |  | 0 | 0.53121 | 0.271 | 0.149595 | 1.028974 |
| TCGA-K7-A6G5-01A | 0 | 0 | 0.313183 | 0 | 0 |  | 0.009935 | 0.676882 | 0.362 | 0.083032 | 1.062885 |
| TCGA-DD-AACU-01A | 0 | 0 | 0.207212 | 0 | 0.003009 |  | 0.009497 | 0.780281 | 0.242 | 0.178317 | 1.024807 |
| TCGA-CC-A7IL-01A | 0 | 0 | 0.198857 | 0 | 0 |  | 0 | 0.801143 | 0.281 | 0.139366 | 1.041554 |
| TCGA-BW-A5NO-01A | 0.031816 | 0 | 0.276174 | 0 | 0.072504 |  | 0 | 0.619505 | 0.422 | 0.044101 | 1.072063 |
| TCGA-XR-A8TG-01A | 0 | 0 | 0.260804 | 0 | 0.017913 |  | 0.017499 | 0.703784 | 0.423 | 0.043356 | 1.084718 |
| TCGA-DD-A1EJ-01A | 0 | 0 | 0.308739 | 0 | 0 |  | 0.010543 | 0.680717 | 0.253 | 0.165234 | 1.026804 |
| TCGA-G3-A7M8-01A | 0 | 0 | 0.341475 | 0 | 0 |  | 0 | 0.658525 | 0.297 | 0.127131 | 1.040096 |
| TCGA-UB-AA0V-01A | 0 | 0 | 0.349073 | 0 | 0 |  | 0 | 0.650927 | 0.354 | 0.088689 | 1.056199 |
| TCGA-DD-AACZ-01A | 0 | 0 | 0.389952 | 0 | 0.067165 |  | 0 | 0.542883 | 0.449 | 0.034419 | 1.074828 |
| TCGA-DD-A1EB-01A | 0 | 0 | 0.391218 | 0 | 0 |  | 0 | 0.608782 | 0.313 | 0.11386 | 1.044399 |
| TCGA-BC-4073-01B | 0.151871 | 0 | 0.539461 | 0 | 0.018049 |  | 0 | 0.290619 | 0.545 | -0.00458 | 1.077896 |
| TCGA-ZS-A9CG-01A | 0 | 0 | 0.335792 | 0 | 0 |  | 0 | 0.664208 | 0.421 | 0.04494 | 1.075119 |
| TCGA-BD-A2L6-01A | 0 | 0 | 0.184007 | 0 | 0.078214 |  | 0.005656 | 0.732123 | 0.26 | 0.161847 | 1.0297 |
| TCGA-CC-5260-01A | 0.324812 | 0 | 0.355885 | 0 | 0.319303 |  | 0 | 0 | 0.328 | 0.106005 | 1.047744 |
| TCGA-DD-AAD5-01A | 0 | 0 | 0.552132 | 0 | 0.081178 |  | 0 | 0.36669 | 0.533 | -0.00101 | 1.087465 |
| TCGA-BD-A3EP-01A | 0.120701 | 0 | 0.383408 | 0 | 0.004961 |  | 0 | 0.490929 | 0.582 | -0.01797 | 1.086413 |
| TCGA-WQ-A9G7-01A | 0 | 0 | 0.35017 | 0 | 0.04571 |  | 0 | 0.60412 | 0.35 | 0.09128 | 1.053218 |
| TCGA-FV-A2QR-01A | 0 | 0 | 0.405201 | 0 | 0.046474 |  | 0 | 0.548326 | 0.337 | 0.101241 | 1.047811 |
| TCGA-DD-AAC9-01A | 0 | 0 | 0.315721 | 0 | 0 |  | 0 | 0.684279 | 0.392 | 0.059494 | 1.070019 |
| TCGA-DD-AA3A-01A | 0.084547 | 0.126155 | 0.161902 | 0 | 0 |  | 0 | 0.627396 | 0.227 | 0.194368 | 1.013133 |
| TCGA-CC-5263-01A | 0 | 0 | 0.364239 | 0 | 0.358074 |  | 0 | 0.277687 | 0.427 | 0.042774 | 1.08416 |
| TCGA-FV-A495-01A | 0 | 0 | 0.278885 | 0 | 0 |  | 0.003629 | 0.717486 | 0.276 | 0.14195 | 1.036827 |
| TCGA-BC-A10Z-01A | 0 | 0 | 0.337681 | 0 | 0.002842 |  | 0 | 0.659477 | 0.366 | 0.080649 | 1.059898 |
| TCGA-DD-AAVR-01A | 0 | 0 | 0.282302 | 0 | 0 |  | 0.012752 | 0.704947 | 0.343 | 0.096605 | 1.05929 |
| TCGA-ZS-A9CE-01A | 0 | 0 | 0.202214 | 0 | 0 |  | 0.009093 | 0.788693 | 0.334 | 0.102733 | 1.060553 |
| TCGA-CC-5259-01A | 0 | 0 | 0.313416 | 0 | 0 |  | 0.008517 | 0.678067 | 0.287 | 0.136191 | 1.039154 |
| TCGA-5R-AA1D-01A | 0 | 0 | 0.252528 | 0 | 0 |  | 0.022994 | 0.724478 | 0.518 | 0.005179 | 1.10516 |
| TCGA-ED-A82E-01A | 0.328218 | 0.080623 | 0 | 0 | 0.414539 |  | 0 | 0.17662 | 0.238 | 0.182544 | 1.026982 |
| TCGA-DD-A3A1-01A | 0 | 0 | 0.295516 | 0 | 0 |  | 0.005996 | 0.698489 | 0.35 | 0.091363 | 1.05902 |
| TCGA-DD-AAEA-01A | 0 | 0 | 0.270239 | 0 | 0 |  | 0 | 0.729761 | 0.252 | 0.165541 | 1.025768 |
| TCGA-ZS-A9CF-01A | 0 | 0 | 0.265974 | 0 | 0 |  | 0.013236 | 0.72079 | 0.354 | 0.088727 | 1.063868 |
| TCGA-G3-A25V-01A | 0 | 0 | 0.328165 | 0 | 0 |  | 0 | 0.671835 | 0.367 | 0.07927 | 1.061039 |
| TCGA-CC-A9FS-01A | 0 | 0 | 0.198761 | 0 | 0.000541 |  | 0.008762 | 0.791937 | 0.249 | 0.17114 | 1.028664 |
| TCGA-BC-A69H-01A | 0 | 0 | 0.368365 | 0 | 0.020601 |  | 0 | 0.611034 | 0.249 | 0.170759 | 1.019894 |
| TCGA-DD-A3A7-01A | 0 | 0 | 0.269815 | 0 | 0 |  | 0 | 0.730185 | 0.246 | 0.17593 | 1.021076 |
| TCGA-G3-A7M6-01A | 0 | 0 | 0.454695 | 0 | 0.072386 |  | 0 | 0.47292 | 0.333 | 0.102906 | 1.046371 |
| TCGA-MI-A75E-01A | 0 | 0 | 0.226947 | 0 | 0 |  | 0 | 0.773053 | 0.363 | 0.082667 | 1.065392 |
| TCGA-DD-AAE4-01A | 0.00545 | 0 | 0.334247 | 0 | 0 |  | 0 | 0.660303 | 0.483 | 0.020113 | 1.084866 |
| TCGA-CC-A7II-01A | 0 | 0 | 0.422982 | 0 | 0.478342 |  | 0 | 0.098675 | 0.329 | 0.105516 | 1.072036 |
| TCGA-G3-A7M5-01A | 0 | 0 | 0.289973 | 0 | 0 |  | 0.000637 | 0.70939 | 0.265 | 0.156756 | 1.029065 |
| TCGA-CC-A3MC-01A | 0 | 0 | 0.281696 | 0 | 0 |  | 0.013264 | 0.70504 | 0.303 | 0.122987 | 1.047642 |
| TCGA-ES-A2HT-01A | 0 | 0 | 0.275661 | 0 | 0 |  | 0.002741 | 0.721598 | 0.384 | 0.064494 | 1.070925 |
| TCGA-G3-A6UC-01A | 0 | 0 | 0.274531 | 0 | 0 |  | 0 | 0.725469 | 0.349 | 0.09267 | 1.057966 |
| TCGA-BD-A3ER-01A | 0 | 0 | 0.295449 | 0 | 0 |  | 0.014002 | 0.690549 | 0.387 | 0.063218 | 1.073676 |
| TCGA-DD-A3A5-01A | 0 | 0 | 0.310639 | 0 | 0 |  | 0.006159 | 0.683202 | 0.272 | 0.147415 | 1.033706 |
| TCGA-DD-AACH-01A | 0 | 0 | 0.300543 | 0 | 0.051857 |  | 0 | 0.647599 | 0.3 | 0.126169 | 1.039824 |
| TCGA-CC-5262-01A | 0 | 0 | 0.285608 | 0 | 0.00279 |  | 0 | 0.711602 | 0.38 | 0.06881 | 1.067529 |
| TCGA-DD-AADG-01A | 0 | 0 | 0.241602 | 0 | 0 |  | 0 | 0.758398 | 0.342 | 0.097271 | 1.057904 |
| TCGA-DD-A39Z-01A | 0 | 0 | 0.299134 | 0 | 0 |  | 0.004614 | 0.696252 | 0.279 | 0.1403 | 1.036939 |
| TCGA-DD-AACJ-01A | 0 | 0 | 0.280133 | 0 | 0 |  | 0 | 0.719867 | 0.313 | 0.113102 | 1.048704 |
| TCGA-DD-AADL-01A | 0 | 0 | 0.350173 | 0 | 0 |  | 0 | 0.649827 | 0.351 | 0.091036 | 1.055169 |
| TCGA-DD-AAEG-01A | 0 | 0 | 0.316111 | 0 | 0 |  | 0 | 0.683889 | 0.368 | 0.076796 | 1.062651 |
| TCGA-DD-A4NO-01A | 0 | 0 | 0.19961 | 0 | 0 |  | 0.011419 | 0.788971 | 0.278 | 0.140628 | 1.043717 |
| TCGA-CC-A5UE-01A | 0 | 0 | 0.286763 | 0 | 0.02872 |  | 0 | 0.684517 | 0.318 | 0.110829 | 1.047946 |
| TCGA-G3-A7M9-01A | 0 | 0 | 0.402457 | 0 | 0.160404 |  | 0 | 0.437139 | 0.314 | 0.111664 | 1.043223 |
| TCGA-2Y-A9HB-01A | 0 | 0 | 0.295343 | 0 | 0 |  | 0.011911 | 0.692746 | 0.379 | 0.071216 | 1.069542 |
| TCGA-DD-A39W-01A | 0 | 0 | 0.33158 | 0 | 0.02034 |  | 0 | 0.64808 | 0.477 | 0.024107 | 1.082912 |
| TCGA-2Y-A9GV-01A | 0 | 0 | 0.319003 | 0 | 0 |  | 0.007474 | 0.673523 | 0.339 | 0.099296 | 1.054859 |
| TCGA-DD-AACF-01A | 0 | 0 | 0.357371 | 0 | 0 |  | 0 | 0.642629 | 0.347 | 0.094368 | 1.053511 |
| TCGA-UB-A7MA-01A | 0 | 0 | 0.39005 | 0 | 0.052526 |  | 0 | 0.557424 | 0.287 | 0.136425 | 1.033284 |
| TCGA-DD-AAE7-01A | 0 | 0 | 0.344983 | 0 | 0 |  | 0 | 0.655017 | 0.322 | 0.108961 | 1.047755 |
| TCGA-MI-A75C-01A | 0 | 0 | 0.303574 | 0 | 0 |  | 0.010619 | 0.685807 | 0.383 | 0.066508 | 1.070756 |
| TCGA-DD-A4NA-01A | 0.381034 | 0 | 0.519171 | 0 | 0.084363 |  | 0.015431 | 0 | 0.548 | -0.00701 | 1.085631 |
| TCGA-ZP-A9CV-01A | 0 | 0 | 0.330871 | 0 | 0 |  | 0 | 0.669129 | 0.358 | 0.084944 | 1.058513 |
| TCGA-RC-A6M6-01A | 0 | 0 | 0.338782 | 0 | 0 |  | 0.002965 | 0.658253 | 0.33 | 0.10454 | 1.050612 |
| TCGA-T1-A6J8-01A | 0 | 0 | 0.192727 | 0 | 0 |  | 0.009808 | 0.797466 | 0.244 | 0.177067 | 1.026464 |
| TCGA-DD-A1EK-01A | 0 | 0 | 0.296248 | 0 | 0 |  | 0.013212 | 0.69054 | 0.301 | 0.125877 | 1.045655 |
| TCGA-KR-A7K8-01A | 0.140203 | 0 | 0.276558 | 0 | 0.034574 |  | 0 | 0.548665 | 0.474 | 0.025125 | 1.072215 |
| TCGA-DD-AAD6-01A | 0 | 0 | 0.299884 | 0 | 0 |  | 0.011786 | 0.68833 | 0.393 | 0.05879 | 1.074668 |
| TCGA-DD-A4NB-01A | 0 | 0 | 0.27546 | 0 | 0 |  | 0.010875 | 0.713665 | 0.484 | 0.019305 | 1.093015 |
| TCGA-DD-A11C-01A | 0 | 0 | 0.311832 | 0 | 0.003842 |  | 0 | 0.684326 | 0.401 | 0.055307 | 1.071779 |
| TCGA-RC-A7SF-01A | 0 | 0 | 0.308327 | 0 | 0 |  | 0.010565 | 0.681108 | 0.313 | 0.113248 | 1.050041 |
| TCGA-ED-A8O6-01A | 0 | 0 | 0.280132 | 0 | 0 |  | 0.017268 | 0.7026 | 0.307 | 0.120457 | 1.049956 |
| TCGA-DD-AAEB-01A | 0 | 0 | 0.11532 | 0 | 0 |  | 0 | 0.88468 | 0.309 | 0.117831 | 1.05824 |
| TCGA-DD-AAVP-01A | 0 | 0 | 0.318837 | 0 | 0 |  | 0 | 0.681163 | 0.474 | 0.025107 | 1.084297 |
| TCGA-DD-A73A-01A | 0 | 0 | 0.326362 | 0 | 0 |  | 0 | 0.673638 | 0.349 | 0.093326 | 1.055136 |
| TCGA-RC-A6M4-01A | 0 | 0 | 0.182199 | 0 | 0 |  | 0 | 0.817801 | 0.303 | 0.123373 | 1.050115 |
| TCGA-G3-AAV0-01A | 0 | 0 | 0.342943 | 0 | 0 |  | 0.002121 | 0.654936 | 0.271 | 0.152155 | 1.029706 |
| TCGA-DD-AAVY-01A | 0 | 0 | 0.361352 | 0 | 0 |  | 0 | 0.638648 | 0.465 | 0.027654 | 1.081049 |
| TCGA-DD-A1EL-01A | 0 | 0 | 0.12403 | 0 | 0 |  | 0 | 0.87597 | 0.379 | 0.070997 | 1.079485 |
| TCGA-DD-A4NJ-01A | 0 | 0 | 0.323139 | 0 | 0.017295 |  | 0 | 0.659566 | 0.398 | 0.056667 | 1.069927 |
| TCGA-G3-A5SM-01A | 0 | 0 | 0.282591 | 0 | 0 |  | 0 | 0.717409 | 0.324 | 0.107495 | 1.051043 |
| TCGA-G3-A5SL-01A | 0 | 0 | 0.171506 | 0 | 0 |  | 0 | 0.828494 | 0.35 | 0.092252 | 1.06531 |
| TCGA-BC-A10U-01A | 0 | 0 | 0.285818 | 0 | 0 |  | 0.014952 | 0.69923 | 0.398 | 0.056626 | 1.077428 |
| TCGA-DD-AACG-01A | 0 | 0 | 0.317114 | 0 | 0 |  | 0.007781 | 0.675106 | 0.284 | 0.138633 | 1.037756 |
| TCGA-DD-A3A3-01A | 0 | 0 | 0.302737 | 0 | 0 |  | 0 | 0.697263 | 0.36 | 0.083786 | 1.060318 |
| TCGA-ED-A7XO-01A | 0 | 0 | 0.382647 | 0 | 0 |  | 0 | 0.617353 | 0.342 | 0.097464 | 1.051446 |
| TCGA-DD-AACB-01A | 0 | 0 | 0.46622 | 0 | 0 |  | 0 | 0.53378 | 0.886 | -0.09909 | 1.126256 |
| TCGA-RC-A7SH-01A | 0 | 0 | 0.307542 | 0 | 0 |  | 0 | 0.692458 | 0.234 | 0.1861 | 1.015228 |
| TCGA-CC-5261-01A | 0.143296 | 0 | 0.283807 | 0 | 0.08942 |  | 0 | 0.483477 | 0.321 | 0.109112 | 1.037017 |
| TCGA-DD-A3A8-01A | 0 | 0 | 0.266268 | 0 | 0 |  | 0 | 0.733732 | 0.367 | 0.080011 | 1.063982 |
| TCGA-DD-A73C-01A | 0 | 0 | 0.250822 | 0 | 0.004105 |  | 0.002292 | 0.742781 | 0.272 | 0.14686 | 1.035415 |
| TCGA-GJ-A9DB-01A | 0 | 0 | 0.332444 | 0 | 0.005189 |  | 0 | 0.662367 | 0.28 | 0.139561 | 1.034773 |
| TCGA-DD-AAW1-01A | 0 | 0 | 0.237079 | 0 | 0 |  | 0 | 0.762921 | 0.404 | 0.053662 | 1.077478 |
| TCGA-XR-A8TF-01A | 0 | 0 | 0.259224 | 0 | 0 |  | 0.008011 | 0.732766 | 0.363 | 0.082229 | 1.065664 |
| TCGA-CC-A5UC-01A | 0 | 0 | 0.353492 | 0 | 0 |  | 0 | 0.646508 | 0.35 | 0.091707 | 1.054766 |
| TCGA-DD-A39Y-01A | 0 | 0 | 0.276861 | 0 | 0.083504 |  | 0.004074 | 0.635561 | 0.521 | 0.004135 | 1.093236 |
| TCGA-DD-A114-01A | 0.282654 | 0 | 0.384578 | 0 | 0.332768 |  | 0 | 0 | 0.353 | 0.088885 | 1.055684 |
| TCGA-NI-A8LF-01A | 0 | 0 | 0.172214 | 0 | 0 |  | 0.01537 | 0.812416 | 0.361 | 0.083271 | 1.073762 |
| TCGA-DD-AADF-01A | 0.122956 | 0 | 0.338738 | 0 | 0.020613 |  | 0 | 0.517693 | 0.681 | -0.04528 | 1.097805 |
| TCGA-DD-AAEI-01A | 0 | 0 | 0.282092 | 0 | 0 |  | 0.015619 | 0.702289 | 0.306 | 0.120739 | 1.049276 |
| TCGA-2Y-A9H5-01A | 0 | 0 | 0.353339 | 0 | 0 |  | 0 | 0.646661 | 0.297 | 0.127479 | 1.039591 |

**Table S2.** Analysis of prognosis and clinical correlation

| Ssample | censor | patient | os | age | gender | type | grade | stage | weight | fibrosis | TP53 | naive/memory CD8 T cells | exhausted CD8 T cells 1 | exhausted CD8 T cells 2 | cytotoxic CD8 T cells 1 | cytotoxic CD8 T cells 2 | cytotoxic CD8 T cells 3 | cytotoxic CD8 T cells 4 | P value | Correlation | RMSE | cluster | cytotoxic4 Type | exhausted2 Type | Naïve Type |
| --- | --- | --- | --- | --- | --- | --- | --- | --- | --- | --- | --- | --- | --- | --- | --- | --- | --- | --- | --- | --- | --- | --- | --- | --- | --- |
| TCGA-DD-A4NG-01A | 1 | TCGA-DD-A4NG | 802 | 77 | male | Primary Tumor | G2 | stage iiia | 94 |  | WT | 0 | 0 | 0.291202 | 0 | 0 | 0 | 0.708798 | 0.378 | 0.071275 | 1.066314 | cluster 1 | High | Low | Low |
| TCGA-G3-AAV4-01A | 1 | TCGA-G3-AAV4 | 27 | 83 | female | Primary Tumor | G1 | stage i | 56 | 3,4 - Fibrous Speta | Mut | 0 | 0 | 0.025874 | 0 | 0 | 0.000895 | 0.973231 | 0.366 | 0.081085 | 1.085854 | cluster 1 | High | Low | Low |
| TCGA-2Y-A9H1-01A | 1 | TCGA-2Y-A9H1 | 1229 | 58 | male | Primary Tumor | G2 | stage i | 89 | 1,2 - Portal Fibrosis | WT | 0 | 0 | 0.298497 | 0 | 0 | 0 | 0.701503 | 0.342 | 0.097389 | 1.054657 | cluster 1 | High | Low | Low |
| TCGA-CC-A3M9-01A | 1 | TCGA-CC-A3M9 | 300 | 45 | male | Primary Tumor | G3 | stage iiia | 61 |  | Mut | 0.285245 | 0 | 0.339022 | 0.052287 | 0.308209 | 0.015237 | 0 | 0.015 | 0.690601 | 0.754421 | cluster 2 | Low | High | High |
| TCGA-K7-AAU7-01A | 0 | TCGA-K7-AAU7 | 359 | 61 | male | Primary Tumor | G2 | stage ii | NA |  | Mut | 0 | 0 | 0.308202 | 0 | 0.047132 | 0.003005 | 0.641661 | 0.411 | 0.049703 | 1.073196 | cluster 2 | High | Low | Low |
| TCGA-BC-A10W-01A | 1 | TCGA-BC-A10W | 91 | 50 | male | Primary Tumor | G3 | not reported | 71 |  | WT | 0 | 0 | 0.362017 | 0 | 0.152848 | 0 | 0.485135 | 0.505 | 0.01087 | 1.084913 | cluster 2 | Low | High | Low |
| TCGA-DD-AACV-01A | 0 | TCGA-DD-AACV | 1531 | 53 | male | Primary Tumor | G3 | stage i | 70 |  | Mut | 0 | 0 | 0.292337 | 0 | 0.034779 | 0 | 0.672883 | 0.35 | 0.092291 | 1.05545 | cluster 2 | High | Low | Low |
| TCGA-DD-AAD3-01A | 0 | TCGA-DD-AAD3 | 1295 | 43 | male | Primary Tumor | G2 | stage i | 77 | 1,2 - Portal Fibrosis | Mut | 0 | 0 | 0.343982 | 0 | 0 | 0.002931 | 0.653087 | 0.288 | 0.13481 | 1.037393 | cluster 1 | High | High | Low |
| TCGA-DD-AACA-01A | 0 | TCGA-DD-AACA | 2301 | 65 | male | Primary Tumor | G3 | stage i | 70 | 6 - Established Cirrhosis | WT | 0 | 0 | 0.104664 | 0 | 0 | 0.009218 | 0.886118 | 0.377 | 0.071661 | 1.083793 | cluster 1 | High | Low | Low |
| TCGA-G3-AAV2-01A | 0 | TCGA-G3-AAV2 | 372 | 50 | male | Primary Tumor | G1 | stage i | NA | 6 - Established Cirrhosis | WT | 0 | 0 | 0.293725 | 0 | 0 | 0 | 0.706275 | 0.405 | 0.052234 | 1.074324 | cluster 1 | High | Low | Low |
| TCGA-DD-AACT-01A | 0 | TCGA-DD-AACT | 1562 | 69 | female | Primary Tumor | G2 | stage i | 62 |  | WT | 0 | 0 | 0.35542 | 0 | 0 | 0 | 0.64458 | 0.394 | 0.05817 | 1.068722 | cluster 1 | High | High | Low |
| TCGA-GJ-A6C0-01A | 1 | TCGA-GJ-A6C0 | 31 | 75 | female | Primary Tumor | G2 | stage ii | 90 |  | WT | 0 | 0 | 0.290391 | 0 | 0.018195 | 0.005852 | 0.685562 | 0.291 | 0.13275 | 1.040094 | cluster 2 | High | Low | Low |
| TCGA-CC-5258-01A | 1 | TCGA-CC-5258 | 129 | 48 | male | Primary Tumor | G2 | stage ii | 55 |  | Mut | 0 | 0 | 0.311317 | 0 | 0.05142 | 0 | 0.637263 | 0.368 | 0.077733 | 1.060165 | cluster 2 | High | Low | Low |
| TCGA-QA-A7B7-01A | 0 | TCGA-QA-A7B7 | 94 | 48 | male | Primary Tumor | G2 | stage ii | 108 | 0 - No Fibrosis | Mut | 0 | 0 | 0.410457 | 0 | 0.120389 | 0 | 0.469154 | 0.449 | 0.034949 | 1.073874 | cluster 2 | Low | High | Low |
| TCGA-2Y-A9H3-01A | 0 | TCGA-2Y-A9H3 | 1516 | 45 | male | Primary Tumor | G1 | stage ii | 105 | 1,2 - Portal Fibrosis | WT | 0.437199 | 0 | 0.462452 | 0.016014 | 0.006116 | 0 | 0.078219 | 0.271 | 0.150154 | 1.026613 | cluster 1 | Low | High | High |
| TCGA-DD-AACW-01A | 0 | TCGA-DD-AACW | 1424 | 43 | male | Primary Tumor | G3 | stage i | 65 |  | WT | 0 | 0 | 0.317237 | 0 | 0.002594 | 0 | 0.680169 | 0.249 | 0.17008 | 1.021974 | cluster 2 | High | High | Low |
| TCGA-ZS-A9CD-01A | 1 | TCGA-ZS-A9CD | 1386 | 73 | male | Primary Tumor | G2 | stage ii | NA | 5 - Nodular Formation and Incomplete Cirrhosis | WT | 0 | 0 | 0.294892 | 0 | 0 | 0.012982 | 0.692126 | 0.382 | 0.067744 | 1.071412 | cluster 1 | High | Low | Low |
| TCGA-UB-A7MF-01A | 1 | TCGA-UB-A7MF | 214 | 56 | male | Primary Tumor | G2 | stage iiia | 95 | 6 - Established Cirrhosis | Mut | 0.032998 | 0 | 0.564665 | 0 | 0.020618 | 0 | 0.381718 | 0.369 | 0.075094 | 1.056491 | cluster 2 | Low | High | High |
| TCGA-G3-A5SI-01A | 1 | TCGA-G3-A5SI | 768 | 44 | male | Primary Tumor | G2 | stage ii | 74 | 6 - Established Cirrhosis | WT | 0 | 0 | 0.366619 | 0 | 0 | 0 | 0.633381 | 0.32 | 0.109858 | 1.046694 | cluster 1 | High | High | Low |
| TCGA-CC-A8HV-01A | 1 | TCGA-CC-A8HV | 279 | 51 | female | Primary Tumor | G2 | stage ii | 51 |  | Mut | 0 | 0 | 0.285917 | 0 | 0.006113 | 0.00265 | 0.70532 | 0.24 | 0.180414 | 1.018708 | cluster 2 | High | Low | Low |
| TCGA-DD-AADQ-01A | 0 | TCGA-DD-AADQ | 436 | 59 | male | Primary Tumor | G3 | stage ii | 64 |  | WT | 0 | 0 | 0.311743 | 0 | 0 | 0 | 0.688257 | 0.434 | 0.040561 | 1.078227 | cluster 1 | High | Low | Low |
| TCGA-2Y-A9H6-01A | 0 | TCGA-2Y-A9H6 | 357 | 68 | female | Primary Tumor | G2 | stage i | 99 |  | WT | 0 | 0 | 0.298705 | 0 | 0 | 0.002553 | 0.698743 | 0.308 | 0.119156 | 1.045797 | cluster 1 | High | Low | Low |
| TCGA-DD-A1EA-01A | 0 | TCGA-DD-A1EA | 2415 | 68 | male | Primary Tumor | G2 | stage ii | 94 | 1,2 - Portal Fibrosis | WT | 0 | 0 | 0.382742 | 0 | 0 | 0 | 0.617258 | 0.54 | -0.00289 | 1.092453 | cluster 1 | High | High | Low |
| TCGA-5R-AA1C-01A | 0 | TCGA-5R-AA1C | 520 | 57 | male | Primary Tumor | G2 | stage ii | 90 | 1,2 - Portal Fibrosis | WT | 0 | 0 | 0.094897 | 0 | 0 | 0 | 0.905103 | 0.475 | 0.02429 | 1.104335 | cluster 1 | High | Low | Low |
| TCGA-DD-A113-01A | 0 | TCGA-DD-A113 | 2425 | 55 | female | Primary Tumor | G3 | stage ii | 56 | 0 - No Fibrosis | WT | 0 | 0 | 0.373941 | 0 | 0 | 0 | 0.626059 | 0.306 | 0.120947 | 1.04182 | cluster 1 | High | High | Low |
| TCGA-2Y-A9H9-01A | 0 | TCGA-2Y-A9H9 | 697 | 70 | male | Primary Tumor | G2 | stage i | 88 |  | WT | 0 | 0 | 0.390385 | 0 | 0 | 0 | 0.609615 | 0.371 | 0.074219 | 1.060852 | cluster 1 | High | High | Low |
| TCGA-CC-A7IE-01A | 1 | TCGA-CC-A7IE | 217 | 57 | male | Primary Tumor | G2 | stage iiia | 62 |  | WT | 0 | 0 | 0.377745 | 0 | 0 | 0.008946 | 0.613309 | 0.372 | 0.073415 | 1.064096 | cluster 1 | High | High | Low |
| TCGA-XR-A8TD-01A | 0 | TCGA-XR-A8TD | 1030 | 49 | female | Primary Tumor | G3 | stage iiib | 69 | 0 - No Fibrosis | WT | 0.265799 | 0 | 0.449566 | 0 | 0.051591 | 0 | 0.233045 | 0.479 | 0.022502 | 1.06556 | cluster 2 | Low | High | High |
| TCGA-DD-AAD2-01A | 0 | TCGA-DD-AAD2 | 658 | 66 | male | Primary Tumor | G2 | stage i | 52 | 1,2 - Portal Fibrosis | WT | 0 | 0 | 0.308475 | 0 | 0 | 0 | 0.691525 | 0.388 | 0.062903 | 1.068953 | cluster 1 | High | Low | Low |
| TCGA-DD-AACY-01A | 0 | TCGA-DD-AACY | 1450 | 61 | male | Primary Tumor | G3 | stage i | 62 | 3,4 - Fibrous Speta | WT | 0 | 0 | 0.292336 | 0 | 0 | 0.001919 | 0.705745 | 0.372 | 0.072709 | 1.066164 | cluster 1 | High | Low | Low |
| TCGA-ED-A4XI-01A | 0 | TCGA-ED-A4XI | 819 | 58 | male | Primary Tumor | G3 | stage ii | 55 |  | WT | 0 | 0 | 0.358899 | 0 | 0 | 0 | 0.641101 | 0.31 | 0.115883 | 1.044369 | cluster 1 | High | High | Low |
| TCGA-CC-A9FW-01A | 0 | TCGA-CC-A9FW | 248 | 68 | male | Primary Tumor | G2 | stage iiia | 67 |  | WT | 0 | 0 | 0.302769 | 0 | 0 | 0.002603 | 0.694627 | 0.36 | 0.083619 | 1.06109 | cluster 1 | High | Low | Low |
| TCGA-DD-A4NP-01A | 0 | TCGA-DD-A4NP | 3308 | 32 | male | Primary Tumor | G3 | stage i | 79 | 0 - No Fibrosis | WT | 0 | 0 | 0.316652 | 0 | 0 | 0 | 0.683348 | 0.367 | 0.078016 | 1.062105 | cluster 1 | High | High | Low |
| TCGA-G3-A5SJ-01A | 0 | TCGA-G3-A5SJ | 698 | 59 | male | Primary Tumor | G2 | stage i | 87 | 6 - Established Cirrhosis | Mut | 0.453438 | 0 | 0.352594 | 0 | 0.193968 | 0 | 0 | 0.385 | 0.063848 | 1.062362 | cluster 2 | Low | High | High |
| TCGA-DD-AAE2-01A | 0 | TCGA-DD-AAE2 | 638 | 51 | male | Primary Tumor | G3 | stage i | 82 | 6 - Established Cirrhosis | WT | 0 | 0 | 0.427113 | 0 | 0.021911 | 0 | 0.550976 | 0.404 | 0.05292 | 1.067713 | cluster 2 | High | High | Low |
| TCGA-DD-AAEK-01A | 0 | TCGA-DD-AAEK | 1067 | 51 | male | Primary Tumor | G3 | stage ii | 64 | 6 - Established Cirrhosis | WT | 0 | 0 | 0.280896 | 0 | 0 | 0 | 0.719104 | 0.387 | 0.063204 | 1.070389 | cluster 1 | High | Low | Low |
| TCGA-EP-A2KC-01A | 1 | TCGA-EP-A2KC | 19 | 62 | male | Primary Tumor | G3 | stage i | NA |  | WT | 0 | 0 | 0.328115 | 0 | 0 | 0 | 0.671885 | 0.346 | 0.095557 | 1.05411 | cluster 1 | High | High | Low |
| TCGA-2Y-A9GS-01A | 1 | TCGA-2Y-A9GS | 724 | 58 | male | Primary Tumor | G2 | not reported | 92 |  | Mut | 0 | 0 | 0.379422 | 0 | 0.052754 | 0 | 0.567824 | 0.309 | 0.118152 | 1.041077 | cluster 2 | High | High | Low |
| TCGA-DD-A4NE-01A | 1 | TCGA-DD-A4NE | 660 | 75 | female | Primary Tumor | G3 | stage iiia | 79 | 0 - No Fibrosis | WT | 0 | 0 | 0.393096 | 0 | 0.038174 | 0 | 0.56873 | 0.393 | 0.058595 | 1.065699 | cluster 2 | High | High | Low |
| TCGA-DD-AADC-01A | 1 | TCGA-DD-AADC | 425 | 53 | male | Primary Tumor | G3 | stage i | 81 |  | Mut | 0 | 0 | 0.305899 | 0 | 0.019782 | 0 | 0.674318 | 0.271 | 0.152028 | 1.029546 | cluster 2 | High | Low | Low |
| TCGA-DD-AAVZ-01A | 0 | TCGA-DD-AAVZ | 1900 | 38 | male | Primary Tumor | G2 | stage i | 62 | 6 - Established Cirrhosis | WT | 0 | 0 | 0.290111 | 0 | 0 | 0.00349 | 0.706399 | 0.288 | 0.135689 | 1.039096 | cluster 1 | High | Low | Low |
| TCGA-CC-A3MA-01A | 1 | TCGA-CC-A3MA | 303 | 61 | male | Primary Tumor | G2 | stage iiia | 66 |  | Mut | 0 | 0 | 0.311874 | 0 | 0.058832 | 0 | 0.629294 | 0.329 | 0.105256 | 1.048195 | cluster 2 | High | Low | Low |
| TCGA-2Y-A9GY-01A | 1 | TCGA-2Y-A9GY | 757 | 64 | female | Primary Tumor | G3 | stage ii | 92 |  | Mut | 0.744956 | 0 | 0.123773 | 0 | 0.102259 | 0.029012 | 0 | 0.276 | 0.142103 | 1.058643 | cluster 2 | Low | Low | High |
| TCGA-ED-A627-01A | 0 | TCGA-ED-A627 | 423 | 74 | male | Primary Tumor | G2 | stage i | 74 |  | WT | 0.051891 | 0 | 0.326634 | 0 | 0 | 0 | 0.621476 | 0.49 | 0.017179 | 1.081461 | cluster 1 | High | High | High |
| TCGA-2Y-A9GW-01A | 1 | TCGA-2Y-A9GW | 1271 | 64 | male | Primary Tumor | G2 | stage i | 139 |  | WT | 0 | 0 | 0.278889 | 0 | 0 | 0.017376 | 0.703735 | 0.383 | 0.067107 | 1.07399 | cluster 1 | High | Low | Low |
| TCGA-DD-AADO-01A | 0 | TCGA-DD-AADO | 453 | 55 | male | Primary Tumor | G3 | stage i | 58 |  | WT | 0 | 0 | 0.339702 | 0 | 0 | 0 | 0.660298 | 0.319 | 0.110454 | 1.047302 | cluster 1 | High | High | Low |
| TCGA-DD-AADP-01A | 0 | TCGA-DD-AADP | 458 | 45 | male | Primary Tumor | G3 | stage i | 67 |  | WT | 0 | 0 | 0.30397 | 0 | 0 | 0.004271 | 0.691759 | 0.303 | 0.122943 | 1.044331 | cluster 1 | High | Low | Low |
| TCGA-DD-AADK-01A | 0 | TCGA-DD-AADK | 1049 | 68 | female | Primary Tumor | G3 | stage ii | 51 | 6 - Established Cirrhosis | WT | 0 | 0 | 0.330555 | 0 | 0 | 0 | 0.669445 | 0.272 | 0.147942 | 1.031425 | cluster 1 | High | High | Low |
| TCGA-G3-AAV7-01A | 0 | TCGA-G3-AAV7 | 361 | 38 | male | Primary Tumor | G2 | stage ii | 58 | 5 - Nodular Formation and Incomplete Cirrhosis | Mut | 0 | 0 | 0.320761 | 0 | 0.286442 | 0 | 0.392798 | 0.412 | 0.049239 | 1.075418 | cluster 2 | Low | High | Low |
| TCGA-DD-A1EI-01A | 0 | TCGA-DD-A1EI | 183 | 46 | male | Primary Tumor | G2 | stage i | 69 | 6 - Established Cirrhosis | Mut | 0 | 0 | 0.325252 | 0 | 0 | 0 | 0.674748 | 0.247 | 0.174896 | 1.019742 | cluster 1 | High | High | Low |
| TCGA-DD-AACA-02A | 0 | TCGA-DD-AACA | 2301 | 65 | male | Recurrent Tumor | G3 | stage i | 70 | 6 - Established Cirrhosis | WT | 0 | 0 | 0.279657 | 0 | 0 | 0 | 0.720343 | 0.349 | 0.092918 | 1.057574 | cluster 1 | High | Low | Low |
| TCGA-KR-A7K0-01A | 1 | TCGA-KR-A7K0 | 65 | 65 | male | Primary Tumor | G1 | stage i | 66 |  | WT | 0 | 0 | 0.17021 | 0 | 0 | 0 | 0.82979 | 0.368 | 0.077728 | 1.072075 | cluster 1 | High | Low | Low |
| TCGA-DD-AACC-01A | 1 | TCGA-DD-AACC | 1685 | 61 | male | Primary Tumor | G2 | stage i | 61 | 6 - Established Cirrhosis | WT | 0 | 0 | 0.466229 | 0 | 0 | 0.00781 | 0.525961 | 0.632 | -0.03047 | 1.103078 | cluster 1 | High | High | Low |
| TCGA-BC-A3KG-01A | 0 | TCGA-BC-A3KG | 680 | 68 | female | Primary Tumor | G3 | stage ii | 64 |  | WT | 0 | 0 | 0.33077 | 0 | 0.078345 | 0 | 0.590885 | 0.475 | 0.024373 | 1.080767 | cluster 2 | High | High | Low |
| TCGA-G3-AAV1-01A | 1 | TCGA-G3-AAV1 | 359 | 51 | male | Primary Tumor | G3 | stage iiic | 99 | 6 - Established Cirrhosis | WT | 0 | 0 | 0.203383 | 0 | 0 | 0 | 0.796617 | 0.372 | 0.072691 | 1.071614 | cluster 1 | High | Low | Low |
| TCGA-CC-A7IK-01A | 1 | TCGA-CC-A7IK | 262 | 59 | male | Primary Tumor | G3 | stage iiia | 57 |  | Mut | 0 | 0 | 0.278153 | 0 | 0 | 0 | 0.721847 | 0.322 | 0.108818 | 1.050689 | cluster 1 | High | Low | Low |
| TCGA-G3-A25S-01A | 1 | TCGA-G3-A25S | 416 | 64 | male | Primary Tumor | G2 | stage i | 73 | 6 - Established Cirrhosis | WT | 0 | 0 | 0.19414 | 0 | 0.003794 | 0.009441 | 0.792625 | 0.283 | 0.138944 | 1.044141 | cluster 2 | High | Low | Low |
| TCGA-DD-AADR-01A | 0 | TCGA-DD-AADR | 2028 | 58 | male | Primary Tumor | G3 | stage i | 75 | 6 - Established Cirrhosis | Mut | 0 | 0 | 0.290406 | 0 | 0 | 0 | 0.709594 | 0.314 | 0.111904 | 1.048726 | cluster 1 | High | Low | Low |
| TCGA-UB-A7MD-01A | 1 | TCGA-UB-A7MD | 52 | 67 | male | Primary Tumor | G3 | stage i | 57 | 6 - Established Cirrhosis | Mut | 0 | 0 | 0.287507 | 0 | 0 | 0 | 0.712493 | 0.384 | 0.065251 | 1.069115 | cluster 1 | High | Low | Low |
| TCGA-DD-A4NS-01A | 1 | TCGA-DD-A4NS | 2456 | 61 | female | Primary Tumor | G2 | stage i | 58 | 0 - No Fibrosis | WT | 0 | 0 | 0.307399 | 0 | 0 | 0 | 0.692601 | 0.36 | 0.084021 | 1.059985 | cluster 1 | High | Low | Low |
| TCGA-DD-AAE3-01A | 0 | TCGA-DD-AAE3 | 566 | 50 | male | Primary Tumor | G2 | stage i | 73 | 1,2 - Portal Fibrosis | WT | 0 | 0 | 0.236681 | 0 | 0 | 0.008282 | 0.755037 | 0.366 | 0.081173 | 1.067687 | cluster 1 | High | Low | Low |
| TCGA-DD-AACP-01A | 0 | TCGA-DD-AACP | 415 | 64 | male | Primary Tumor | G3 | stage i | 62 |  | WT | 0 | 0 | 0.311006 | 0 | 0.007382 | 0 | 0.681612 | 0.272 | 0.148045 | 1.031667 | cluster 2 | High | Low | Low |
| TCGA-DD-A115-01A | 1 | TCGA-DD-A115 | 2542 | 53 | male | Primary Tumor | G2 | stage iiia | 79 | 0 - No Fibrosis | WT | 0 | 0 | 0.311843 | 0 | 0.076691 | 0 | 0.611466 | 0.391 | 0.060409 | 1.066683 | cluster 2 | High | Low | Low |
| TCGA-2Y-A9H8-01A | 1 | TCGA-2Y-A9H8 | 633 | 85 | female | Primary Tumor | G2 | not reported | 40 |  | WT | 0 | 0 | 0.300977 | 0 | 0.045004 | 0 | 0.654019 | 0.303 | 0.123173 | 1.041342 | cluster 2 | High | Low | Low |
| TCGA-FV-A3I1-01A | 1 | TCGA-FV-A3I1 | 247 | 81 | female | Primary Tumor | G2 | stage ii | NA | 0 - No Fibrosis | Mut | 0 | 0 | 0.237779 | 0 | 0.003097 | 0.005613 | 0.753512 | 0.275 | 0.143957 | 1.03826 | cluster 2 | High | Low | Low |
| TCGA-2Y-A9H7-01A | 0 | TCGA-2Y-A9H7 | 1168 | 81 | female | Primary Tumor | G2 | stage i | 104 | 3,4 - Fibrous Speta | WT | 0 | 0 | 0.290947 | 0 | 0.016069 | 0 | 0.692983 | 0.292 | 0.131964 | 1.039092 | cluster 2 | High | Low | Low |
| TCGA-DD-AAVU-01A | 0 | TCGA-DD-AAVU | 2202 | 46 | male | Primary Tumor | G2 | stage ii | 88 | 3,4 - Fibrous Speta | WT | 0 | 0 | 0.307141 | 0 | 0 | 0.009438 | 0.683421 | 0.306 | 0.121275 | 1.046245 | cluster 1 | High | Low | Low |
| TCGA-BC-A110-01A | 1 | TCGA-BC-A110 | 2116 | 51 | female | Primary Tumor | G1 | not reported | 91 |  | WT | 0 | 0 | 0.284017 | 0 | 0 | 0 | 0.715983 | 0.389 | 0.061847 | 1.070785 | cluster 1 | High | Low | Low |
| TCGA-ZP-A9CZ-01A | 0 | TCGA-ZP-A9CZ | 706 | 72 | male | Primary Tumor | G1 | not reported | 73 | 3,4 - Fibrous Speta | WT | 0.104375 | 0 | 0.26103 | 0 | 0.055735 | 0 | 0.57886 | 0.433 | 0.04098 | 1.068397 | cluster 2 | High | Low | High |
| TCGA-DD-AAE1-01A | 0 | TCGA-DD-AAE1 | 552 | 52 | male | Primary Tumor | G3 | stage i | 62 | 6 - Established Cirrhosis | WT | 0 | 0 | 0.310895 | 0 | 0 | 0 | 0.689105 | 0.271 | 0.150971 | 1.030731 | cluster 1 | High | Low | Low |
| TCGA-GJ-A3OU-01A | 0 | TCGA-GJ-A3OU | 879 | 59 | male | Primary Tumor | G2 | stage i | 172 |  | Mut | 0.032367 | 0 | 0.256584 | 0 | 0.006832 | 0 | 0.704217 | 0.382 | 0.067714 | 1.066087 | cluster 2 | High | Low | High |
| TCGA-DD-A4NK-01A | 1 | TCGA-DD-A4NK | 1210 | 80 | female | Primary Tumor | G2 | stage iiia | 84 | 0 - No Fibrosis | WT | 0 | 0 | 0.313175 | 0 | 0 | 0 | 0.686825 | 0.353 | 0.088923 | 1.057606 | cluster 1 | High | High | Low |
| TCGA-RC-A7SB-01A | 0 | TCGA-RC-A7SB | 588 | 53 | male | Primary Tumor | G2 | stage ii | 61 | 0 - No Fibrosis | Mut | 0 | 0 | 0.313713 | 0 | 0 | 0 | 0.686287 | 0.322 | 0.108835 | 1.049009 | cluster 1 | High | High | Low |
| TCGA-FV-A3I0-01A | 0 | TCGA-FV-A3I0 | 848 | 76 | female | Primary Tumor | G2 | stage ii | NA |  | WT | 0 | 0 | 0.316658 | 0 | 0.503335 | 0 | 0.180007 | 0.299 | 0.126566 | 1.062486 | cluster 2 | Low | High | Low |
| TCGA-ED-A66X-01A | 0 | TCGA-ED-A66X | 406 | 35 | male | Primary Tumor | G3 | stage iiia | 61 |  | WT | 0 | 0 | 0.342296 | 0 | 0 | 0 | 0.657704 | 0.27 | 0.153099 | 1.028852 | cluster 1 | High | High | Low |
| TCGA-2Y-A9GT-01A | 1 | TCGA-2Y-A9GT | 1624 | 51 | male | Primary Tumor | G2 | stage i | 122 |  | WT | 0 | 0 | 0.352534 | 0 | 0 | 0 | 0.647466 | 0.354 | 0.0881 | 1.05632 | cluster 1 | High | High | Low |
| TCGA-FV-A2QQ-01A | 0 | TCGA-FV-A2QQ | 729 | 80 | male | Primary Tumor | G2 | stage i | NA |  | WT | 0 | 0 | 0.291658 | 0 | 0 | 0 | 0.708342 | 0.301 | 0.126025 | 1.042472 | cluster 1 | High | Low | Low |
| TCGA-DD-AAEH-01A | 0 | TCGA-DD-AAEH | 784 | 73 | male | Primary Tumor | G2 | stage i | 62 | 1,2 - Portal Fibrosis | WT | 0 | 0 | 0.194775 | 0 | 0 | 0 | 0.805225 | 0.303 | 0.124449 | 1.048719 | cluster 1 | High | Low | Low |
| TCGA-G3-A3CK-01A | 0 | TCGA-G3-A3CK | 585 | 61 | male | Primary Tumor | G2 | stage i | 67 | 6 - Established Cirrhosis | WT | 0 | 0 | 0.276668 | 0 | 0 | 0 | 0.723332 | 0.449 | 0.034405 | 1.083009 | cluster 1 | High | Low | Low |
| TCGA-EP-A26S-01A | 0 | TCGA-EP-A26S | 608 | 70 | male | Primary Tumor | G2 | stage i | 41 | 0 - No Fibrosis | WT | 0 | 0 | 0.293799 | 0 | 0 | 0 | 0.706201 | 0.372 | 0.073505 | 1.065211 | cluster 1 | High | Low | Low |
| TCGA-DD-A11A-01A | 1 | TCGA-DD-A11A | 79 | 67 | male | Primary Tumor | G3 | stage i | 67 | 0 - No Fibrosis | Mut | 0 | 0 | 0.308494 | 0 | 0 | 0 | 0.691506 | 0.294 | 0.13009 | 1.039983 | cluster 1 | High | Low | Low |
| TCGA-DD-AAE6-01A | 0 | TCGA-DD-AAE6 | 141 | 59 | female | Primary Tumor | G2 | stage i | 46 | 6 - Established Cirrhosis | Mut | 0 | 0 | 0.309524 | 0 | 0 | 0 | 0.690476 | 0.293 | 0.130481 | 1.039772 | cluster 1 | High | Low | Low |
| TCGA-XR-A8TE-01A | 0 | TCGA-XR-A8TE | 925 | 16 | male | Primary Tumor | G1 | stage iiia | 43 | 0 - No Fibrosis | WT | 0 | 0 | 0.316629 | 0 | 0 | 0.003677 | 0.679694 | 0.4 | 0.055595 | 1.072685 | cluster 1 | High | High | Low |
| TCGA-DD-A73B-01A | 1 | TCGA-DD-A73B | 283 | 72 | female | Primary Tumor | G2 | stage i | 74 | 6 - Established Cirrhosis | WT | 0 | 0 | 0.28176 | 0 | 0 | 0.014159 | 0.704081 | 0.323 | 0.107848 | 1.054692 | cluster 1 | High | Low | Low |
| TCGA-EP-A3JL-01A | 0 | TCGA-EP-A3JL | 303 | 76 | male | Primary Tumor | G2 | stage i | 73 |  | WT | 0 | 0 | 0.298158 | 0 | 0 | 0.01335 | 0.688492 | 0.368 | 0.075959 | 1.067744 | cluster 1 | High | Low | Low |
| TCGA-CC-A3MB-01A | 1 | TCGA-CC-A3MB | 315 | 36 | male | Primary Tumor | G1 | stage iiia | 58 |  | Mut | 0 | 0 | 0.294402 | 0 | 0.023906 | 0 | 0.681693 | 0.288 | 0.135579 | 1.037014 | cluster 2 | High | Low | Low |
| TCGA-DD-AAD0-01A | 0 | TCGA-DD-AAD0 | 137 | 73 | female | Primary Tumor | G2 | stage i | 54 |  | WT | 0 | 0 | 0.274854 | 0 | 0 | 0 | 0.725146 | 0.368 | 0.076719 | 1.064902 | cluster 1 | High | Low | Low |
| TCGA-MI-A75I-01A | 0 | TCGA-MI-A75I | 630 | 61 | male | Primary Tumor | G1 | not reported | 100 |  | Mut | 0 | 0 | 0.281337 | 0 | 0 | 0.015152 | 0.70351 | 0.271 | 0.149107 | 1.036288 | cluster 1 | High | Low | Low |
| TCGA-CC-A8HU-01A | 1 | TCGA-CC-A8HU | 344 | 39 | female | Primary Tumor | G3 | stage iiia | 42 |  | Mut | 0 | 0 | 0.307355 | 0 | 0.062689 | 0 | 0.629955 | 0.313 | 0.112941 | 1.044952 | cluster 2 | High | Low | Low |
| TCGA-RC-A6M5-01A | 0 | TCGA-RC-A6M5 | 15 | 20 | female | Primary Tumor | G2 | stage iva | NA |  | WT | 0 | 0 | 0.433093 | 0 | 0 | 0 | 0.566907 | 0.478 | 0.022721 | 1.080435 | cluster 1 | High | High | Low |
| TCGA-2Y-A9GZ-01A | 1 | TCGA-2Y-A9GZ | 848 | 82 | female | Primary Tumor | G2 | stage ii | 51 |  | WT | 0 | 0 | 0.379453 | 0 | 0.030323 | 0 | 0.590223 | 0.384 | 0.064878 | 1.063778 | cluster 2 | High | High | Low |
| TCGA-DD-AACO-01A | 0 | TCGA-DD-AACO | 1876 | 40 | male | Primary Tumor | G3 | stage i | 43 | 6 - Established Cirrhosis | WT | 0 | 0 | 0.372937 | 0 | 0 | 0 | 0.627063 | 0.33 | 0.104769 | 1.048657 | cluster 1 | High | High | Low |
| TCGA-LG-A6GG-01A | 0 | TCGA-LG-A6GG | 387 | 79 | female | Primary Tumor | G2 | stage ii | 65 |  | WT | 0 | 0 | 0.181009 | 0 | 0.039776 | 0 | 0.779215 | 0.408 | 0.050315 | 1.080877 | cluster 2 | High | Low | Low |
| TCGA-ED-A7PZ-01A | 0 | TCGA-ED-A7PZ | 6 | 61 | male | Primary Tumor | G2 | stage ii | 50 |  | Mut | 0 | 0 | 0.281692 | 0 | 0 | 0 | 0.718308 | 0.236 | 0.184229 | 1.016872 | cluster 1 | High | Low | Low |
| TCGA-DD-A4NV-01A | 0 | TCGA-DD-A4NV | 2398 | 61 | male | Primary Tumor | G1 | stage iiia | 129 | 0 - No Fibrosis | WT | 0 | 0 | 0.294274 | 0 | 0 | 0 | 0.705726 | 0.291 | 0.133333 | 1.03914 | cluster 1 | High | Low | Low |
| TCGA-DD-A73G-01A | 0 | TCGA-DD-A73G | 3478 | 73 | female | Primary Tumor | G3 | stage i | 44 | 0 - No Fibrosis | WT | 0 | 0 | 0.31763 | 0 | 0 | 0 | 0.68237 | 0.294 | 0.130461 | 1.039473 | cluster 1 | High | High | Low |
| TCGA-G3-AAV3-01A | 0 | TCGA-G3-AAV3 | 412 | 58 | female | Primary Tumor | G2 | stage ii | 85 | 6 - Established Cirrhosis | WT | 0 | 0 | 0.322792 | 0 | 0 | 0.017846 | 0.659362 | 0.834 | -0.08581 | 1.136447 | cluster 1 | High | High | Low |
| TCGA-BC-A5W4-01A | 1 | TCGA-BC-A5W4 | 547 | 69 | male | Primary Tumor | G3 | stage iiia | 106 |  | Mut | 0 | 0 | 0.300568 | 0 | 0 | 0 | 0.699432 | 0.247 | 0.173773 | 1.020954 | cluster 1 | High | Low | Low |
| TCGA-UB-AA0U-01A | 0 | TCGA-UB-AA0U | 327 | 60 | male | Primary Tumor | G2 | stage ii | 93 | 0 - No Fibrosis | WT | 0 | 0 | 0.338424 | 0 | 0 | 0 | 0.661576 | 0.289 | 0.134483 | 1.037024 | cluster 1 | High | High | Low |
| TCGA-EP-A12J-01A | 0 | TCGA-EP-A12J | 570 | 62 | male | Primary Tumor | G1 | stage i | NA |  | WT | 0 | 0 | 0.285501 | 0 | 0 | 0 | 0.714499 | 0.357 | 0.08582 | 1.060344 | cluster 1 | High | Low | Low |
| TCGA-WJ-A86L-01A | 0 | TCGA-WJ-A86L | 345 | 68 | female | Primary Tumor | G2 | stage i | 56 | 0 - No Fibrosis | Mut | 0 | 0 | 0.208912 | 0 | 0.005368 | 0.008963 | 0.776756 | 0.261 | 0.160272 | 1.03299 | cluster 2 | High | Low | Low |
| TCGA-DD-AACA-02B | 0 | TCGA-DD-AACA | 2301 | 65 | male | Recurrent Tumor | G3 | stage i | 70 | 6 - Established Cirrhosis | WT | 0 | 0 | 0.278571 | 0 | 0 | 0.015973 | 0.705455 | 0.391 | 0.060245 | 1.076602 | cluster 1 | High | Low | Low |
| TCGA-FV-A4ZQ-01A | 0 | TCGA-FV-A4ZQ | 12 | 52 | male | Primary Tumor | G2 | stage i | NA |  | Mut | 0.207967 | 0 | 0.435944 | 0 | 0 | 0 | 0.356089 | 0.575 | -0.01549 | 1.080056 | cluster 1 | Low | High | High |
| TCGA-WX-AA46-01A | 0 | TCGA-WX-AA46 | 756 | 61 | male | Primary Tumor | G1 | stage ii | 75 | 0 - No Fibrosis | WT | 0 | 0 | 0.288415 | 0 | 0 | 0 | 0.711585 | 0.337 | 0.101207 | 1.053497 | cluster 1 | High | Low | Low |
| TCGA-DD-A4ND-01A | 0 | TCGA-DD-A4ND | 2746 | 56 | female | Primary Tumor | G3 | stage i | 89 | 0 - No Fibrosis | WT | 0 | 0 | 0.378332 | 0 | 0 | 0 | 0.621668 | 0.331 | 0.103873 | 1.048885 | cluster 1 | High | High | Low |
| TCGA-ED-A8O5-01A | 0 | TCGA-ED-A8O5 | 406 | 59 | female | Primary Tumor | G3 | stage iiia | 63 |  | WT | 0 | 0 | 0.313925 | 0 | 0.073674 | 0 | 0.612401 | 0.331 | 0.10345 | 1.048487 | cluster 2 | High | High | Low |
| TCGA-UB-A7ME-01A | 0 | TCGA-UB-A7ME | 486 | 51 | male | Primary Tumor | G2 | stage i | 75 | 1,2 - Portal Fibrosis | WT | 0 | 0 | 0.236687 | 0 | 0.024239 | 0.012437 | 0.726637 | 0.271 | 0.150811 | 1.035798 | cluster 2 | High | Low | Low |
| TCGA-CC-5264-01A | 1 | TCGA-CC-5264 | 102 | 71 | male | Primary Tumor | G2 | stage iiia | 52 |  | WT | 0 | 0 | 0.299396 | 0 | 0.101008 | 0 | 0.599596 | 0.308 | 0.119873 | 1.041397 | cluster 2 | High | Low | Low |
| TCGA-G3-A7M7-01A | 0 | TCGA-G3-A7M7 | 361 | 65 | male | Primary Tumor | G1 | stage i | 77 | 3,4 - Fibrous Speta | WT | 0 | 0 | 0.303682 | 0 | 0 | 0 | 0.696318 | 0.368 | 0.075556 | 1.063803 | cluster 1 | High | Low | Low |
| TCGA-DD-AACS-01A | 0 | TCGA-DD-AACS | 1804 | 39 | male | Primary Tumor | G3 | stage i | 80 |  | Mut | 0 | 0 | 0.267329 | 0 | 0.013879 | 0.011428 | 0.707364 | 0.256 | 0.164241 | 1.028391 | cluster 2 | High | Low | Low |
| TCGA-DD-AAE0-01A | 0 | TCGA-DD-AAE0 | 555 | 45 | female | Primary Tumor | G4 | stage iiia | 56 | 3,4 - Fibrous Speta | Mut | 0.088398 | 0 | 0.397498 | 0 | 0.080676 | 0 | 0.433429 | 0.303 | 0.123521 | 1.032324 | cluster 2 | Low | High | High |
| TCGA-G3-A5SK-01A | 0 | TCGA-G3-A5SK | 744 | 58 | male | Primary Tumor | G1 | stage i | 67 | 6 - Established Cirrhosis | WT | 0 | 0 | 0.295849 | 0 | 0 | 0.004092 | 0.700059 | 0.441 | 0.038465 | 1.081283 | cluster 1 | High | Low | Low |
| TCGA-BC-A69I-01A | 0 | TCGA-BC-A69I | 387 | 69 | male | Primary Tumor | G1 | stage i | 95 |  | WT | 0 | 0 | 0.262456 | 0 | 0 | 0 | 0.737544 | 0.351 | 0.090051 | 1.059812 | cluster 1 | High | Low | Low |
| TCGA-BC-A10R-01A | 1 | TCGA-BC-A10R | 308 | 66 | female | Primary Tumor | G2 | not reported | NA |  | Mut | 0 | 0 | 0.309159 | 0 | 0 | 0 | 0.690841 | 0.383 | 0.066565 | 1.067358 | cluster 1 | High | Low | Low |
| TCGA-5C-AAPD-01A | 0 | TCGA-5C-AAPD | 20 | 61 | male | Primary Tumor | G1 | stage ii | 69 |  | Mut | 0.502448 | 0 | 0.329964 | 0 | 0.16193 | 0.005658 | 0 | 0.245 | 0.176245 | 1.018741 | cluster 2 | Low | High | High |
| TCGA-BC-A10S-01A | 1 | TCGA-BC-A10S | 1423 | 81 | male | Primary Tumor | G1 | not reported | 74 |  | WT | 0 | 0 | 0.29811 | 0 | 0 | 0.012328 | 0.689561 | 0.354 | 0.08787 | 1.062209 | cluster 1 | High | Low | Low |
| TCGA-DD-AAVX-01A | 0 | TCGA-DD-AAVX | 1718 | 38 | male | Primary Tumor | G2 | stage ii | 65 | 6 - Established Cirrhosis | WT | 0 | 0 | 0.287874 | 0 | 0 | 0.004545 | 0.707582 | 0.403 | 0.054313 | 1.07511 | cluster 1 | High | Low | Low |
| TCGA-DD-AAVQ-01A | 0 | TCGA-DD-AAVQ | 2728 | 38 | male | Primary Tumor | G2 | stage i | 72 | 6 - Established Cirrhosis | WT | 0.268485 | 0 | 0.212736 | 0 | 0.043392 | 0.005144 | 0.470242 | 0.379 | 0.070584 | 1.05351 | cluster 2 | Low | Low | High |
| TCGA-G3-A3CJ-01A | 0 | TCGA-G3-A3CJ | 594 | 52 | male | Primary Tumor | G2 | stage ii | 72 | 5 - Nodular Formation and Incomplete Cirrhosis | WT | 0 | 0 | 0.220843 | 0 | 0 | 0 | 0.779157 | 0.393 | 0.058748 | 1.076465 | cluster 1 | High | Low | Low |
| TCGA-DD-A4NF-01A | 0 | TCGA-DD-A4NF | 942 | 72 | male | Primary Tumor | G2 | stage i | 86 | 6 - Established Cirrhosis | WT | 0 | 0 | 0.154228 | 0 | 0 | 0 | 0.845772 | 0.412 | 0.049203 | 1.086543 | cluster 1 | High | Low | Low |
| TCGA-BC-A10Q-01A | 1 | TCGA-BC-A10Q | 1135 | 72 | female | Primary Tumor |  | not reported | NA |  | WT | 0 | 0 | 0.484149 | 0.181175 | 0.270349 | 0 | 0.064328 | 0.569 | -0.01369 | 1.134234 | cluster 2 | Low | High | Low |
| TCGA-DD-A1EE-01A | 1 | TCGA-DD-A1EE | 349 | 73 | male | Primary Tumor | G3 | stage iiia | 77 | 6 - Established Cirrhosis | Mut | 0 | 0 | 0.215759 | 0 | 0 | 0 | 0.784241 | 0.394 | 0.058248 | 1.077083 | cluster 1 | High | Low | Low |
| TCGA-ZP-A9CY-01A | 0 | TCGA-ZP-A9CY | 782 | 66 | female | Primary Tumor | G1 | not reported | 83 | 6 - Established Cirrhosis | WT | 0 | 0 | 0.350998 | 0 | 0 | 0 | 0.649002 | 0.369 | 0.07487 | 1.061934 | cluster 1 | High | High | Low |
| TCGA-FV-A23B-01A | 1 | TCGA-FV-A23B | 1852 | 70 | female | Primary Tumor |  | stage ii | NA |  | WT | 0 | 0 | 0.283095 | 0 | 0 | 0.006261 | 0.710645 | 0.295 | 0.12975 | 1.042732 | cluster 1 | High | Low | Low |
| TCGA-HP-A5MZ-01A | 1 | TCGA-HP-A5MZ | 91 | 78 | male | Primary Tumor | G2 | stage i | 82 |  | WT | 0 | 0 | 0.312631 | 0 | 0 | 0.007562 | 0.679808 | 0.396 | 0.057614 | 1.073189 | cluster 1 | High | Low | Low |
| TCGA-DD-AADB-01A | 0 | TCGA-DD-AADB | 1242 | 51 | male | Primary Tumor | G4 | stage i | 68 |  | Mut | 0.15155 | 0 | 0.443038 | 0 | 0.041602 | 0 | 0.36381 | 0.549 | -0.00736 | 1.078415 | cluster 2 | Low | High | High |
| TCGA-CC-A1HT-01A | 1 | TCGA-CC-A1HT | 101 | 50 | male | Primary Tumor | G3 | stage iiia | 54 |  | Mut | 0.365539 | 0 | 0.384087 | 0 | 0.231888 | 0.018486 | 0 | 0.407 | 0.051321 | 1.06899 | cluster 2 | Low | High | High |
| TCGA-DD-A11B-01A | 1 | TCGA-DD-A11B | 14 | 73 | male | Primary Tumor | G2 | stage i | 134 | 6 - Established Cirrhosis | WT | 0 | 0 | 0.311356 | 0 | 0 | 0 | 0.688644 | 0.275 | 0.14406 | 1.033755 | cluster 1 | High | Low | Low |
| TCGA-ED-A7XP-01A | 0 | TCGA-ED-A7XP | 400 | 53 | female | Primary Tumor | G3 | stage ii | 54 |  | Mut | 0 | 0 | 0.330423 | 0 | 0 | 0 | 0.669577 | 0.367 | 0.078233 | 1.061378 | cluster 1 | High | High | Low |
| TCGA-FV-A3R3-01A | 1 | TCGA-FV-A3R3 | 366 | 38 | female | Primary Tumor | G2 | stage i | 97 |  | WT | 0 | 0 | 0.291699 | 0 | 0 | 0.007937 | 0.700364 | 0.359 | 0.084539 | 1.062746 | cluster 1 | High | Low | Low |
| TCGA-HP-A5N0-01A | 1 | TCGA-HP-A5N0 | 752 | 90 | female | Primary Tumor |  | not reported | 65 | 0 - No Fibrosis | WT | 0 | 0 | 0.344798 | 0 | 0.004246 | 0 | 0.650956 | 0.314 | 0.111701 | 1.046392 | cluster 2 | High | High | Low |
| TCGA-DD-A4NI-01A | 0 | TCGA-DD-A4NI | 816 | 67 | male | Primary Tumor | G2 | stage ii | 99 | 0 - No Fibrosis | WT | 0 | 0 | 0.29524 | 0 | 0 | 0 | 0.70476 | 0.387 | 0.063364 | 1.069482 | cluster 1 | High | Low | Low |
| TCGA-EP-A2KB-01A | 1 | TCGA-EP-A2KB | 596 | 46 | female | Primary Tumor | G2 | stage i | 81 |  | WT | 0 | 0 | 0.298294 | 0 | 0 | 0 | 0.701706 | 0.269 | 0.155267 | 1.029278 | cluster 1 | High | Low | Low |
| TCGA-CC-A7IH-01A | 0 | TCGA-CC-A7IH | 365 | 58 | male | Primary Tumor | G1 | stage iiia | 56 |  | WT | 0 | 0 | 0.306858 | 0 | 0 | 0 | 0.693142 | 0.372 | 0.073569 | 1.06449 | cluster 1 | High | Low | Low |
| TCGA-ES-A2HS-01A | 1 | TCGA-ES-A2HS | 688 | 80 | male | Primary Tumor | G2 | stage i | 97 | 0 - No Fibrosis | WT | 0 | 0 | 0.315024 | 0 | 0 | 0 | 0.684976 | 0.249 | 0.171708 | 1.021433 | cluster 1 | High | High | Low |
| TCGA-G3-A3CG-01A | 0 | TCGA-G3-A3CG | 673 | 80 | male | Primary Tumor | G2 | stage i | 93 | 6 - Established Cirrhosis | WT | 0 | 0 | 0.30506 | 0 | 0 | 0 | 0.69494 | 0.503 | 0.011433 | 1.090842 | cluster 1 | High | Low | Low |
| TCGA-ED-A5KG-01A | 0 | TCGA-ED-A5KG | 854 | 60 | female | Primary Tumor | G2 | stage ii | 61 |  | WT | 0.472149 | 0 | 0.484631 | 0 | 0.006274 | 0.036311 | 0.000636 | 0.227 | 0.193063 | 1.015236 | cluster 2 | Low | High | High |
| TCGA-CC-A123-01A | 0 | TCGA-CC-A123 | 219 | 24 | female | Primary Tumor | G1 | stage iiia | NA |  | WT | 0 | 0 | 0.321267 | 0 | 0 | 0.000547 | 0.678185 | 0.275 | 0.143905 | 1.033603 | cluster 1 | High | High | Low |
| TCGA-DD-AAD1-01A | 0 | TCGA-DD-AAD1 | 564 | 51 | female | Primary Tumor | G4 | stage i | 44 |  | WT | 0.002674 | 0 | 0.332079 | 0 | 0.034711 | 0 | 0.630536 | 0.297 | 0.127078 | 1.038737 | cluster 2 | High | High | Low |
| TCGA-DD-A73D-01A | 0 | TCGA-DD-A73D | 693 | 68 | female | Primary Tumor | G1 | stage ii | 102 | 6 - Established Cirrhosis | WT | 0 | 0 | 0.310095 | 0 | 0 | 0 | 0.689905 | 0.3 | 0.126328 | 1.041562 | cluster 1 | High | Low | Low |
| TCGA-DD-AAVW-01A | 0 | TCGA-DD-AAVW | 2317 | 35 | male | Primary Tumor | G2 | stage i | 62 | 6 - Established Cirrhosis | WT | 0 | 0 | 0.296247 | 0 | 0 | 0 | 0.703753 | 0.248 | 0.173472 | 1.021229 | cluster 1 | High | Low | Low |
| TCGA-DD-AAVS-01A | 0 | TCGA-DD-AAVS | 1823 | 56 | male | Primary Tumor | G2 | stage i | 72 | 3,4 - Fibrous Speta | WT | 0 | 0 | 0.304722 | 0 | 0 | 0.011299 | 0.683979 | 0.249 | 0.170281 | 1.024823 | cluster 1 | High | Low | Low |
| TCGA-DD-AACN-01A | 0 | TCGA-DD-AACN | 1302 | 32 | male | Primary Tumor | G3 | stage i | 70 |  | WT | 0 | 0 | 0.301895 | 0 | 0 | 0 | 0.698105 | 0.337 | 0.100794 | 1.053018 | cluster 1 | High | Low | Low |
| TCGA-DD-A3A2-01A | 1 | TCGA-DD-A3A2 | 2131 | 76 | female | Primary Tumor | G1 | stage i | 85 | 0 - No Fibrosis | WT | 0 | 0 | 0.211387 | 0 | 0.013242 | 0.0044 | 0.770971 | 0.272 | 0.147027 | 1.037504 | cluster 2 | High | Low | Low |
| TCGA-K7-A5RF-01A | 0 | TCGA-K7-A5RF | 631 | 64 | male | Primary Tumor | G1 | stage i | 75 |  | WT | 0 | 0 | 0.262054 | 0 | 0 | 0.000155 | 0.737792 | 0.355 | 0.086029 | 1.061644 | cluster 1 | High | Low | Low |
| TCGA-DD-A116-01A | 1 | TCGA-DD-A116 | 1622 | 68 | male | Primary Tumor | G3 | stage iiia | 73 | 3,4 - Fibrous Speta | WT | 0 | 0 | 0.219101 | 0 | 0 | 0.018612 | 0.762287 | 0.35 | 0.091313 | 1.067356 | cluster 1 | High | Low | Low |
| TCGA-K7-A5RG-01A | 0 | TCGA-K7-A5RG | 519 | 66 | male | Primary Tumor | G1 | stage i | 123 |  | Mut | 0.43495 | 0 | 0.335186 | 0 | 0.088952 | 0 | 0.140912 | 0.24 | 0.179695 | 1.011205 | cluster 2 | Low | High | High |
| TCGA-BC-A10T-01A | 1 | TCGA-BC-A10T | 837 | 76 | male | Primary Tumor | G1 | not reported | 86 | 1,2 - Portal Fibrosis | WT | 0 | 0 | 0.325357 | 0 | 0 | 0 | 0.674643 | 0.382 | 0.067651 | 1.066089 | cluster 1 | High | High | Low |
| TCGA-DD-AADJ-01A | 0 | TCGA-DD-AADJ | 1066 | 70 | female | Primary Tumor | G3 | stage i | 59 |  | WT | 0 | 0 | 0.317476 | 0 | 0 | 0 | 0.682524 | 0.279 | 0.140387 | 1.035148 | cluster 1 | High | High | Low |
| TCGA-DD-AACE-01A | 0 | TCGA-DD-AACE | 2184 | 62 | male | Primary Tumor | G3 | stage i | 62 | 6 - Established Cirrhosis | Mut | 0 | 0 | 0.278882 | 0 | 0 | 0.015963 | 0.705154 | 0.369 | 0.075482 | 1.06983 | cluster 1 | High | Low | Low |
| TCGA-FV-A3R2-01A | 1 | TCGA-FV-A3R2 | 194 | 75 | male | Primary Tumor |  | stage i | 75 |  | Mut | 0 | 0 | 0.367522 | 0 | 0.002849 | 0 | 0.629629 | 0.308 | 0.120126 | 1.042204 | cluster 2 | High | High | Low |
| TCGA-DD-AADD-01A | 0 | TCGA-DD-AADD | 1231 | 51 | male | Primary Tumor | G4 | stage i | 68 |  | Mut | 0 | 0 | 0.302353 | 0 | 0 | 0.009319 | 0.688328 | 0.284 | 0.138621 | 1.038697 | cluster 1 | High | Low | Low |
| TCGA-DD-AACX-01A | 0 | TCGA-DD-AACX | 170 | 66 | male | Primary Tumor | G3 | stage ii | 53 | 3,4 - Fibrous Speta | WT | 0 | 0 | 0.269752 | 0 | 0 | 0 | 0.730248 | 0.404 | 0.05367 | 1.0752 | cluster 1 | High | Low | Low |
| TCGA-4R-AA8I-01A | 1 | TCGA-4R-AA8I | 262 | 66 | male | Primary Tumor | G2 | stage ii | 103 | 6 - Established Cirrhosis | WT | 0 | 0 | 0.255158 | 0 | 0 | 0 | 0.744842 | 0.312 | 0.114307 | 1.049502 | cluster 1 | High | Low | Low |
| TCGA-FV-A496-01A | 0 | TCGA-FV-A496 | 10 | 84 | female | Primary Tumor | G2 | stage i | 54 | 0 - No Fibrosis | WT | 0 | 0 | 0.32627 | 0 | 0.019421 | 0 | 0.65431 | 0.263 | 0.157779 | 1.026426 | cluster 2 | High | High | Low |
| TCGA-CC-A7IF-01A | 1 | TCGA-CC-A7IF | 649 | 59 | male | Primary Tumor | G1 | stage iiia | 58 |  | WT | 0 | 0 | 0.325155 | 0 | 0 | 0 | 0.674845 | 0.309 | 0.117316 | 1.044888 | cluster 1 | High | High | Low |
| TCGA-DD-AADS-01A | 0 | TCGA-DD-AADS | 474 | 63 | male | Primary Tumor | G2 | stage i | 53 |  | WT | 0 | 0 | 0.244596 | 0 | 0 | 0.004404 | 0.751 | 0.274 | 0.144467 | 1.037542 | cluster 1 | High | Low | Low |
| TCGA-DD-AAE9-01A | 0 | TCGA-DD-AAE9 | 722 | 69 | male | Primary Tumor | G3 | stage i | 63 | 1,2 - Portal Fibrosis | WT | 0 | 0 | 0.055898 | 0 | 0 | 0 | 0.944102 | 0.304 | 0.122581 | 1.061809 | cluster 1 | High | Low | Low |
| TCGA-ZS-A9CF-02A | 0 | TCGA-ZS-A9CF | 2412 | 64 | male | Recurrent Tumor | G2 | stage ii | NA | 0 - No Fibrosis | WT | 0 | 0 | 0.267867 | 0 | 0.004732 | 0.012834 | 0.714566 | 0.426 | 0.042883 | 1.083643 | cluster 2 | High | Low | Low |
| TCGA-NI-A4U2-01A | 1 | TCGA-NI-A4U2 | 1791 | 71 | male | Primary Tumor | G1 | stage iiia | 82 | 1,2 - Portal Fibrosis | WT | 0 | 0 | 0.324688 | 0 | 0.052136 | 0 | 0.623175 | 0.293 | 0.130711 | 1.037061 | cluster 2 | High | High | Low |
| TCGA-DD-AACI-01A | 0 | TCGA-DD-AACI | 1618 | 69 | male | Primary Tumor | G3 | stage ii | 48 | 5 - Nodular Formation and Incomplete Cirrhosis | WT | 0 | 0 | 0.35014 | 0 | 0 | 0 | 0.64986 | 0.29 | 0.134386 | 1.036722 | cluster 1 | High | High | Low |
| TCGA-ZP-A9D1-01A | 0 | TCGA-ZP-A9D1 | 21 | 56 | female | Primary Tumor | G2 | not reported | 130 | 5 - Nodular Formation and Incomplete Cirrhosis | WT | 0 | 0 | 0.317684 | 0 | 0 | 0.003152 | 0.679164 | 0.37 | 0.074839 | 1.064277 | cluster 1 | High | High | Low |
| TCGA-UB-A7MB-01A | 0 | TCGA-UB-A7MB | 601 | 24 | male | Primary Tumor | G3 | stage ii | 62 | 0 - No Fibrosis | Mut | 0 | 0 | 0.344816 | 0 | 0.106267 | 0 | 0.548917 | 0.369 | 0.075326 | 1.058816 | cluster 2 | High | High | Low |
| TCGA-2Y-A9HA-01A | 1 | TCGA-2Y-A9HA | 36 | 70 | male | Primary Tumor | G2 | stage ii | 69 | 3,4 - Fibrous Speta | Mut | 0 | 0 | 0.21805 | 0 | 0 | 0.018542 | 0.763408 | 0.34 | 0.098845 | 1.063931 | cluster 1 | High | Low | Low |
| TCGA-DD-A4NR-01A | 1 | TCGA-DD-A4NR | 9 | 85 | female | Primary Tumor | G3 | stage i | 54 | 6 - Established Cirrhosis | WT | 0 | 0 | 0.326976 | 0 | 0 | 0.011821 | 0.661203 | 0.404 | 0.052986 | 1.075741 | cluster 1 | High | High | Low |
| TCGA-RG-A7D4-01A | 0 | TCGA-RG-A7D4 | 1098 | 69 | male | Primary Tumor | G2 | stage ii | 87 |  | Mut | 0 | 0 | 0.442286 | 0 | 0 | 0 | 0.557714 | 0.896 | -0.10388 | 1.129029 | cluster 1 | High | High | Low |
| TCGA-5R-AAAM-01A | 1 | TCGA-5R-AAAM | 46 | 65 | female | Primary Tumor | G2 | stage ii | 80 | 6 - Established Cirrhosis | WT | 0 | 0 | 0.367333 | 0 | 0 | 0 | 0.632667 | 0.338 | 0.100074 | 1.050791 | cluster 1 | High | High | Low |
| TCGA-ED-A97K-01A | 0 | TCGA-ED-A97K | 6 | 54 | male | Primary Tumor | G2 | stage iiia | 64 |  | WT | 0.078283 | 0 | 0.502699 | 0 | 0 | 0 | 0.419018 | 0.547 | -0.00691 | 1.083179 | cluster 1 | Low | High | High |
| TCGA-DD-A3A4-01A | 1 | TCGA-DD-A3A4 | 612 | 37 | male | Primary Tumor | G3 | stage iiia | 65 | 0 - No Fibrosis | WT | 0 | 0 | 0.285561 | 0 | 0 | 0.00581 | 0.708629 | 0.511 | 0.008919 | 1.095075 | cluster 1 | High | Low | Low |
| TCGA-BC-A217-01A | 1 | TCGA-BC-A217 | 1397 | 75 | female | Primary Tumor | G3 | stage ii | 81 | 0 - No Fibrosis | Mut | 0.088025 | 0 | 0.432564 | 0 | 0.099665 | 0 | 0.379746 | 0.351 | 0.090012 | 1.045433 | cluster 2 | Low | High | High |
| TCGA-DD-A4NQ-01A | 1 | TCGA-DD-A4NQ | 373 | 60 | male | Primary Tumor | G3 | stage ii | 75 |  | Mut | 0 | 0 | 0.301115 | 0 | 0 | 0 | 0.698885 | 0.372 | 0.073336 | 1.064889 | cluster 1 | High | Low | Low |
| TCGA-G3-A25T-01A | 0 | TCGA-G3-A25T | 1553 | 45 | female | Primary Tumor | G2 | stage iiia | 64 | 0 - No Fibrosis | WT | 0 | 0 | 0.326219 | 0 | 0.102512 | 0 | 0.571269 | 0.354 | 0.087556 | 1.054285 | cluster 2 | High | High | Low |
| TCGA-UB-A7MC-01A | 0 | TCGA-UB-A7MC | 500 | 59 | male | Primary Tumor | G3 | stage iiia | 78 | 1,2 - Portal Fibrosis | WT | 0 | 0 | 0.300079 | 0 | 0 | 0.000741 | 0.69918 | 0.363 | 0.082291 | 1.061296 | cluster 1 | High | Low | Low |
| TCGA-MR-A8JO-01A | 0 | TCGA-MR-A8JO | 330 | 34 | male | Primary Tumor | G3 | stage i | 90 |  | WT | 0 | 0 | 0.338088 | 0 | 0.025622 | 0.001668 | 0.634622 | 0.535 | -0.00128 | 1.093285 | cluster 2 | High | High | Low |
| TCGA-DD-AADI-01A | 0 | TCGA-DD-AADI | 1085 | 43 | female | Primary Tumor | G3 | stage i | 64 |  | WT | 0 | 0 | 0.401499 | 0 | 0 | 0 | 0.598501 | 0.248 | 0.17358 | 1.019059 | cluster 1 | High | High | Low |
| TCGA-WX-AA47-01A | 1 | TCGA-WX-AA47 | 556 | 33 | female | Primary Tumor | G2 | stage iiia | 63 | 0 - No Fibrosis | WT | 0 | 0 | 0.2676 | 0 | 0 | 0.014626 | 0.717774 | 0.486 | 0.018175 | 1.095337 | cluster 1 | High | Low | Low |
| TCGA-DD-AADA-01A | 0 | TCGA-DD-AADA | 1233 | 66 | female | Primary Tumor | G3 | stage i | 59 |  | WT | 0 | 0 | 0.348459 | 0 | 0 | 0.002019 | 0.649522 | 0.325 | 0.10711 | 1.048929 | cluster 1 | High | High | Low |
| TCGA-WQ-AB4B-01A | 0 | TCGA-WQ-AB4B | 395 | 62 | male | Primary Tumor | G2 | stage ii | 122 |  | WT | 0 | 0 | 0.194319 | 0 | 0 | 0.012815 | 0.792866 | 0.337 | 0.101429 | 1.062786 | cluster 1 | High | Low | Low |
| TCGA-YA-A8S7-01A | 1 | TCGA-YA-A8S7 | 412 | 68 | male | Primary Tumor | G3 | stage iiia | 73 |  | Mut | 0 | 0 | 0.415298 | 0 | 0.158549 | 0 | 0.426152 | 0.656 | -0.0368 | 1.102856 | cluster 2 | Low | High | Low |
| TCGA-2Y-A9H2-01A | 0 | TCGA-2Y-A9H2 | 1731 | 64 | female | Primary Tumor | G3 | stage i | 55 |  | WT | 0 | 0 | 0.464769 | 0 | 0 | 0 | 0.535231 | 0.357 | 0.085834 | 1.054775 | cluster 1 | High | High | Low |
| TCGA-5C-A9VG-01A | 0 | TCGA-5C-A9VG | 328 | 58 | male | Primary Tumor | G2 | stage ii | 73 |  | WT | 0 | 0 | 0.281986 | 0 | 0 | 0.006505 | 0.711509 | 0.471 | 0.025784 | 1.088351 | cluster 1 | High | Low | Low |
| TCGA-BC-A10Y-01A | 1 | TCGA-BC-A10Y | 711 | 76 | male | Primary Tumor | G3 | not reported | 77 |  | WT | 0 | 0 | 0.328749 | 0 | 0.050145 | 0 | 0.621105 | 0.363 | 0.082149 | 1.057642 | cluster 2 | High | High | Low |
| TCGA-EP-A2KA-01A | 1 | TCGA-EP-A2KA | 627 | 52 | female | Primary Tumor | G3 | stage iiia | NA | 1,2 - Portal Fibrosis | WT | 0.005791 | 0 | 0.30295 | 0 | 0.041135 | 0 | 0.650125 | 0.303 | 0.12354 | 1.040723 | cluster 2 | High | Low | High |
| TCGA-BC-A10X-01A | 1 | TCGA-BC-A10X | 770 | 52 | female | Primary Tumor | G2 | stage iiia | 65 |  | WT | 0 | 0 | 0.356575 | 0 | 0 | 0 | 0.643425 | 0.363 | 0.082607 | 1.05848 | cluster 1 | High | High | Low |
| TCGA-DD-AADW-01A | 0 | TCGA-DD-AADW | 587 | 48 | male | Primary Tumor | G3 | stage i | 49 | 6 - Established Cirrhosis | Mut | 0 | 0 | 0.390752 | 0 | 0.043893 | 0 | 0.565355 | 0.27 | 0.15471 | 1.025794 | cluster 2 | High | High | Low |
| TCGA-DD-AAED-01A | 0 | TCGA-DD-AAED | 763 | 51 | male | Primary Tumor | G3 | stage i | 69 | 1,2 - Portal Fibrosis | Mut | 0 | 0 | 0.214213 | 0 | 0 | 0.008988 | 0.776799 | 0.228 | 0.190205 | 1.018828 | cluster 1 | High | Low | Low |
| TCGA-LG-A9QD-01A | 0 | TCGA-LG-A9QD | 366 | 68 | male | Primary Tumor | G2 | stage iiia | 74 |  | WT | 0 | 0 | 0.292462 | 0 | 0 | 0.004235 | 0.703303 | 0.413 | 0.048473 | 1.077252 | cluster 1 | High | Low | Low |
| TCGA-DD-AADV-01A | 0 | TCGA-DD-AADV | 574 | 50 | male | Primary Tumor | G3 | stage i | 64 | 6 - Established Cirrhosis | Mut | 0 | 0 | 0.336505 | 0 | 0.014764 | 0 | 0.648732 | 0.274 | 0.144246 | 1.032215 | cluster 2 | High | High | Low |
| TCGA-G3-A3CH-01A | 0 | TCGA-G3-A3CH | 780 | 53 | male | Primary Tumor | G2 | stage iiia | 74 | 6 - Established Cirrhosis | WT | 0 | 0 | 0.363425 | 0 | 0 | 0 | 0.636575 | 0.319 | 0.110457 | 1.046534 | cluster 1 | High | High | Low |
| TCGA-DD-AAW0-01A | 0 | TCGA-DD-AAW0 | 2015 | 54 | male | Primary Tumor | G2 | stage i | 75 | 3,4 - Fibrous Speta | WT | 0 | 0 | 0.318462 | 0 | 0 | 0.002344 | 0.679194 | 0.291 | 0.133762 | 1.038551 | cluster 1 | High | High | Low |
| TCGA-DD-AAW3-01A | 0 | TCGA-DD-AAW3 | 1633 | 69 | male | Primary Tumor | G2 | stage i | 64 | 0 - No Fibrosis | WT | 0 | 0 | 0.351923 | 0 | 0 | 0 | 0.648077 | 0.379 | 0.070344 | 1.063791 | cluster 1 | High | High | Low |
| TCGA-ED-A7PY-01A | 0 | TCGA-ED-A7PY | 390 | 20 | female | Primary Tumor | G3 | stage ii | 40 |  | WT | 0 | 0 | 0.270093 | 0 | 0 | 0.019132 | 0.710775 | 0.249 | 0.170051 | 1.028081 | cluster 1 | High | Low | Low |
| TCGA-ZP-A9D2-01A | 1 | TCGA-ZP-A9D2 | 765 | 51 | male | Primary Tumor | G2 | not reported | 74 | 1,2 - Portal Fibrosis | Mut | 0.005622 | 0 | 0.512697 | 0 | 0.203332 | 0 | 0.27835 | 0.368 | 0.075627 | 1.060464 | cluster 2 | Low | High | High |
| TCGA-G3-A25Y-01A | 1 | TCGA-G3-A25Y | 452 | 52 | female | Primary Tumor | G3 | stage i | 57 | 3,4 - Fibrous Speta | WT | 0 | 0 | 0.375452 | 0 | 0.001234 | 0 | 0.623314 | 0.247 | 0.173892 | 1.019149 | cluster 2 | High | High | Low |
| TCGA-G3-A3CI-01A | 0 | TCGA-G3-A3CI | 180 | 71 | male | Primary Tumor | G2 | stage i | 98 | 0 - No Fibrosis | WT | 0 | 0 | 0.402774 | 0 | 0 | 0 | 0.597226 | 0.293 | 0.130609 | 1.037184 | cluster 1 | High | High | Low |
| TCGA-ZP-A9D0-01A | 0 | TCGA-ZP-A9D0 | 1091 | 67 | female | Primary Tumor | G1 | not reported | 78 | 0 - No Fibrosis | WT | 0 | 0 | 0.319634 | 0 | 0 | 0 | 0.680366 | 0.365 | 0.081262 | 1.060581 | cluster 1 | High | High | Low |
| TCGA-EP-A3RK-01A | 0 | TCGA-EP-A3RK | 363 | 73 | male | Primary Tumor | G2 | stage iiia | 101 | 1,2 - Portal Fibrosis | WT | 0 | 0 | 0.32742 | 0 | 0 | 0.018435 | 0.654145 | 0.659 | -0.03795 | 1.116521 | cluster 1 | High | High | Low |
| TCGA-CC-A7IJ-01A | 0 | TCGA-CC-A7IJ | 382 | 56 | male | Primary Tumor | G3 | stage ii | 54 |  | Mut | 0.072547 | 0 | 0.455669 | 0 | 0.439346 | 0 | 0.032438 | 0.171 | 0.262145 | 0.990619 | cluster 2 | Low | High | High |
| TCGA-MI-A75H-01A | 0 | TCGA-MI-A75H | 747 | 77 | male | Primary Tumor |  | not reported | 84 | 6 - Established Cirrhosis | WT | 0 | 0 | 0.157925 | 0 | 0 | 0 | 0.842075 | 0.364 | 0.081886 | 1.071244 | cluster 1 | High | Low | Low |
| TCGA-G3-AAV5-01A | 0 | TCGA-G3-AAV5 | 354 | 67 | male | Primary Tumor | G2 | stage ii | 88 | 6 - Established Cirrhosis | WT | 0 | 0 | 0.335598 | 0 | 0 | 0 | 0.664402 | 0.39 | 0.061006 | 1.068407 | cluster 1 | High | High | Low |
| TCGA-ZP-A9D4-01A | 0 | TCGA-ZP-A9D4 | 395 | 64 | female | Primary Tumor | G1 | not reported | 55 | 0 - No Fibrosis | WT | 0 | 0 | 0.305506 | 0 | 0 | 0 | 0.694494 | 0.386 | 0.063583 | 1.068822 | cluster 1 | High | Low | Low |
| TCGA-DD-A1ED-01A | 0 | TCGA-DD-A1ED | 2301 | 68 | male | Primary Tumor | G1 | stage i | 85 | 0 - No Fibrosis | WT | 0 | 0 | 0.287561 | 0 | 0 | 0.00754 | 0.704899 | 0.45 | 0.033129 | 1.085162 | cluster 1 | High | Low | Low |
| TCGA-DD-A39X-01A | 1 | TCGA-DD-A39X | 1694 | 78 | female | Primary Tumor | G2 | stage i | 48 | 0 - No Fibrosis | WT | 0 | 0 | 0.246052 | 0 | 0 | 0 | 0.753948 | 0.302 | 0.124871 | 1.045289 | cluster 1 | High | Low | Low |
| TCGA-ED-A459-01A | 0 | TCGA-ED-A459 | 910 | 47 | male | Primary Tumor | G2 | stage ii | 51 |  | Mut | 0 | 0 | 0.279407 | 0 | 0.00469 | 0.007402 | 0.708501 | 0.418 | 0.046589 | 1.079557 | cluster 2 | High | Low | Low |
| TCGA-2Y-A9GX-01A | 0 | TCGA-2Y-A9GX | 2442 | 68 | male | Primary Tumor | G2 | stage i | 104 |  | WT | 0 | 0 | 0.361264 | 0 | 0 | 0 | 0.638736 | 0.447 | 0.036792 | 1.077304 | cluster 1 | High | High | Low |
| TCGA-DD-A73E-01A | 0 | TCGA-DD-A73E | 44 | 66 | male | Primary Tumor | G1 | stage i | 108 | 0 - No Fibrosis | WT | 0 | 0 | 0.266837 | 0 | 0 | 0 | 0.733163 | 0.409 | 0.050158 | 1.076907 | cluster 1 | High | Low | Low |
| TCGA-DD-AACL-01A | 1 | TCGA-DD-AACL | 107 | 66 | female | Primary Tumor | G3 | stage i | 69 |  | Mut | 0.066502 | 0 | 0.499355 | 0 | 0.008191 | 0.02659 | 0.399361 | 0.855 | -0.09037 | 1.124814 | cluster 1 | Low | High | High |
| TCGA-MR-A520-01A | 0 | TCGA-MR-A520 | 229 | 58 | male | Primary Tumor | G1 | stage i | 104 |  | WT | 0 | 0 | 0.352594 | 0 | 0 | 0 | 0.647406 | 0.291 | 0.132779 | 1.037343 | cluster 1 | High | High | Low |
| TCGA-DD-A1EF-01A | 1 | TCGA-DD-A1EF | 394 | 57 | female | Primary Tumor | G3 | stage i | 81 | 3,4 - Fibrous Speta | WT | 0 | 0 | 0.296334 | 0 | 0 | 0 | 0.703666 | 0.329 | 0.105185 | 1.051375 | cluster 1 | High | Low | Low |
| TCGA-FV-A4ZP-01A | 1 | TCGA-FV-A4ZP | 2486 | 78 | male | Primary Tumor | G2 | stage iiia | 69 |  | Mut | 0 | 0 | 0.37035 | 0 | 0.087156 | 0 | 0.542494 | 0.251 | 0.166502 | 1.020166 | cluster 2 | High | High | Low |
| TCGA-WX-AA44-01A | 0 | TCGA-WX-AA44 | 615 | 64 | female | Primary Tumor | G3 | stage i | 61 | 6 - Established Cirrhosis | Mut | 0 | 0 | 0.286348 | 0 | 0.055852 | 0 | 0.657799 | 0.285 | 0.137894 | 1.035102 | cluster 2 | High | Low | Low |
| TCGA-ED-A7PX-01A | 0 | TCGA-ED-A7PX | 6 | 48 | female | Primary Tumor | G3 | stage ii | 43 |  | WT | 0.19066 | 0 | 0.282064 | 0 | 0.440283 | 0 | 0.086993 | 0.49 | 0.01702 | 1.096762 | cluster 2 | Low | Low | High |
| TCGA-CC-A7IG-01A | 1 | TCGA-CC-A7IG | 299 | 47 | male | Primary Tumor | G2 | stage ii | 62 |  | Mut | 0 | 0 | 0.368122 | 0 | 0.13927 | 0 | 0.492608 | 0.296 | 0.128646 | 1.036051 | cluster 2 | Low | High | Low |
| TCGA-G3-A25X-01A | 0 | TCGA-G3-A25X | 1779 | 73 | male | Primary Tumor | G3 | stage ii | 65 | 1,2 - Portal Fibrosis | WT | 0.045587 | 0 | 0.549465 | 0 | 0.092228 | 0 | 0.31272 | 0.529 | 0.00107 | 1.082821 | cluster 2 | Low | High | High |
| TCGA-G3-A25U-01A | 0 | TCGA-G3-A25U | 1636 | 63 | female | Primary Tumor | G3 | stage i | 47 | 0 - No Fibrosis | Mut | 0 | 0 | 0.387288 | 0 | 0.010735 | 0 | 0.601977 | 0.451 | 0.032706 | 1.077405 | cluster 2 | High | High | Low |
| TCGA-BC-4072-01B | 1 | TCGA-BC-4072 | 1490 | 74 | female | Primary Tumor | G3 | stage iiia | NA |  | WT | 0.121159 | 0 | 0.537289 | 0 | 0.09535 | 0 | 0.246201 | 0.456 | 0.031085 | 1.067277 | cluster 2 | Low | High | High |
| TCGA-XR-A8TC-01A | 0 | TCGA-XR-A8TC | 1339 | 43 | female | Primary Tumor | G2 | stage i | 52 | 6 - Established Cirrhosis | WT | 0 | 0 | 0.294937 | 0 | 0 | 0.012298 | 0.692764 | 0.296 | 0.128322 | 1.044376 | cluster 1 | High | Low | Low |
| TCGA-BC-A3KF-01A | 0 | TCGA-BC-A3KF | 8 | 66 | female | Primary Tumor | G2 | stage i | 58 |  | WT | 0 | 0 | 0.256591 | 0 | 0 | 0 | 0.743409 | 0.27 | 0.153402 | 1.031868 | cluster 1 | High | Low | Low |
| TCGA-O8-A75V-01A | 0 | TCGA-O8-A75V | 538 | 54 | male | Primary Tumor | G2 | stage i | 92 | 6 - Established Cirrhosis | WT | 0 | 0 | 0.274407 | 0 | 0 | 0.018285 | 0.707308 | 0.339 | 0.099373 | 1.060087 | cluster 1 | High | Low | Low |
| TCGA-BC-A8YO-01A | 0 | TCGA-BC-A8YO | 562 | 66 | female | Primary Tumor | G3 | stage iiic | 74 |  | Mut | 0.029233 | 0 | 0.374357 | 0 | 0.128287 | 0 | 0.468123 | 0.367 | 0.078436 | 1.054344 | cluster 2 | Low | High | High |
| TCGA-DD-A39V-01A | 1 | TCGA-DD-A39V | 643 | 77 | male | Primary Tumor | G3 | stage ii | 79 | 0 - No Fibrosis | WT | 0 | 0 | 0.207615 | 0 | 0 | 0 | 0.792385 | 0.362 | 0.083195 | 1.066567 | cluster 1 | High | Low | Low |
| TCGA-DD-A1EC-01A | 0 | TCGA-DD-A1EC | 602 | 20 | female | Primary Tumor | G3 | stage i | 50 | 0 - No Fibrosis | WT | 0.257147 | 0 | 0.502738 | 0 | 0 | 0.039626 | 0.200489 | 0.744 | -0.06351 | 1.10783 | cluster 1 | Low | High | High |
| TCGA-DD-AACQ-01A | 1 | TCGA-DD-AACQ | 432 | 50 | male | Primary Tumor | G3 | stage ii | 64 |  | Mut | 0 | 0 | 0.273835 | 0 | 0.01175 | 0 | 0.714414 | 0.25 | 0.167077 | 1.024359 | cluster 2 | High | Low | Low |
| TCGA-DD-A1EH-01A | 0 | TCGA-DD-A1EH | 1495 | 23 | male | Primary Tumor | G3 | stage iii | 70 | 3,4 - Fibrous Speta | WT | 0 | 0 | 0.305059 | 0 | 0 | 0 | 0.694941 | 0.245 | 0.176501 | 1.019594 | cluster 1 | High | Low | Low |
| TCGA-KR-A7K7-01A | 0 | TCGA-KR-A7K7 | 951 | 61 | female | Primary Tumor | G1 | stage ii | 125 |  | WT | 0 | 0 | 0.283466 | 0 | 0 | 0 | 0.716534 | 0.403 | 0.054109 | 1.074142 | cluster 1 | High | Low | Low |
| TCGA-DD-AAVV-01A | 0 | TCGA-DD-AAVV | 2455 | 56 | male | Primary Tumor | G3 | stage ii | 71 | 3,4 - Fibrous Speta | Mut | 0 | 0 | 0.393519 | 0 | 0 | 0 | 0.606481 | 0.384 | 0.064873 | 1.064595 | cluster 1 | High | High | Low |
| TCGA-DD-AADN-01A | 0 | TCGA-DD-AADN | 898 | 59 | male | Primary Tumor | G4 | stage i | 62 |  | Mut | 0.673967 | 0 | 0.27673 | 0 | 0 | 0.049303 | 0 | 0.246 | 0.175938 | 1.033489 | cluster 1 | Low | Low | High |
| TCGA-LG-A9QC-01A | 0 | TCGA-LG-A9QC | 425 | 48 | male | Primary Tumor | G2 | stage i | 103 |  | WT | 0 | 0 | 0.265873 | 0 | 0 | 0.019117 | 0.715009 | 0.401 | 0.055403 | 1.080575 | cluster 1 | High | Low | Low |
| TCGA-RC-A7S9-01A | 0 | TCGA-RC-A7S9 | 640 | 47 | female | Primary Tumor | G3 | stage i | 58 | 6 - Established Cirrhosis | WT | 0 | 0 | 0.334546 | 0 | 0.015478 | 0.003363 | 0.646614 | 0.309 | 0.116867 | 1.044863 | cluster 2 | High | High | Low |
| TCGA-BC-A112-01A | 1 | TCGA-BC-A112 | 153 | 80 | male | Primary Tumor | G2 | not reported | 97 | 0 - No Fibrosis | WT | 0 | 0 | 0.520093 | 0 | 0.027309 | 0 | 0.452598 | 0.283 | 0.139237 | 1.033048 | cluster 2 | Low | High | Low |
| TCGA-2Y-A9H0-01A | 0 | TCGA-2Y-A9H0 | 3675 | 49 | male | Primary Tumor | G1 | stage iiia | 89 |  | WT | 0 | 0 | 0.261859 | 0 | 0.076287 | 0 | 0.661854 | 0.341 | 0.098682 | 1.052643 | cluster 2 | High | Low | Low |
| TCGA-DD-AAW2-01A | 0 | TCGA-DD-AAW2 | 1855 | 69 | male | Primary Tumor | G2 | stage i | 50 | 6 - Established Cirrhosis | WT | 0 | 0 | 0.199785 | 0 | 0 | 0 | 0.800215 | 0.33 | 0.104379 | 1.057557 | cluster 1 | High | Low | Low |
| TCGA-KR-A7K2-01A | 0 | TCGA-KR-A7K2 | 829 | 64 | male | Primary Tumor | G1 | stage i | 79 |  | WT | 0 | 0 | 0.297965 | 0 | 0 | 0.002554 | 0.699482 | 0.248 | 0.172714 | 1.022039 | cluster 1 | High | Low | Low |
| TCGA-DD-AADU-01A | 0 | TCGA-DD-AADU | 554 | 60 | male | Primary Tumor | G3 | stage ii | 73 | 3,4 - Fibrous Speta | WT | 0 | 0 | 0.221768 | 0 | 0 | 0 | 0.778232 | 0.368 | 0.077786 | 1.067939 | cluster 1 | High | Low | Low |
| TCGA-ED-A66Y-01A | 1 | TCGA-ED-A66Y | 296 | 51 | female | Primary Tumor | G3 | stage iiia | 59 |  | WT | 0 | 0 | 0.282629 | 0 | 0.2791 | 0 | 0.438271 | 0.338 | 0.09965 | 1.053741 | cluster 2 | Low | Low | Low |
| TCGA-CC-A8HT-01A | 1 | TCGA-CC-A8HT | 140 | 74 | male | Primary Tumor | G2 | stage iiia | 48 |  | Mut | 0 | 0 | 0.408655 | 0 | 0.040849 | 0 | 0.550495 | 0.328 | 0.105919 | 1.045981 | cluster 2 | High | High | Low |
| TCGA-DD-AADY-01A | 0 | TCGA-DD-AADY | 555 | 55 | female | Primary Tumor | G2 | stage i | 55 | 6 - Established Cirrhosis | WT | 0 | 0 | 0.393123 | 0 | 0.035439 | 0 | 0.571439 | 0.35 | 0.092004 | 1.052123 | cluster 2 | High | High | Low |
| TCGA-BC-A216-01A | 0 | TCGA-BC-A216 | 1351 | 62 | female | Primary Tumor | G2 | stage iiia | 151 | 0 - No Fibrosis | WT | 0 | 0 | 0.373918 | 0 | 0.021397 | 0 | 0.604686 | 0.337 | 0.1006 | 1.049487 | cluster 2 | High | High | Low |
| TCGA-G3-AAUZ-01A | 0 | TCGA-G3-AAUZ | 480 | 48 | male | Primary Tumor | G2 | stage i | 77 | 1,2 - Portal Fibrosis | WT | 0 | 0 | 0.207556 | 0 | 0 | 0.013196 | 0.779248 | 0.379 | 0.071176 | 1.075799 | cluster 1 | High | Low | Low |
| TCGA-RC-A7SK-01A | 0 | TCGA-RC-A7SK | 472 | 59 | male | Primary Tumor | G3 | stage i | 60 | 1,2 - Portal Fibrosis | Mut | 0 | 0 | 0.300157 | 0 | 0 | 0 | 0.699843 | 0.288 | 0.135885 | 1.037774 | cluster 1 | High | Low | Low |
| TCGA-G3-A25Z-01A | 0 | TCGA-G3-A25Z | 655 | 58 | male | Primary Tumor | G2 | stage i | 69 | 5 - Nodular Formation and Incomplete Cirrhosis | Mut | 0 | 0 | 0.289269 | 0 | 0 | 0.013826 | 0.696905 | 0.324 | 0.107491 | 1.054379 | cluster 1 | High | Low | Low |
| TCGA-DD-AAD8-01A | 0 | TCGA-DD-AAD8 | 1219 | 73 | female | Primary Tumor | G2 | stage i | 57 | 1,2 - Portal Fibrosis | Mut | 0 | 0 | 0.265947 | 0 | 0 | 0.016951 | 0.717102 | 0.325 | 0.107138 | 1.056654 | cluster 1 | High | Low | Low |
| TCGA-DD-AAEE-01A | 0 | TCGA-DD-AAEE | 810 | 55 | male | Primary Tumor | G4 | stage i | 58 | 6 - Established Cirrhosis | WT | 0 | 0 | 0.220793 | 0 | 0 | 0 | 0.779207 | 0.274 | 0.144378 | 1.037883 | cluster 1 | High | Low | Low |
| TCGA-2Y-A9GU-01A | 0 | TCGA-2Y-A9GU | 1939 | 55 | female | Primary Tumor | G2 | stage i | 78 |  | WT | 0 | 0 | 0.315058 | 0 | 0.026414 | 0 | 0.658527 | 0.305 | 0.121397 | 1.042301 | cluster 2 | High | High | Low |
| TCGA-DD-A119-01A | 1 | TCGA-DD-A119 | 223 | 40 | male | Primary Tumor | G3 | stage iv | 58 | 0 - No Fibrosis | WT | 0 | 0 | 0.296121 | 0 | 0 | 0 | 0.703879 | 0.347 | 0.094729 | 1.055926 | cluster 1 | High | Low | Low |
| TCGA-G3-AAV6-01A | 1 | TCGA-G3-AAV6 | 65 | 53 | female | Primary Tumor | G3 | stage iiia | 78 | 0 - No Fibrosis | WT | 0 | 0 | 0.290902 | 0 | 0.032284 | 0 | 0.676814 | 0.312 | 0.114178 | 1.046151 | cluster 2 | High | Low | Low |
| TCGA-DD-A73F-01A | 0 | TCGA-DD-A73F | 1085 | 77 | female | Primary Tumor | G1 | stage i | 69 | 0 - No Fibrosis | WT | 0 | 0 | 0.31769 | 0 | 0 | 0 | 0.68231 | 0.275 | 0.143726 | 1.03368 | cluster 1 | High | High | Low |
| TCGA-CC-A5UD-01A | 1 | TCGA-CC-A5UD | 304 | 45 | male | Primary Tumor | G2 | stage iiia | 62 |  | WT | 0 | 0 | 0.219755 | 0 | 0.187135 | 0 | 0.59311 | 0.444 | 0.037901 | 1.079972 | cluster 2 | High | Low | Low |
| TCGA-DD-AACD-01A | 1 | TCGA-DD-AACD | 381 | 48 | male | Primary Tumor | G4 | stage i | 66 | 1,2 - Portal Fibrosis | WT | 0 | 0 | 0.309713 | 0 | 0 | 0 | 0.690287 | 0.404 | 0.052844 | 1.073155 | cluster 1 | High | Low | Low |
| TCGA-CC-A8HS-01A | 1 | TCGA-CC-A8HS | 300 | 18 | male | Primary Tumor | G1 | stage iiic | 62 |  | Mut | 0 | 0 | 0.3968 | 0 | 0.163945 | 0 | 0.439255 | 0.309 | 0.117068 | 1.041047 | cluster 2 | Low | High | Low |
| TCGA-DD-A11D-01A | 1 | TCGA-DD-A11D | 1560 | 57 | female | Primary Tumor | G2 | stage i | 73 | 6 - Established Cirrhosis | WT | 0 | 0 | 0.30012 | 0 | 0 | 0.011103 | 0.688777 | 0.277 | 0.141702 | 1.037849 | cluster 1 | High | Low | Low |
| TCGA-DD-A1EG-01A | 1 | TCGA-DD-A1EG | 1372 | 76 | male | Primary Tumor | G3 | stage i | 82 | 1,2 - Portal Fibrosis | WT | 0 | 0 | 0.380512 | 0 | 0 | 0 | 0.619488 | 0.416 | 0.047763 | 1.07203 | cluster 1 | High | High | Low |
| TCGA-PD-A5DF-01A | 1 | TCGA-PD-A5DF | 639 | 58 | female | Primary Tumor | G2 | stage iiib | 72 |  | WT | 0 | 0 | 0.330741 | 0 | 0.007295 | 0 | 0.661964 | 0.372 | 0.072877 | 1.06325 | cluster 2 | High | High | Low |
| TCGA-DD-A3A9-01A | 1 | TCGA-DD-A3A9 | 931 | 64 | female | Primary Tumor | G2 | stage ivb | 62 | 0 - No Fibrosis | WT | 0 | 0 | 0.357844 | 0 | 0 | 0.002769 | 0.639387 | 0.247 | 0.174623 | 1.019708 | cluster 1 | High | High | Low |
| TCGA-2Y-A9H4-01A | 0 | TCGA-2Y-A9H4 | 1452 | 68 | male | Primary Tumor | G2 | stage i | 61 |  | Mut | 0 | 0 | 0.31461 | 0 | 0 | 0 | 0.68539 | 0.322 | 0.108358 | 1.049177 | cluster 1 | High | High | Low |
| TCGA-DD-AACK-01A | 0 | TCGA-DD-AACK | 9 | 70 | male | Primary Tumor | G2 | stage i | 68 | 6 - Established Cirrhosis | WT | 0 | 0 | 0.320854 | 0 | 0 | 0 | 0.679146 | 0.351 | 0.090206 | 1.056707 | cluster 1 | High | High | Low |
| TCGA-DD-A118-01A | 0 | TCGA-DD-A118 | 3437 | 77 | female | Primary Tumor | G2 | stage ii | 75 | 0 - No Fibrosis | WT | 0 | 0 | 0.37922 | 0 | 0.072303 | 0 | 0.548477 | 0.35 | 0.091122 | 1.051891 | cluster 2 | High | High | Low |
| TCGA-DD-A4NL-01A | 0 | TCGA-DD-A4NL | 1711 | 46 | male | Primary Tumor | G1 | stage i | 113 | 0 - No Fibrosis | WT | 0 | 0 | 0.324647 | 0 | 0 | 0 | 0.675353 | 0.38 | 0.068738 | 1.065663 | cluster 1 | High | High | Low |
| TCGA-5C-A9VH-01A | 0 | TCGA-5C-A9VH | 322 | 70 | male | Primary Tumor | G2 | stage i | 69 |  | WT | 0 | 0 | 0.299367 | 0 | 0 | 0 | 0.700633 | 0.241 | 0.179443 | 1.018452 | cluster 1 | High | Low | Low |
| TCGA-3K-AAZ8-01A | 0 | TCGA-3K-AAZ8 | 396 | 65 | male | Primary Tumor | G1 | stage iiib | 84 | 5 - Nodular Formation and Incomplete Cirrhosis | WT | 0 | 0 | 0.354099 | 0 | 0 | 0 | 0.645901 | 0.309 | 0.116279 | 1.044344 | cluster 1 | High | High | Low |
| TCGA-MI-A75G-01A | 0 | TCGA-MI-A75G | 698 | 63 | male | Primary Tumor | G2 | stage ii | 69 | 6 - Established Cirrhosis | Mut | 0 | 0 | 0.244486 | 0 | 0 | 0 | 0.755514 | 0.271 | 0.150828 | 1.033634 | cluster 1 | High | Low | Low |
| TCGA-DD-A3A6-01A | 1 | TCGA-DD-A3A6 | 3258 | 72 | female | Primary Tumor | G2 | stage ii | 52 | 0 - No Fibrosis | WT | 0.296199 | 0 | 0.53503 | 0.147562 | 0 | 0.021209 | 0 | 0.132 | 0.32692 | 0.960656 | cluster 1 | Low | High | High |
| TCGA-DD-AADM-01A | 1 | TCGA-DD-AADM | 12 | 58 | male | Primary Tumor | G3 | stage ii | 55 |  | WT | 0 | 0 | 0.202191 | 0 | 0 | 0.012858 | 0.78495 | 0.368 | 0.077405 | 1.073271 | cluster 1 | High | Low | Low |
| TCGA-DD-AAC8-01A | 1 | TCGA-DD-AAC8 | 16 | 72 | male | Primary Tumor | G3 | stage i | 57 | 1,2 - Portal Fibrosis | WT | 0 | 0 | 0.266292 | 0 | 0 | 0 | 0.733708 | 0.484 | 0.019307 | 1.090179 | cluster 1 | High | Low | Low |
| TCGA-DD-A4NN-01A | 1 | TCGA-DD-A4NN | 899 | 56 | female | Primary Tumor | G3 | stage i | 59 | 0 - No Fibrosis | WT | 0 | 0 | 0.300911 | 0 | 0 | 0 | 0.699089 | 0.228 | 0.191877 | 1.012816 | cluster 1 | High | Low | Low |
| TCGA-DD-A4NH-01A | 0 | TCGA-DD-A4NH | 917 | 65 | female | Primary Tumor | G3 | stage iiib | 81 | 1,2 - Portal Fibrosis | WT | 0 | 0 | 0.46879 | 0 | 0 | 0 | 0.53121 | 0.271 | 0.149595 | 1.028974 | cluster 1 | High | High | Low |
| TCGA-K7-A6G5-01A | 0 | TCGA-K7-A6G5 | 512 | 66 | male | Primary Tumor | G2 | stage i | 80 |  | WT | 0 | 0 | 0.313183 | 0 | 0 | 0.009935 | 0.676882 | 0.362 | 0.083032 | 1.062885 | cluster 1 | High | High | Low |
| TCGA-DD-AACU-01A | 0 | TCGA-DD-AACU | 1567 | 59 | male | Primary Tumor | G3 | stage i | 67 |  | Mut | 0 | 0 | 0.207212 | 0 | 0.003009 | 0.009497 | 0.780281 | 0.242 | 0.178317 | 1.024807 | cluster 2 | High | Low | Low |
| TCGA-CC-A7IL-01A | 1 | TCGA-CC-A7IL | 278 | 61 | male | Primary Tumor | G1 | stage iiia | 63 |  | WT | 0 | 0 | 0.198857 | 0 | 0 | 0 | 0.801143 | 0.281 | 0.139366 | 1.041554 | cluster 1 | High | Low | Low |
| TCGA-BW-A5NO-01A | 0 | TCGA-BW-A5NO | 20 | 50 | male | Primary Tumor | G2 | stage iiia | NA | 3,4 - Fibrous Speta | Mut | 0.031816 | 0 | 0.276174 | 0 | 0.072504 | 0 | 0.619505 | 0.422 | 0.044101 | 1.072063 | cluster 2 | High | Low | High |
| TCGA-XR-A8TG-01A | 0 | TCGA-XR-A8TG | 898 | 58 | male | Primary Tumor | G2 | stage i | 71 | 6 - Established Cirrhosis | Mut | 0 | 0 | 0.260804 | 0 | 0.017913 | 0.017499 | 0.703784 | 0.423 | 0.043356 | 1.084718 | cluster 2 | High | Low | Low |
| TCGA-DD-A1EJ-01A | 1 | TCGA-DD-A1EJ | 1005 | 71 | female | Primary Tumor | G2 | stage iiic | 64 | 0 - No Fibrosis | WT | 0 | 0 | 0.308739 | 0 | 0 | 0.010543 | 0.680717 | 0.253 | 0.165234 | 1.026804 | cluster 1 | High | Low | Low |
| TCGA-G3-A7M8-01A | 0 | TCGA-G3-A7M8 | 430 | 31 | male | Primary Tumor | G1 | stage i | NA | 6 - Established Cirrhosis | WT | 0 | 0 | 0.341475 | 0 | 0 | 0 | 0.658525 | 0.297 | 0.127131 | 1.040096 | cluster 1 | High | High | Low |
| TCGA-UB-AA0V-01A | 0 | TCGA-UB-AA0V | 314 | 69 | female | Primary Tumor | G1 | stage i | 57 | 0 - No Fibrosis | WT | 0 | 0 | 0.349073 | 0 | 0 | 0 | 0.650927 | 0.354 | 0.088689 | 1.056199 | cluster 1 | High | High | Low |
| TCGA-DD-AACZ-01A | 1 | TCGA-DD-AACZ | 171 | 63 | female | Primary Tumor | G4 | stage i | 52 |  | Mut | 0 | 0 | 0.389952 | 0 | 0.067165 | 0 | 0.542883 | 0.449 | 0.034419 | 1.074828 | cluster 2 | High | High | Low |
| TCGA-DD-A1EB-01A | 0 | TCGA-DD-A1EB | 2017 | 72 | female | Primary Tumor | G2 | stage i | 57 | 0 - No Fibrosis | Mut | 0 | 0 | 0.391218 | 0 | 0 | 0 | 0.608782 | 0.313 | 0.11386 | 1.044399 | cluster 1 | High | High | Low |
| TCGA-BC-4073-01B | 0 | TCGA-BC-4073 | 849 | 73 | male | Primary Tumor | G3 | stage iiia | NA |  | WT | 0.151871 | 0 | 0.539461 | 0 | 0.018049 | 0 | 0.290619 | 0.545 | -0.00458 | 1.077896 | cluster 2 | Low | High | High |
| TCGA-ZS-A9CG-01A | 0 | TCGA-ZS-A9CG | 341 | 55 | male | Primary Tumor | G2 | stage ii | 94 | 0 - No Fibrosis | WT | 0 | 0 | 0.335792 | 0 | 0 | 0 | 0.664208 | 0.421 | 0.04494 | 1.075119 | cluster 1 | High | High | Low |
| TCGA-BD-A2L6-01A | 0 | TCGA-BD-A2L6 | 1363 | 69 | male | Primary Tumor | G2 | not reported | 110 | 3,4 - Fibrous Speta | WT | 0 | 0 | 0.184007 | 0 | 0.078214 | 0.005656 | 0.732123 | 0.26 | 0.161847 | 1.0297 | cluster 2 | High | Low | Low |
| TCGA-CC-5260-01A | 1 | TCGA-CC-5260 | 87 | 61 | female | Primary Tumor | G1 | stage iiic | 46 |  | WT | 0.324812 | 0 | 0.355885 | 0 | 0.319303 | 0 | 0 | 0.328 | 0.106005 | 1.047744 | cluster 2 | Low | High | High |
| TCGA-DD-AAD5-01A | 0 | TCGA-DD-AAD5 | 1345 | 54 | male | Primary Tumor | G3 | stage i | 70 | 3,4 - Fibrous Speta | Mut | 0 | 0 | 0.552132 | 0 | 0.081178 | 0 | 0.36669 | 0.533 | -0.00101 | 1.087465 | cluster 2 | Low | High | Low |
| TCGA-BD-A3EP-01A | 0 | TCGA-BD-A3EP | 409 | 75 | female | Primary Tumor | G2 | stage i | 71 | 1,2 - Portal Fibrosis | Mut | 0.120701 | 0 | 0.383408 | 0 | 0.004961 | 0 | 0.490929 | 0.582 | -0.01797 | 1.086413 | cluster 2 | Low | High | High |
| TCGA-WQ-A9G7-01A | 0 | TCGA-WQ-A9G7 | 30 | 71 | female | Primary Tumor | G3 | not reported | 69 |  | WT | 0 | 0 | 0.35017 | 0 | 0.04571 | 0 | 0.60412 | 0.35 | 0.09128 | 1.053218 | cluster 2 | High | High | Low |
| TCGA-FV-A2QR-01A | 1 | TCGA-FV-A2QR | 581 | 75 | male | Primary Tumor | G1 | stage i | NA |  | WT | 0 | 0 | 0.405201 | 0 | 0.046474 | 0 | 0.548326 | 0.337 | 0.101241 | 1.047811 | cluster 2 | High | High | Low |
| TCGA-DD-AAC9-01A | 0 | TCGA-DD-AAC9 | 347 | 51 | male | Primary Tumor | G2 | stage i | 81 | 6 - Established Cirrhosis | WT | 0 | 0 | 0.315721 | 0 | 0 | 0 | 0.684279 | 0.392 | 0.059494 | 1.070019 | cluster 1 | High | High | Low |
| TCGA-DD-AA3A-01A | 1 | TCGA-DD-AA3A | 410 | 81 | female | Primary Tumor | G4 | stage i | 60 | 0 - No Fibrosis | WT | 0.084547 | 0.126155 | 0.161902 | 0 | 0 | 0 | 0.627396 | 0.227 | 0.194368 | 1.013133 | cluster 1 | High | Low | High |
| TCGA-CC-5263-01A | 1 | TCGA-CC-5263 | 129 | 35 | male | Primary Tumor | G1 | stage iiia | 59 |  | Mut | 0 | 0 | 0.364239 | 0 | 0.358074 | 0 | 0.277687 | 0.427 | 0.042774 | 1.08416 | cluster 2 | Low | High | Low |
| TCGA-FV-A495-01A | 0 | TCGA-FV-A495 | 1 | 51 | female | Primary Tumor | G2 | stage ii | 70 | 3,4 - Fibrous Speta | WT | 0 | 0 | 0.278885 | 0 | 0 | 0.003629 | 0.717486 | 0.276 | 0.14195 | 1.036827 | cluster 1 | High | Low | Low |
| TCGA-BC-A10Z-01A | 1 | TCGA-BC-A10Z | 34 | 62 | female | Primary Tumor | G2 | stage i | 79 |  | WT | 0 | 0 | 0.337681 | 0 | 0.002842 | 0 | 0.659477 | 0.366 | 0.080649 | 1.059898 | cluster 2 | High | High | Low |
| TCGA-DD-AAVR-01A | 0 | TCGA-DD-AAVR | 2513 | 44 | male | Primary Tumor | G2 | stage i | 69 | 6 - Established Cirrhosis | WT | 0 | 0 | 0.282302 | 0 | 0 | 0.012752 | 0.704947 | 0.343 | 0.096605 | 1.05929 | cluster 1 | High | Low | Low |
| TCGA-ZS-A9CE-01A | 0 | TCGA-ZS-A9CE | 1241 | 79 | female | Primary Tumor | G1 | stage ii | NA | 0 - No Fibrosis | WT | 0 | 0 | 0.202214 | 0 | 0 | 0.009093 | 0.788693 | 0.334 | 0.102733 | 1.060553 | cluster 1 | High | Low | Low |
| TCGA-CC-5259-01A | 0 | TCGA-CC-5259 | 250 | 60 | female | Primary Tumor | G2 | stage iiic | 49 |  | Mut | 0 | 0 | 0.313416 | 0 | 0 | 0.008517 | 0.678067 | 0.287 | 0.136191 | 1.039154 | cluster 1 | High | High | Low |
| TCGA-5R-AA1D-01A | 0 | TCGA-5R-AA1D | 449 | 17 | female | Primary Tumor | G3 | stage iiia | 59 | 0 - No Fibrosis | WT | 0 | 0 | 0.252528 | 0 | 0 | 0.022994 | 0.724478 | 0.518 | 0.005179 | 1.10516 | cluster 1 | High | Low | Low |
| TCGA-ED-A82E-01A | 0 | TCGA-ED-A82E | 408 | 60 | female | Primary Tumor | G2 | stage iiia | 44 |  | WT | 0.328218 | 0.080623 | 0 | 0 | 0.414539 | 0 | 0.17662 | 0.238 | 0.182544 | 1.026982 | cluster 2 | Low | Low | High |
| TCGA-DD-A3A1-01A | 1 | TCGA-DD-A3A1 | 233 | 65 | male | Primary Tumor | G2 | stage iiia | 78 | 0 - No Fibrosis | WT | 0 | 0 | 0.295516 | 0 | 0 | 0.005996 | 0.698489 | 0.35 | 0.091363 | 1.05902 | cluster 1 | High | Low | Low |
| TCGA-DD-AAEA-01A | 0 | TCGA-DD-AAEA | 575 | 65 | male | Primary Tumor | G3 | stage i | 66 | 1,2 - Portal Fibrosis | Mut | 0 | 0 | 0.270239 | 0 | 0 | 0 | 0.729761 | 0.252 | 0.165541 | 1.025768 | cluster 1 | High | Low | Low |
| TCGA-ZS-A9CF-01A | 0 | TCGA-ZS-A9CF | 2412 | 64 | male | Primary Tumor | G2 | stage ii | NA | 0 - No Fibrosis | WT | 0 | 0 | 0.265974 | 0 | 0 | 0.013236 | 0.72079 | 0.354 | 0.088727 | 1.063868 | cluster 1 | High | Low | Low |
| TCGA-G3-A25V-01A | 0 | TCGA-G3-A25V | 860 | 68 | male | Primary Tumor | G2 | stage i | 90 | 6 - Established Cirrhosis | Mut | 0 | 0 | 0.328165 | 0 | 0 | 0 | 0.671835 | 0.367 | 0.07927 | 1.061039 | cluster 1 | High | High | Low |
| TCGA-CC-A9FS-01A | 0 | TCGA-CC-A9FS | 211 | 55 | male | Primary Tumor | G2 | stage ii | 72 |  | WT | 0 | 0 | 0.198761 | 0 | 0.000541 | 0.008762 | 0.791937 | 0.249 | 0.17114 | 1.028664 | cluster 2 | High | Low | Low |
| TCGA-BC-A69H-01A | 0 | TCGA-BC-A69H | 444 | 64 | male | Primary Tumor | G3 | stage ii | 70 |  | Mut | 0 | 0 | 0.368365 | 0 | 0.020601 | 0 | 0.611034 | 0.249 | 0.170759 | 1.019894 | cluster 2 | High | High | Low |
| TCGA-DD-A3A7-01A | 1 | TCGA-DD-A3A7 | 419 | 67 | male | Primary Tumor | G3 | stage iiib | 80 | 0 - No Fibrosis | Mut | 0 | 0 | 0.269815 | 0 | 0 | 0 | 0.730185 | 0.246 | 0.17593 | 1.021076 | cluster 1 | High | Low | Low |
| TCGA-G3-A7M6-01A | 0 | TCGA-G3-A7M6 | 632 | 60 | female | Primary Tumor | G3 | stage i | 77 | 1,2 - Portal Fibrosis | Mut | 0 | 0 | 0.454695 | 0 | 0.072386 | 0 | 0.47292 | 0.333 | 0.102906 | 1.046371 | cluster 2 | Low | High | Low |
| TCGA-MI-A75E-01A | 0 | TCGA-MI-A75E | 507 | 61 | male | Primary Tumor | G2 | stage iiic | 103 | 1,2 - Portal Fibrosis | WT | 0 | 0 | 0.226947 | 0 | 0 | 0 | 0.773053 | 0.363 | 0.082667 | 1.065392 | cluster 1 | High | Low | Low |
| TCGA-DD-AAE4-01A | 0 | TCGA-DD-AAE4 | 608 | 49 | female | Primary Tumor | G1 | stage i | 62 | 6 - Established Cirrhosis | WT | 0.00545 | 0 | 0.334247 | 0 | 0 | 0 | 0.660303 | 0.483 | 0.020113 | 1.084866 | cluster 1 | High | High | High |
| TCGA-CC-A7II-01A | 0 | TCGA-CC-A7II | 399 | 54 | male | Primary Tumor | G3 | stage iiia | 52 |  | Mut | 0 | 0 | 0.422982 | 0 | 0.478342 | 0 | 0.098675 | 0.329 | 0.105516 | 1.072036 | cluster 2 | Low | High | Low |
| TCGA-G3-A7M5-01A | 0 | TCGA-G3-A7M5 | 447 | 76 | male | Primary Tumor | G2 | stage i | 68 | 0 - No Fibrosis | WT | 0 | 0 | 0.289973 | 0 | 0 | 0.000637 | 0.70939 | 0.265 | 0.156756 | 1.029065 | cluster 1 | High | Low | Low |
| TCGA-CC-A3MC-01A | 0 | TCGA-CC-A3MC | 363 | 54 | male | Primary Tumor | G2 | stage iiia | 52 |  | WT | 0 | 0 | 0.281696 | 0 | 0 | 0.013264 | 0.70504 | 0.303 | 0.122987 | 1.047642 | cluster 1 | High | Low | Low |
| TCGA-ES-A2HT-01A | 1 | TCGA-ES-A2HT | 438 | 54 | male | Primary Tumor | G2 | stage i | 80 | 0 - No Fibrosis | WT | 0 | 0 | 0.275661 | 0 | 0 | 0.002741 | 0.721598 | 0.384 | 0.064494 | 1.070925 | cluster 1 | High | Low | Low |
| TCGA-G3-A6UC-01A | 0 | TCGA-G3-A6UC | 671 | 65 | male | Primary Tumor | G2 | stage iiib | 91 | 6 - Established Cirrhosis | WT | 0 | 0 | 0.274531 | 0 | 0 | 0 | 0.725469 | 0.349 | 0.09267 | 1.057966 | cluster 1 | High | Low | Low |
| TCGA-BD-A3ER-01A | 0 | TCGA-BD-A3ER | 1115 | 62 | male | Primary Tumor | G2 | stage ii | 111 | 6 - Established Cirrhosis | WT | 0 | 0 | 0.295449 | 0 | 0 | 0.014002 | 0.690549 | 0.387 | 0.063218 | 1.073676 | cluster 1 | High | Low | Low |
| TCGA-DD-A3A5-01A | 1 | TCGA-DD-A3A5 | 3125 | 66 | female | Primary Tumor | G2 | stage iii | 58 | 0 - No Fibrosis | WT | 0 | 0 | 0.310639 | 0 | 0 | 0.006159 | 0.683202 | 0.272 | 0.147415 | 1.033706 | cluster 1 | High | Low | Low |
| TCGA-DD-AACH-01A | 1 | TCGA-DD-AACH | 195 | 69 | male | Primary Tumor | G3 | stage ii | 53 | 6 - Established Cirrhosis | WT | 0 | 0 | 0.300543 | 0 | 0.051857 | 0 | 0.647599 | 0.3 | 0.126169 | 1.039824 | cluster 2 | High | Low | Low |
| TCGA-CC-5262-01A | 1 | TCGA-CC-5262 | 103 | 67 | male | Primary Tumor | G1 | stage iiic | 62 |  | WT | 0 | 0 | 0.285608 | 0 | 0.00279 | 0 | 0.711602 | 0.38 | 0.06881 | 1.067529 | cluster 2 | High | Low | Low |
| TCGA-DD-AADG-01A | 0 | TCGA-DD-AADG | 1145 | 70 | male | Primary Tumor | G3 | stage iiia | 61 | 3,4 - Fibrous Speta | WT | 0 | 0 | 0.241602 | 0 | 0 | 0 | 0.758398 | 0.342 | 0.097271 | 1.057904 | cluster 1 | High | Low | Low |
| TCGA-DD-A39Z-01A | 1 | TCGA-DD-A39Z | 601 | 43 | female | Primary Tumor | G2 | stage ii | 61 |  | WT | 0 | 0 | 0.299134 | 0 | 0 | 0.004614 | 0.696252 | 0.279 | 0.1403 | 1.036939 | cluster 1 | High | Low | Low |
| TCGA-DD-AACJ-01A | 0 | TCGA-DD-AACJ | 2102 | 75 | male | Primary Tumor | G2 | stage ii | 73 |  | WT | 0 | 0 | 0.280133 | 0 | 0 | 0 | 0.719867 | 0.313 | 0.113102 | 1.048704 | cluster 1 | High | Low | Low |
| TCGA-DD-AADL-01A | 0 | TCGA-DD-AADL | 636 | 58 | male | Primary Tumor | G4 | stage i | 53 |  | Mut | 0 | 0 | 0.350173 | 0 | 0 | 0 | 0.649827 | 0.351 | 0.091036 | 1.055169 | cluster 1 | High | High | Low |
| TCGA-DD-AAEG-01A | 0 | TCGA-DD-AAEG | 719 | 59 | female | Primary Tumor | G3 | stage i | 40 | 6 - Established Cirrhosis | WT | 0 | 0 | 0.316111 | 0 | 0 | 0 | 0.683889 | 0.368 | 0.076796 | 1.062651 | cluster 1 | High | High | Low |
| TCGA-DD-A4NO-01A | 0 | TCGA-DD-A4NO | 2245 | 65 | male | Primary Tumor | G1 | stage i | 109 | 0 - No Fibrosis | WT | 0 | 0 | 0.19961 | 0 | 0 | 0.011419 | 0.788971 | 0.278 | 0.140628 | 1.043717 | cluster 1 | High | Low | Low |
| TCGA-CC-A5UE-01A | 1 | TCGA-CC-A5UE | 272 | 48 | male | Primary Tumor | G2 | stage iiib | 65 |  | WT | 0 | 0 | 0.286763 | 0 | 0.02872 | 0 | 0.684517 | 0.318 | 0.110829 | 1.047946 | cluster 2 | High | Low | Low |
| TCGA-G3-A7M9-01A | 1 | TCGA-G3-A7M9 | 56 | 70 | male | Primary Tumor | G2 | stage iiib | 73 | 6 - Established Cirrhosis | Mut | 0 | 0 | 0.402457 | 0 | 0.160404 | 0 | 0.437139 | 0.314 | 0.111664 | 1.043223 | cluster 2 | Low | High | Low |
| TCGA-2Y-A9HB-01A | 0 | TCGA-2Y-A9HB | 260 | 66 | male | Primary Tumor | G2 | stage i | 74 |  | WT | 0 | 0 | 0.295343 | 0 | 0 | 0.011911 | 0.692746 | 0.379 | 0.071216 | 1.069542 | cluster 1 | High | Low | Low |
| TCGA-DD-A39W-01A | 1 | TCGA-DD-A39W | 827 | 29 | female | Primary Tumor | G2 | stage iii | 74 |  | WT | 0 | 0 | 0.33158 | 0 | 0.02034 | 0 | 0.64808 | 0.477 | 0.024107 | 1.082912 | cluster 2 | High | High | Low |
| TCGA-2Y-A9GV-01A | 1 | TCGA-2Y-A9GV | 2532 | 54 | female | Primary Tumor | G1 | stage i | 85 |  | WT | 0 | 0 | 0.319003 | 0 | 0 | 0.007474 | 0.673523 | 0.339 | 0.099296 | 1.054859 | cluster 1 | High | High | Low |
| TCGA-DD-AACF-01A | 1 | TCGA-DD-AACF | 365 | 68 | male | Primary Tumor | G3 | stage i | 60 | 6 - Established Cirrhosis | Mut | 0 | 0 | 0.357371 | 0 | 0 | 0 | 0.642629 | 0.347 | 0.094368 | 1.053511 | cluster 1 | High | High | Low |
| TCGA-UB-A7MA-01A | 0 | TCGA-UB-A7MA | 848 | 62 | female | Primary Tumor | G2 | stage ii | 85 | 0 - No Fibrosis | WT | 0 | 0 | 0.39005 | 0 | 0.052526 | 0 | 0.557424 | 0.287 | 0.136425 | 1.033284 | cluster 2 | High | High | Low |
| TCGA-DD-AAE7-01A | 0 | TCGA-DD-AAE7 | 644 | 72 | male | Primary Tumor | G2 | stage i | 74 | 3,4 - Fibrous Speta | WT | 0 | 0 | 0.344983 | 0 | 0 | 0 | 0.655017 | 0.322 | 0.108961 | 1.047755 | cluster 1 | High | High | Low |
| TCGA-MI-A75C-01A | 0 | TCGA-MI-A75C | 291 | 64 | male | Primary Tumor | G3 | stage i | 84 |  | WT | 0 | 0 | 0.303574 | 0 | 0 | 0.010619 | 0.685807 | 0.383 | 0.066508 | 1.070756 | cluster 1 | High | Low | Low |
| TCGA-DD-A4NA-01A | 0 | TCGA-DD-A4NA | 1008 | 67 | female | Primary Tumor | G3 | stage iiic | 93 | 5 - Nodular Formation and Incomplete Cirrhosis | WT | 0.381034 | 0 | 0.519171 | 0 | 0.084363 | 0.015431 | 0 | 0.548 | -0.00701 | 1.085631 | cluster 2 | Low | High | High |
| TCGA-ZP-A9CV-01A | 1 | TCGA-ZP-A9CV | 1088 | 59 | male | Primary Tumor | G1 | not reported | 59 |  | WT | 0 | 0 | 0.330871 | 0 | 0 | 0 | 0.669129 | 0.358 | 0.084944 | 1.058513 | cluster 1 | High | High | Low |
| TCGA-RC-A6M6-01A | 0 | TCGA-RC-A6M6 | 9 | 75 | male | Primary Tumor | G3 | stage ii | NA |  | WT | 0 | 0 | 0.338782 | 0 | 0 | 0.002965 | 0.658253 | 0.33 | 0.10454 | 1.050612 | cluster 1 | High | High | Low |
| TCGA-T1-A6J8-01A | 0 | TCGA-T1-A6J8 | 23 | 68 | male | Primary Tumor | G2 | not reported | 65 |  | Mut | 0 | 0 | 0.192727 | 0 | 0 | 0.009808 | 0.797466 | 0.244 | 0.177067 | 1.026464 | cluster 1 | High | Low | Low |
| TCGA-DD-A1EK-01A | 1 | TCGA-DD-A1EK | 558 | 64 | female | Primary Tumor | G2 | stage ivb | 97 | 0 - No Fibrosis | WT | 0 | 0 | 0.296248 | 0 | 0 | 0.013212 | 0.69054 | 0.301 | 0.125877 | 1.045655 | cluster 1 | High | Low | Low |
| TCGA-KR-A7K8-01A | 0 | TCGA-KR-A7K8 | 906 | 57 | male | Primary Tumor | G1 | stage i | 109 |  | WT | 0.140203 | 0 | 0.276558 | 0 | 0.034574 | 0 | 0.548665 | 0.474 | 0.025125 | 1.072215 | cluster 2 | High | Low | High |
| TCGA-DD-AAD6-01A | 0 | TCGA-DD-AAD6 | 672 | 66 | male | Primary Tumor | G3 | stage iiia | 93 |  | WT | 0 | 0 | 0.299884 | 0 | 0 | 0.011786 | 0.68833 | 0.393 | 0.05879 | 1.074668 | cluster 1 | High | Low | Low |
| TCGA-DD-A4NB-01A | 0 | TCGA-DD-A4NB | 989 | 25 | male | Primary Tumor | G2 | stage i | 69 |  | WT | 0 | 0 | 0.27546 | 0 | 0 | 0.010875 | 0.713665 | 0.484 | 0.019305 | 1.093015 | cluster 1 | High | Low | Low |
| TCGA-DD-A11C-01A | 0 | TCGA-DD-A11C | 662 | 69 | male | Primary Tumor | G3 | stage i | 123 | 0 - No Fibrosis | WT | 0 | 0 | 0.311832 | 0 | 0.003842 | 0 | 0.684326 | 0.401 | 0.055307 | 1.071779 | cluster 2 | High | Low | Low |
| TCGA-RC-A7SF-01A | 0 | TCGA-RC-A7SF | 579 | 66 | male | Primary Tumor | G2 | stage i | 76 | 3,4 - Fibrous Speta | WT | 0 | 0 | 0.308327 | 0 | 0 | 0.010565 | 0.681108 | 0.313 | 0.113248 | 1.050041 | cluster 1 | High | Low | Low |
| TCGA-ED-A8O6-01A | 1 | TCGA-ED-A8O6 | 56 | 50 | female | Primary Tumor | G3 | stage iiia | 45 |  | WT | 0 | 0 | 0.280132 | 0 | 0 | 0.017268 | 0.7026 | 0.307 | 0.120457 | 1.049956 | cluster 1 | High | Low | Low |
| TCGA-DD-AAEB-01A | 0 | TCGA-DD-AAEB | 478 | 60 | male | Primary Tumor | G2 | stage i | 75 |  | WT | 0 | 0 | 0.11532 | 0 | 0 | 0 | 0.88468 | 0.309 | 0.117831 | 1.05824 | cluster 1 | High | Low | Low |
| TCGA-DD-AAVP-01A | 0 | TCGA-DD-AAVP | 2752 | 48 | male | Primary Tumor | G1 | stage i | 62 | 6 - Established Cirrhosis | WT | 0 | 0 | 0.318837 | 0 | 0 | 0 | 0.681163 | 0.474 | 0.025107 | 1.084297 | cluster 1 | High | High | Low |
| TCGA-DD-A73A-01A | 0 | TCGA-DD-A73A | 728 | 71 | male | Primary Tumor | G2 | stage i | 102 | 3,4 - Fibrous Speta | WT | 0 | 0 | 0.326362 | 0 | 0 | 0 | 0.673638 | 0.349 | 0.093326 | 1.055136 | cluster 1 | High | High | Low |
| TCGA-RC-A6M4-01A | 0 | TCGA-RC-A6M4 | 22 | 74 | female | Primary Tumor | G2 | stage iiia | 87 |  | WT | 0 | 0 | 0.182199 | 0 | 0 | 0 | 0.817801 | 0.303 | 0.123373 | 1.050115 | cluster 1 | High | Low | Low |
| TCGA-G3-AAV0-01A | 0 | TCGA-G3-AAV0 | 476 | 58 | male | Primary Tumor | G2 | stage i | 58 | 0 - No Fibrosis | WT | 0 | 0 | 0.342943 | 0 | 0 | 0.002121 | 0.654936 | 0.271 | 0.152155 | 1.029706 | cluster 1 | High | High | Low |
| TCGA-DD-AAVY-01A | 0 | TCGA-DD-AAVY | 1970 | 56 | male | Primary Tumor | G2 | stage iiia | 63 | 0 - No Fibrosis | WT | 0 | 0 | 0.361352 | 0 | 0 | 0 | 0.638648 | 0.465 | 0.027654 | 1.081049 | cluster 1 | High | High | Low |
| TCGA-DD-A1EL-01A | 1 | TCGA-DD-A1EL | 415 | 23 | male | Primary Tumor | G3 | stage ii | 54 | 0 - No Fibrosis | Mut | 0 | 0 | 0.12403 | 0 | 0 | 0 | 0.87597 | 0.379 | 0.070997 | 1.079485 | cluster 1 | High | Low | Low |
| TCGA-DD-A4NJ-01A | 0 | TCGA-DD-A4NJ | 928 | 54 | female | Primary Tumor | G2 | stage ii | 88 | 1,2 - Portal Fibrosis | WT | 0 | 0 | 0.323139 | 0 | 0.017295 | 0 | 0.659566 | 0.398 | 0.056667 | 1.069927 | cluster 2 | High | High | Low |
| TCGA-G3-A5SM-01A | 0 | TCGA-G3-A5SM | 520 | 58 | male | Primary Tumor | G3 | stage ii | 106 | 6 - Established Cirrhosis | Mut | 0 | 0 | 0.282591 | 0 | 0 | 0 | 0.717409 | 0.324 | 0.107495 | 1.051043 | cluster 1 | High | Low | Low |
| TCGA-G3-A5SL-01A | 0 | TCGA-G3-A5SL | 621 | 70 | male | Primary Tumor | G2 | stage ii | 88 | 0 - No Fibrosis | WT | 0 | 0 | 0.171506 | 0 | 0 | 0 | 0.828494 | 0.35 | 0.092252 | 1.06531 | cluster 1 | High | Low | Low |
| TCGA-BC-A10U-01A | 1 | TCGA-BC-A10U | 837 | 69 | male | Primary Tumor | G2 | not reported | NA |  | Mut | 0 | 0 | 0.285818 | 0 | 0 | 0.014952 | 0.69923 | 0.398 | 0.056626 | 1.077428 | cluster 1 | High | Low | Low |
| TCGA-DD-AACG-01A | 1 | TCGA-DD-AACG | 469 | 52 | male | Primary Tumor | G4 | stage ii | 65 | 6 - Established Cirrhosis | Mut | 0 | 0 | 0.317114 | 0 | 0 | 0.007781 | 0.675106 | 0.284 | 0.138633 | 1.037756 | cluster 1 | High | High | Low |
| TCGA-DD-A3A3-01A | 1 | TCGA-DD-A3A3 | 535 | 45 | male | Primary Tumor | G2 | stage i | 71 | 0 - No Fibrosis | WT | 0 | 0 | 0.302737 | 0 | 0 | 0 | 0.697263 | 0.36 | 0.083786 | 1.060318 | cluster 1 | High | Low | Low |
| TCGA-ED-A7XO-01A | 0 | TCGA-ED-A7XO | 427 | 29 | male | Primary Tumor | G2 | stage iiia | 64 |  | Mut | 0 | 0 | 0.382647 | 0 | 0 | 0 | 0.617353 | 0.342 | 0.097464 | 1.051446 | cluster 1 | High | High | Low |
| TCGA-DD-AACB-01A | 0 | TCGA-DD-AACB | 2324 | 74 | female | Primary Tumor | G3 | stage i | 72 | 6 - Established Cirrhosis | Mut | 0 | 0 | 0.46622 | 0 | 0 | 0 | 0.53378 | 0.886 | -0.09909 | 1.126256 | cluster 1 | High | High | Low |
| TCGA-RC-A7SH-01A | 0 | TCGA-RC-A7SH | 468 | 42 | male | Primary Tumor | G3 | stage ii | 68 | 3,4 - Fibrous Speta | Mut | 0 | 0 | 0.307542 | 0 | 0 | 0 | 0.692458 | 0.234 | 0.1861 | 1.015228 | cluster 1 | High | Low | Low |
| TCGA-CC-5261-01A | 1 | TCGA-CC-5261 | 97 | 44 | male | Primary Tumor | G2 | stage ii | 58 |  | WT | 0.143296 | 0 | 0.283807 | 0 | 0.08942 | 0 | 0.483477 | 0.321 | 0.109112 | 1.037017 | cluster 2 | Low | Low | High |
| TCGA-DD-A3A8-01A | 1 | TCGA-DD-A3A8 | 11 | 75 | male | Primary Tumor | G2 | stage ii | 99 | 0 - No Fibrosis | WT | 0 | 0 | 0.266268 | 0 | 0 | 0 | 0.733732 | 0.367 | 0.080011 | 1.063982 | cluster 1 | High | Low | Low |
| TCGA-DD-A73C-01A | 0 | TCGA-DD-A73C | 701 | 65 | female | Primary Tumor | G1 | stage iiia | 49 | 0 - No Fibrosis | WT | 0 | 0 | 0.250822 | 0 | 0.004105 | 0.002292 | 0.742781 | 0.272 | 0.14686 | 1.035415 | cluster 2 | High | Low | Low |
| TCGA-GJ-A9DB-01A | 1 | TCGA-GJ-A9DB | 67 | 68 | male | Primary Tumor | G2 | stage i | 96 |  | WT | 0 | 0 | 0.332444 | 0 | 0.005189 | 0 | 0.662367 | 0.28 | 0.139561 | 1.034773 | cluster 2 | High | High | Low |
| TCGA-DD-AAW1-01A | 0 | TCGA-DD-AAW1 | 1989 | 55 | male | Primary Tumor | G2 | stage iiia | 60 | 6 - Established Cirrhosis | WT | 0 | 0 | 0.237079 | 0 | 0 | 0 | 0.762921 | 0.404 | 0.053662 | 1.077478 | cluster 1 | High | Low | Low |
| TCGA-XR-A8TF-01A | 1 | TCGA-XR-A8TF | 693 | 74 | male | Primary Tumor | G1 | stage i | 57 | 3,4 - Fibrous Speta | WT | 0 | 0 | 0.259224 | 0 | 0 | 0.008011 | 0.732766 | 0.363 | 0.082229 | 1.065664 | cluster 1 | High | Low | Low |
| TCGA-CC-A5UC-01A | 1 | TCGA-CC-A5UC | 347 | 63 | male | Primary Tumor | G3 | stage iiia | 57 |  | WT | 0 | 0 | 0.353492 | 0 | 0 | 0 | 0.646508 | 0.35 | 0.091707 | 1.054766 | cluster 1 | High | High | Low |
| TCGA-DD-A39Y-01A | 1 | TCGA-DD-A39Y | 171 | 67 | male | Primary Tumor | G3 | stage i | 85 | 0 - No Fibrosis | WT | 0 | 0 | 0.276861 | 0 | 0.083504 | 0.004074 | 0.635561 | 0.521 | 0.004135 | 1.093236 | cluster 2 | High | Low | Low |
| TCGA-DD-A114-01A | 1 | TCGA-DD-A114 | 1149 | 42 | male | Primary Tumor | G3 | stage ii | 74 | 5 - Nodular Formation and Incomplete Cirrhosis | Mut | 0.282654 | 0 | 0.384578 | 0 | 0.332768 | 0 | 0 | 0.353 | 0.088885 | 1.055684 | cluster 2 | Low | High | High |
| TCGA-NI-A8LF-01A | 0 | TCGA-NI-A8LF | 799 | 74 | male | Primary Tumor | G3 | stage i | 104 |  | Mut | 0 | 0 | 0.172214 | 0 | 0 | 0.01537 | 0.812416 | 0.361 | 0.083271 | 1.073762 | cluster 1 | High | Low | Low |
| TCGA-DD-AADF-01A | 1 | TCGA-DD-AADF | 115 | 64 | female | Primary Tumor | G4 | stage i | 55 |  | WT | 0.122956 | 0 | 0.338738 | 0 | 0.020613 | 0 | 0.517693 | 0.681 | -0.04528 | 1.097805 | cluster 2 | Low | High | High |
| TCGA-DD-AAEI-01A | 0 | TCGA-DD-AAEI | 1531 | 72 | male | Primary Tumor | G2 | stage i | 79 | 6 - Established Cirrhosis | Mut | 0 | 0 | 0.282092 | 0 | 0 | 0.015619 | 0.702289 | 0.306 | 0.120739 | 1.049276 | cluster 1 | High | Low | Low |
| TCGA-2Y-A9H5-01A | 1 | TCGA-2Y-A9H5 | 555 | 59 | female | Primary Tumor | G3 | stage i | 69 | 1,2 - Portal Fibrosis | WT | 0 | 0 | 0.353339 | 0 | 0 | 0 | 0.646661 | 0.297 | 0.127479 | 1.039591 | cluster 1 | High | High | Low |

**Table S3**. Prognosis/treatment marker identification

|  | **p_val** | **avg_log2FC** | **pct.1** | **pct.2** | **p_val_adj** |
| --- | --- | --- | --- | --- | --- |
| CYP2E1 | 0.00E+00 | 2.223595 | 0.687 | 0.009 | 0.00E+00 |
| HPD | 6.35E-271 | 1.73211 | 0.599 | 0.035 | 1.63E-266 |
| GSTA1 | 1.63E-239 | 2.086991 | 0.784 | 0.086 | 4.19E-235 |
| APOH | 7.36E-202 | 3.063292 | 0.991 | 0.27 | 1.89E-197 |
| TTR | 5.33E-196 | 3.283717 | 1 | 0.304 | 1.37E-191 |
| HPX | 3.74E-174 | 1.528408 | 0.599 | 0.065 | 9.62E-170 |
| HP | 3.82E-168 | 2.065858 | 0.859 | 0.169 | 9.82E-164 |
| APOC3 | 2.55E-161 | 2.707381 | 1 | 0.405 | 6.57E-157 |
| APOA1 | 1.15E-155 | 2.941069 | 1 | 0.516 | 2.96E-151 |
| RBP4 | 6.57E-155 | 2.363857 | 0.996 | 0.349 | 1.69E-150 |
| FGA | 2.72E-140 | 1.437982 | 0.656 | 0.097 | 6.98E-136 |
| APOC1 | 2.29E-139 | 2.416382 | 1 | 0.602 | 5.88E-135 |
| ALB | 7.16E-134 | 2.357616 | 1 | 0.695 | 1.84E-129 |

| DCXR | 5.01E-130 | 1.880348 | 0.855 | 0.219 | 1.29E-125 |
| --- | --- | --- | --- | --- | --- |
| SERPINC1 | 6.73E-130 | 1.487152 | 0.626 | 0.099 | 1.73E-125 |
| APOE | 5.99E-118 | 1.764429 | 1 | 0.677 | 1.54E-113 |
| PRAP1 | 4.27E-110 | 1.669305 | 0.846 | 0.233 | 1.10E-105 |
| MGST1 | 3.66E-107 | 1.395069 | 0.696 | 0.14 | 9.42E-103 |
| FGB | 6.55E-101 | 1.235316 | 0.639 | 0.121 | 1.68E-96 |
| VTN | 7.49E-98 | 1.591158 | 0.921 | 0.338 | 1.92E-93 |
| AMBP | 7.60E-95 | 1.600046 | 0.938 | 0.4 | 1.96E-90 |
| GC | 3.99E-92 | 1.084468 | 0.617 | 0.121 | 1.03E-87 |
| C3 | 1.58E-87 | 1.077597 | 0.524 | 0.094 | 4.05E-83 |
| ORM1 | 1.13E-84 | 1.347203 | 0.661 | 0.155 | 2.91E-80 |
| APOA2 | 9.00E-84 | 1.571049 | 1 | 0.773 | 2.31E-79 |
| FABP1 | 1.21E-79 | 1.296672 | 0.577 | 0.125 | 3.11E-75 |
| AHSG | 8.32E-79 | 1.262852 | 0.643 | 0.157 | 2.14E-74 |
| CES1 | 2.11E-63 | 1.05274 | 0.568 | 0.14 | 5.43E-59 |
| CYB5A | 9.59E-63 | 1.202122 | 0.656 | 0.195 | 2.47E-58 |
| FGG | 2.33E-60 | 0.899224 | 0.639 | 0.172 | 5.98E-56 |
| RPS3A | 9.82E-50 | 0.721429 | 0.996 | 0.957 | 2.53E-45 |
| A1BG | 1.49E-49 | 0.97137 | 0.524 | 0.149 | 3.82E-45 |
| SEPP1 | 2.33E-48 | 1.203386 | 0.683 | 0.267 | 5.98E-44 |
| MT1X | 4.83E-45 | 1.01793 | 0.753 | 0.3 | 1.24E-40 |
| MT-ND1 | 2.15E-41 | 0.796055 | 0.996 | 0.785 | 5.53E-37 |
| TF | 7.75E-41 | 0.909702 | 0.52 | 0.163 | 1.99E-36 |
| CD69 | 2.15E-40 | 1.434782 | 0.903 | 0.588 | 5.52E-36 |
| MT-CO1 | 1.43E-39 | 0.490147 | 1 | 0.998 | 3.69E-35 |
| C1QA | 4.12E-39 | 1.003778 | 0.648 | 0.25 | 1.06E-34 |
| RARRES2 | 6.42E-38 | 0.864719 | 0.639 | 0.24 | 1.65E-33 |
| DUSP1 | 7.31E-37 | 0.951799 | 0.987 | 0.661 | 1.88E-32 |
| TXNIP | 2.68E-35 | 1.080572 | 0.916 | 0.576 | 6.89E-31 |
| NFKBIA | 3.97E-35 | 1.472781 | 0.93 | 0.64 | 1.02E-30 |
| C1QB | 5.76E-35 | 0.907875 | 0.568 | 0.206 | 1.48E-30 |
| B2M | 5.23E-34 | -0.44089 | 1 | 1 | 1.34E-29 |
| MT-ND2 | 5.89E-34 | 0.590742 | 1 | 0.93 | 1.51E-29 |
| PEBP1 | 6.34E-34 | 0.898644 | 0.815 | 0.412 | 1.63E-29 |
| CRIP1 | 2.11E-33 | -1.51273 | 0.537 | 0.756 | 5.43E-29 |
| H3F3B | 1.09E-32 | -0.73129 | 1 | 0.992 | 2.80E-28 |
| RPL36A | 2.33E-30 | -0.95723 | 0.89 | 0.888 | 6.00E-26 |
| RPS12 | 2.84E-29 | 0.544516 | 1 | 0.979 | 7.30E-25 |
| CREM | 3.60E-28 | -1.23268 | 0.678 | 0.84 | 9.24E-24 |
| RPL28 | 6.45E-28 | 0.44261 | 1 | 0.991 | 1.66E-23 |
| RPL13 | 2.04E-27 | 0.580917 | 1 | 0.85 | 5.25E-23 |
| BTG1 | 4.73E-27 | 0.656498 | 1 | 0.847 | 1.22E-22 |
| CLU | 7.01E-27 | 0.615569 | 0.67 | 0.295 | 1.80E-22 |
| ITM2B | 8.37E-25 | 0.623579 | 0.947 | 0.655 | 2.15E-20 |
| EEF1A1 | 4.47E-23 | 0.411712 | 1 | 0.994 | 1.15E-18 |
| KLRB1 | 3.37E-21 | 0.915274 | 0.731 | 0.431 | 8.67E-17 |
| PTGER4 | 7.66E-21 | 0.7168 | 0.608 | 0.304 | 1.97E-16 |
| RPL41 | 1.95E-20 | -0.36306 | 1 | 0.999 | 5.02E-16 |
| DUSP2 | 2.35E-20 | 0.664293 | 0.846 | 0.564 | 6.03E-16 |
| TMSB4X | 4.33E-20 | -0.42999 | 1 | 1 | 1.11E-15 |
| CST3 | 6.92E-20 | 0.80654 | 0.696 | 0.37 | 1.78E-15 |
| DDT | 1.58E-19 | 0.615714 | 0.639 | 0.323 | 4.06E-15 |
| MT-CYB | 3.46E-19 | 0.438846 | 1 | 0.94 | 8.90E-15 |
| MT-CO3 | 4.92E-19 | 0.350774 | 1 | 0.988 | 1.26E-14 |
| RPS20 | 1.95E-18 | 0.393991 | 0.996 | 0.959 | 5.02E-14 |
| CACYBP | 2.84E-18 | -1.4723 | 0.511 | 0.65 | 7.31E-14 |
| GZMK | 3.09E-18 | 0.551994 | 0.621 | 0.32 | 7.96E-14 |
| RPL19 | 5.26E-18 | 0.338293 | 1 | 0.985 | 1.35E-13 |
| MT-CO2 | 8.02E-18 | 0.330426 | 1 | 0.981 | 2.06E-13 |
| LGALS1 | 1.02E-17 | -1.14731 | 0.586 | 0.73 | 2.62E-13 |
| RPL30 | 2.16E-17 | 0.349199 | 1 | 0.973 | 5.57E-13 |
| DYNLL1 | 6.50E-17 | -1.02076 | 0.608 | 0.714 | 1.67E-12 |
| LDHA | 9.76E-17 | -0.92839 | 0.696 | 0.72 | 2.51E-12 |
| JUNB | 1.16E-16 | 0.478916 | 0.956 | 0.705 | 2.98E-12 |
| RPLP2 | 2.17E-16 | 0.333825 | 1 | 0.989 | 5.58E-12 |
| CALM2 | 3.28E-16 | -0.73139 | 0.863 | 0.855 | 8.43E-12 |
| BIN2 | 3.61E-16 | 0.530005 | 0.529 | 0.26 | 9.29E-12 |
| RPS2 | 8.53E-16 | 0.462031 | 1 | 0.801 | 2.19E-11 |
| MCL1 | 1.05E-15 | 0.585783 | 0.899 | 0.647 | 2.69E-11 |
| RPL17 | 1.17E-15 | -0.85521 | 0.85 | 0.805 | 3.00E-11 |
| ZFP36L2 | 1.38E-15 | 0.499648 | 0.943 | 0.708 | 3.54E-11 |
| OAZ1 | 1.68E-15 | -0.61014 | 0.965 | 0.929 | 4.32E-11 |
| PNRC1 | 4.14E-15 | 0.514119 | 0.841 | 0.572 | 1.07E-10 |
| MALAT1 | 4.68E-15 | -0.40173 | 1 | 1 | 1.20E-10 |
| SELK | 6.71E-15 | -0.83867 | 0.599 | 0.723 | 1.72E-10 |
| RORA | 8.19E-15 | 0.695625 | 0.648 | 0.407 | 2.11E-10 |
| HSPD1 | 2.01E-14 | -1.28608 | 0.595 | 0.706 | 5.17E-10 |
| PIK3R1 | 3.02E-14 | 0.641078 | 0.559 | 0.319 | 7.76E-10 |
| RPS8 | 3.14E-14 | 0.339725 | 1 | 0.974 | 8.07E-10 |
| UBA52 | 3.17E-14 | 0.332359 | 1 | 0.921 | 8.14E-10 |
| RARRES3 | 5.67E-14 | 0.493937 | 0.727 | 0.439 | 1.46E-09 |
| CUTA | 9.94E-14 | 0.478086 | 0.775 | 0.484 | 2.56E-09 |
| RPL32 | 1.92E-13 | 0.258245 | 1 | 0.995 | 4.94E-09 |
| JUN | 2.57E-13 | 0.467254 | 0.907 | 0.648 | 6.61E-09 |
| GLTSCR2 | 3.22E-13 | 0.47811 | 0.802 | 0.49 | 8.27E-09 |
| RPS4Y1 | 3.22E-13 | 0.372544 | 0.815 | 0.461 | 8.29E-09 |
| RPL10 | 4.16E-13 | 0.256556 | 1 | 0.999 | 1.07E-08 |
| MT-ND4 | 4.41E-13 | 0.297827 | 1 | 0.981 | 1.13E-08 |
| H2AFZ | 4.95E-13 | -1.00344 | 0.586 | 0.673 | 1.27E-08 |
| IL7R | 1.14E-12 | 0.563354 | 0.793 | 0.589 | 2.93E-08 |
| RPL11 | 1.40E-12 | 0.260669 | 1 | 0.985 | 3.59E-08 |
| SAMSN1 | 2.37E-12 | -0.84117 | 0.67 | 0.728 | 6.09E-08 |
| CCL4 | 3.38E-12 | 1.438627 | 0.648 | 0.459 | 8.69E-08 |
| RPL7A | 3.41E-12 | 0.351773 | 1 | 0.89 | 8.76E-08 |
| ATP5D | 5.67E-12 | 0.406226 | 0.648 | 0.367 | 1.46E-07 |
| RPL29 | 7.54E-12 | 0.279768 | 1 | 0.947 | 1.94E-07 |
| S100A11 | 1.91E-11 | -0.70678 | 0.714 | 0.747 | 4.91E-07 |
| RPL37 | 1.95E-11 | 0.295043 | 1 | 0.941 | 5.01E-07 |
| HSP90AB1 | 2.22E-11 | -0.81542 | 0.916 | 0.913 | 5.71E-07 |
| CCNI | 4.72E-11 | 0.435443 | 0.797 | 0.519 | 1.21E-06 |
| DNAJA1 | 1.02E-10 | -0.87702 | 0.833 | 0.843 | 2.61E-06 |
| YPEL5 | 1.14E-10 | -0.88531 | 0.564 | 0.659 | 2.94E-06 |
| MT-ND5 | 1.36E-10 | 0.373229 | 0.828 | 0.599 | 3.49E-06 |
| RPS23 | 1.49E-10 | 0.264611 | 1 | 0.975 | 3.82E-06 |
| CD48 | 1.62E-10 | 0.408906 | 0.687 | 0.422 | 4.15E-06 |
| HSPH1 | 1.68E-10 | -1.1294 | 0.502 | 0.615 | 4.33E-06 |
| KLF6 | 1.75E-10 | 0.379545 | 0.938 | 0.698 | 4.51E-06 |
| RPS17 | 1.90E-10 | 0.277311 | 0.996 | 0.96 | 4.88E-06 |
| EEF1B2 | 1.99E-10 | 0.385243 | 0.912 | 0.685 | 5.10E-06 |
| GLUL | 2.32E-10 | 0.307181 | 0.581 | 0.335 | 5.97E-06 |
| P4HB | 3.89E-10 | 0.388219 | 0.515 | 0.286 | 1.00E-05 |
| MT-ND3 | 3.91E-10 | 0.283779 | 1 | 0.862 | 1.00E-05 |
| ALDOA | 4.25E-10 | -0.70789 | 0.744 | 0.726 | 1.09E-05 |
| UBB | 7.99E-10 | -0.72309 | 0.965 | 0.949 | 2.05E-05 |
| DNAJB6 | 8.33E-10 | -0.8072 | 0.599 | 0.655 | 2.14E-05 |
| SAP18 | 9.14E-10 | -0.65987 | 0.546 | 0.634 | 2.35E-05 |
| PIP4K2A | 1.03E-09 | 0.390642 | 0.52 | 0.298 | 2.65E-05 |
| CITED2 | 1.24E-09 | 0.467183 | 0.52 | 0.315 | 3.20E-05 |
| SUB1 | 1.50E-09 | -0.52086 | 0.815 | 0.805 | 3.86E-05 |
| EIF3F | 1.66E-09 | 0.38594 | 0.7 | 0.447 | 4.28E-05 |
| MT2A | 1.86E-09 | 0.261825 | 0.841 | 0.592 | 4.79E-05 |
| RPL9 | 2.27E-09 | 0.271729 | 1 | 0.938 | 5.84E-05 |
| GAPDH | 3.62E-09 | -0.63807 | 0.987 | 0.957 | 9.30E-05 |
| RPS11 | 3.84E-09 | 0.277773 | 0.991 | 0.889 | 9.88E-05 |
| C19orf43 | 4.00E-09 | 0.363047 | 0.687 | 0.437 | 1.03E-04 |
| IER2 | 4.21E-09 | 0.396938 | 0.67 | 0.435 | 1.08E-04 |
| SMCHD1 | 5.11E-09 | 0.346629 | 0.546 | 0.321 | 1.31E-04 |
| GUK1 | 5.41E-09 | 0.36739 | 0.762 | 0.487 | 1.39E-04 |
| TPI1 | 7.08E-09 | -0.84881 | 0.678 | 0.691 | 1.82E-04 |
| CXCR4 | 8.45E-09 | 0.298474 | 0.978 | 0.941 | 2.17E-04 |
| RBM8A | 1.41E-08 | -0.59206 | 0.626 | 0.657 | 3.63E-04 |
| PPP1R15A | 2.16E-08 | 0.48622 | 0.709 | 0.493 | 5.55E-04 |
| SFPQ | 2.75E-08 | 0.314947 | 0.559 | 0.336 | 7.08E-04 |
| TNFAIP3 | 2.76E-08 | 0.349756 | 0.833 | 0.632 | 7.10E-04 |
| TERF2IP | 3.13E-08 | 0.349834 | 0.612 | 0.398 | 8.06E-04 |
| CCND3 | 3.93E-08 | 0.279442 | 0.564 | 0.336 | 1.01E-03 |
| COX7A2 | 5.16E-08 | -0.49759 | 0.775 | 0.727 | 1.33E-03 |
| PGK1 | 5.50E-08 | -0.72106 | 0.573 | 0.608 | 1.41E-03 |
| FNBP1 | 5.56E-08 | 0.359155 | 0.595 | 0.375 | 1.43E-03 |
| RPSA | 5.62E-08 | 0.286313 | 0.996 | 0.89 | 1.45E-03 |
| UBC | 1.14E-07 | -0.5806 | 0.987 | 0.969 | 2.92E-03 |
| IRF1 | 1.93E-07 | 0.307097 | 0.529 | 0.325 | 4.97E-03 |
| RPL5 | 2.48E-07 | 0.250962 | 1 | 0.904 | 6.37E-03 |
| MTDH | 2.59E-07 | 0.362996 | 0.52 | 0.328 | 6.66E-03 |
| HSPE1 | 2.73E-07 | -0.98997 | 0.793 | 0.792 | 7.03E-03 |
| RPS27L | 3.09E-07 | -0.67173 | 0.537 | 0.597 | 7.94E-03 |
| TUBA1B | 3.33E-07 | -1.06854 | 0.568 | 0.592 | 8.56E-03 |
| GTF3A | 3.93E-07 | 0.261085 | 0.577 | 0.349 | 1.01E-02 |
| HSP90AA1 | 4.07E-07 | -0.88921 | 0.987 | 0.947 | 1.05E-02 |
| HSPA8 | 4.43E-07 | -0.67492 | 0.969 | 0.932 | 1.14E-02 |
| SKP1 | 5.18E-07 | -0.45232 | 0.793 | 0.751 | 1.33E-02 |
| GSTK1 | 6.16E-07 | 0.333726 | 0.687 | 0.471 | 1.58E-02 |
| TAGLN2 | 7.25E-07 | -0.6308 | 0.595 | 0.634 | 1.86E-02 |
| SRP14 | 1.34E-06 | -0.40183 | 0.921 | 0.856 | 3.45E-02 |
| ARF6 | 1.82E-06 | 0.314616 | 0.727 | 0.504 | 4.69E-02 |

**Table S4.** Genetic GSEA analysis of APOC3, APOH, HPX and FGB

| **ID** | **Description** | **Set Size** | **Enrichment Score** | **NES** | **P value** | **p. adjust** | **Q values** | **rank** | **leading_edge** | **core_enrichment** |
| --- | --- | --- | --- | --- | --- | --- | --- | --- | --- | --- |
| KEGG_COMPLEMENT_AND_COAGULATION_CASCADES | KEGG_COMPLEMENT_AND_COAGULATION_CASCADES | 69 | 0.835272 | 3.317157 | 1.00E-10 | 1.84E-08 | 1.14E-08 | 6097 | tags=75%, list=11%, signal=67% | C9/FGB/FGG/FGA/C1R/C8B/C6/CFH/CFI/C7/C1S/C5/C4BPA/C8G/C8A/SERPINA1/SERPING1/MASP2/CFB/C4B/BDKRB2/F11/C4A/C3/F9/F13B/MBL2/A2M/C4BPB/F13A1/PLG/C5AR1/THBD/CR1/KLKB1/SERPINF2/SERPINC1/F8/PLAT/VWF/C1QA/CD55/C1QB/F2/PROS1/KNG1/F12/CPB2/CD59/C3AR1/C1QC/F2R |
| KEGG_GRAFT_VERSUS_HOST_DISEASE | KEGG_GRAFT_VERSUS_HOST_DISEASE | 37 | 0.750309 | 2.652464 | 1.67E-09 | 1.53E-07 | 9.48E-08 | 7372 | tags=68%, list=13%, signal=59% | IL6/HLA-DRB5/IL2/HLA-F/HLA-B/HLA-DPA1/HLA-DQB1/HLA-DRB1/HLA-DRA/KLRD1/HLA-DMA/KLRC1/HLA-DMB/CD28/HLA-DOA/HLA-DPB1/HLA-C/FAS/HLA-A/PRF1/HLA-E/HLA-DOB/HLA-DQA2/KIR2DL1/HLA-G |
| KEGG_CYTOKINE_CYTOKINE_RECEPTOR_INTERACTION | KEGG_CYTOKINE_CYTOKINE_RECEPTOR_INTERACTION | 259 | 0.417994 | 2.014129 | 2.81E-09 | 1.72E-07 | 1.06E-07 | 5912 | tags=33%, list=11%, signal=30% | IL6/CXCL2/IL20/PPBP/TNFSF14/PF4/CSF3/PF4V1/IL25/CXCL6/IL18R1/IL2/EGFR/CXCL16/CRLF2/CCL19/XCR1/CXCL8/HGF/CXCL1/TSLP/EPO/CXCR6/IL18RAP/CCL13/CXCL3/CCL11/XCL2/IL7R/OSMR/CCL18/TNFRSF1B/CCR8/CCR7/CXCR2/CCL24/CXCR1/TNFSF18/LIFR/AMHR2/CCL21/TPO/CXCL12/GHR/IFNGR1/EGF/IL7/IL13RA1/FLT3/CD40LG/KDR/CCL22/CCL4/CCR4/IL12B/IL4R/IL2RB/CCR6/TNFRSF1A/TNFSF8/NGFR/CCL23/FLT4/IL24/TNFSF13/IL6ST/IL3RA/EDA2R/BMP2/BMPR1B/CD70/TNFSF13B/CCL3/FAS/VEGFC/TGFB3/TNFRSF10A/CXCR4/CCL25/IL2RA/CCL3L3/FLT1/ACVRL1/IFNK/IL10 |
| KEGG_SYSTEMIC_LUPUS_ERYTHEMATOSUS | KEGG_SYSTEMIC_LUPUS_ERYTHEMATOSUS | 55 | 0.624844 | 2.431011 | 2.83E-07 | 1.30E-05 | 8.04E-06 | 4190 | tags=51%, list=7%, signal=47% | C9/HLA-DRB5/C1R/C8B/C6/C7/C1S/C5/C8G/C8A/C4B/CTSG/C4A/C3/HLA-DPA1/HLA-DQB1/HLA-DRB1/HLA-DRA/CD40LG/HLA-DMA/C1QA/HLA-DMB/GRIN2B/CD28/ELANE/HLA-DOA/HLA-DPB1/C1QB |
| KEGG_PRION_DISEASES | KEGG_PRION_DISEASES | 35 | 0.701692 | 2.454916 | 3.81E-07 | 1.34E-05 | 8.28E-06 | 6293 | tags=49%, list=11%, signal=43% | C9/IL6/C8B/C6/C7/C5/C8G/C8A/EGR1/C1QA/C1QB/HSPA5/SOD1/NCAM2/C1QC/PRKACA/FYN |
| KEGG_VIRAL_MYOCARDITIS | KEGG_VIRAL_MYOCARDITIS | 68 | 0.58734 | 2.334363 | 4.37E-07 | 1.34E-05 | 8.28E-06 | 7372 | tags=44%, list=13%, signal=38% | MYH4/MYH1/MYH8/MYH2/HLA-DRB5/HLA-F/HLA-B/HLA-DPA1/HLA-DQB1/ICAM1/HLA-DRB1/MYH11/HLA-DRA/SGCA/CD40LG/LAMA2/HLA-DMA/CD55/HLA-DMB/CD28/HLA-DOA/HLA-DPB1/HLA-C/HLA-A/FYN/PRF1/HLA-E/HLA-DOB/HLA-DQA2/HLA-G |
| KEGG_ALLOGRAFT_REJECTION | KEGG_ALLOGRAFT_REJECTION | 35 | 0.675538 | 2.363416 | 1.66E-06 | 4.37E-05 | 2.70E-05 | 7372 | tags=69%, list=13%, signal=60% | HLA-DRB5/IL2/HLA-F/HLA-B/HLA-DPA1/HLA-DQB1/HLA-DRB1/HLA-DRA/CD40LG/HLA-DMA/IL12B/HLA-DMB/CD28/HLA-DOA/HLA-DPB1/HLA-C/FAS/HLA-A/IL10/PRF1/HLA-E/HLA-DOB/HLA-DQA2/HLA-G |
| KEGG_ARGININE_AND_PROLINE_METABOLISM | KEGG_ARGININE_AND_PROLINE_METABOLISM | 53 | 0.596179 | 2.288578 | 3.85E-06 | 8.85E-05 | 5.47E-05 | 4010 | tags=34%, list=7%, signal=32% | PRODH/GLS2/ASS1/OAT/GOT1/ARG1/AOC1/CPS1/ASL/ARG2/SAT1/LAP3/P4HA1/ALDH4A1/NOS3/OTC/ALDH2/GOT2 |
| KEGG_CELL_CYCLE | KEGG_CELL_CYCLE | 124 | -0.53066 | -1.99169 | 4.50E-06 | 9.20E-05 | 5.68E-05 | 17001 | tags=60%, list=30%, signal=42% | EP300/BUB3/CDC27/ZBTB17/E2F4/ANAPC5/CDKN2B/CDC14A/CDK7/MAD2L1/GSK3B/YWHAQ/CCND3/RBX1/PCNA/ORC3/E2F5/CDC23/ORC2/ANAPC11/WEE2/CDKN2C/TFDP1/ABL1/ANAPC4/CDC25B/ANAPC1/CDC7/SMC3/CCND2/HDAC2/ANAPC7/SMAD3/CDK2/CDK4/E2F3/DBF4/MCM4/CCNB3/ORC1/CDC25C/MCM6/SFN/ESPL1/MCM5/RBL1/CDC6/TGFB2/CCNA1/CDKN2A/CCNB1/MCM7/BUB1/PKMYT1/E2F2/MCM3/E2F1/CHEK1/CDK6/ORC6/CDC45/CCNE1/MCM2/CDK1/PTTG1/CCNB2/BUB1B/PLK1/TTK/CDC20/TGFB1/SKP2/CDC25A/SMC1B/CDKN1C |
| KEGG_ANTIGEN_PROCESSING_AND_PRESENTATION | KEGG_ANTIGEN_PROCESSING_AND_PRESENTATION | 79 | 0.515441 | 2.103989 | 6.85E-06 | 0.000126 | 7.78E-05 | 9451 | tags=44%, list=17%, signal=37% | HLA-DRB5/B2M/HLA-F/HLA-B/HLA-DPA1/KIR3DL3/HLA-DQB1/CD74/HLA-DRB1/HLA-DRA/KLRD1/CTSS/CD4/HLA-DMA/KLRC1/HLA-DMB/HLA-DOA/HLA-DPB1/CIITA/HSPA5/HLA-C/LGMN/HLA-A/HLA-E/HLA-DOB/HLA-DQA2/KLRC3/KIR2DL1/HLA-G/KIR2DL4/KLRC4/PDIA3/IFNA6/TAP2/CTSL |
| KEGG_NEUROACTIVE_LIGAND_RECEPTOR_INTERACTION | KEGG_NEUROACTIVE_LIGAND_RECEPTOR_INTERACTION | 271 | -0.4277 | -1.73841 | 1.04E-05 | 0.000174 | 0.000107 | 4935 | tags=24%, list=9%, signal=22% | GPR35/SSTR5/GIPR/GRIA4/GRIN2A/HTR2B/DRD4/CALCR/LPAR1/GLP2R/OPRL1/GABRB2/PTGFR/GLRA2/P2RX7/CHRNA7/CHRNB4/CHRM4/OPRD1/UTS2R/GRIN1/CHRM1/LHB/GHSR/SSTR2/GALR3/TACR2/GALR1/ADORA2B/GABRG3/GRIK4/PRLHR/GRM1/DRD2/SCTR/CHRNB3/CHRNA9/GABBR2/NPFFR2/NPY4R/GABRA3/GRID2/LPAR2/LEP/GABRQ/GRM3/ADORA1/CHRNA1/ADRA2C/CHRNA3/AVPR1B/GRIA2/CHRNB2/KISS1R/PRL/GRPR/GPR50/GRM5/SSTR3/CCKBR/TACR3/HTR1D/CRHR1/MTNR1B/PRSS1 |
| KEGG_RIBOSOME | KEGG_RIBOSOME | 88 | -0.55326 | -1.97407 | 1.30E-05 | 0.000192 | 0.000119 | 17110 | tags=67%, list=30%, signal=47% | RPS27/RPS15A/RPS17/RPL36A/RPS28/RPS2/RPL37/RPS15/RPL3/RPL13/RPLP1/RPS13/RPS23/RPL35/RPS4X/RPL24/RPL6/RPL22L1/RPL12/RPL31/RPS19/RPL7A/RPL4/RPS6/RPS8/RPS27A/RPS12/RPL15/RPL18A/RPL35A/RPL19/RPL5/RPLP0/RPL37A/RPS16/RPL38/RPS11/RPL28/RPS25/RPL27/RPL29/RPS18/RPL23/RPS21/RPL9/RPS10/RPL18/RPL17/RPLP2/RPL27A/RPL32/RPL13A/RPS24/RPS5/RPS7/RPS3/RPL14/RPL23A/RPSA/RPL10L |
| KEGG_AUTOIMMUNE_THYROID_DISEASE | KEGG_AUTOIMMUNE_THYROID_DISEASE | 48 | 0.59018 | 2.226388 | 1.36E-05 | 0.000192 | 0.000119 | 7372 | tags=54%, list=13%, signal=47% | CGA/HLA-DRB5/IL2/HLA-F/HLA-B/HLA-DPA1/HLA-DQB1/TPO/HLA-DRB1/HLA-DRA/CD40LG/HLA-DMA/HLA-DMB/CD28/HLA-DOA/HLA-DPB1/HLA-C/FAS/HLA-A/CTLA4/IL10/PRF1/HLA-E/HLA-DOB/HLA-DQA2/HLA-G |
| KEGG_FOLATE_BIOSYNTHESIS | KEGG_FOLATE_BIOSYNTHESIS | 10 | 0.870014 | 2.181369 | 2.54E-05 | 0.000323 | 0.0002 | 5081 | tags=80%, list=9%, signal=73% | ALPI/GCH1/ALPL/GGH/QDPR/ALPP/FPGS/PTS |
| KEGG_GLYCINE_SERINE_AND_THREONINE_METABOLISM | KEGG_GLYCINE_SERINE_AND_THREONINE_METABOLISM | 31 | 0.6586 | 2.239356 | 2.63E-05 | 0.000323 | 0.0002 | 7503 | tags=47%, list=13%, signal=41% | SDS/CTH/AGXT2/CBS/GNMT/SHMT1/AGXT/SARDH/GLDC/ALAS1/DMGDH/AOC3/CBS/GAMT/ALAS2 |
| KEGG_ARACHIDONIC_ACID_METABOLISM | KEGG_ARACHIDONIC_ACID_METABOLISM | 58 | 0.553448 | 2.155569 | 4.82E-05 | 0.000554 | 0.000343 | 6941 | tags=38%, list=12%, signal=33% | PLA2G2A/PLA2G2F/CYP2B6/PTGIS/PLA2G1B/GPX2/PTGS2/CYP2C18/CYP2C9/CYP2C8/GPX5/GPX3/CYP4F3/CYP4A11/PLA2G5/PLA2G12A/PLA2G4A/GGT5/HPGDS/CYP2E1/CYP4F2/PLA2G4B |
| KEGG_OLFACTORY_TRANSDUCTION | KEGG_OLFACTORY_TRANSDUCTION | 351 | 0.299157 | 1.486442 | 8.33E-05 | 0.000902 | 0.000557 | 19892 | tags=61%, list=35%, signal=40% | CLCA1/OR52N2/OR52N1/OR52N5/CLCA2/OR2W3/OR51Q1/PDE1C/OR1N2/OR1F1/OR7D2/OR51B4/OR52N4/OR4C3/OR2L13/OR2A1/OR52E6/OR56B1/OR5AK2/OR52I2/OR51E2/OR2Z1/OR13C5/OR52D1/OR10J5/OR4F17/OR2B3/OR51E1/OR4F4/OR51B5/OR10J3/CNGB1/OR1C1/PRKACA/OR2T8/OR10V1/OR51M1/OR4X2/OR1L8/ARRB2/OR52E4/OR4A47/OR4C12/OR8D2/OR10A4/OR2L2/OR6C4/OR6F1/OR13F1/OR6K3/OR6K2/OR10H3/OR10J1/OR4C13/OR2G2/OR1N1/OR6T1/OR51G2/OR52M1/OR1A1/OR4D11/OR13C2/OR2V2/OR13C8/OR10K2/OR2Y1/OR10S1/OR51B6/OR52B2/OR13J1/PRKG1/OR2A12/OR6C68/OR6C6/OR4D5/OR6C3/OR7G3/OR2D3/OR6C65/OR14C36/OR5P3/OR1A2/OR11H1/OR9K2/OR1D4/OR4A15/OR51A7/OR51I1/OR2AG2/OR10G2/OR5D16/OR4F3/OR7A10/OR5AR1/OR5T3/OR5M3/OR4K13/OR51V1/OR4P4/OR4C15/OR4N5/OR5D14/OR10T2/OR6N2/OR10G8/OR51G1/OR5I1/OR4F16/OR4D6/OR5AN1/OR7A17/OR6A2/OR10A2/OR51I2/OR6C1/OR4D1/OR10K1/OR5D18/OR2W1/OR8K5/OR13C9/OR14A16/OR6N1/OR10G9/OR10A5/OR11L1/OR2L3/OR4D2/OR52H1/OR5K1/OR5AC2/OR6V1/OR6X1/OR8I2/OR4K17/OR6K6/OR4C45/OR9G1/OR51L1/OR4F15/OR7G1/OR5A2/OR7D4/OR2M7/OR2T29/OR2T5/OR5M10/OR10P1/OR7C2/OR8D4/OR2G3/OR2T4/OR4K5/OR4A16/OR1M1/OR1L3/OR4N2/OR52W1/OR10A3/OR2A5/OR10H2/OR10A6/OR5F1/OR2M5/OR4C11/OR9G4/OR5J2/OR8H2/OR1D2/OR4C46/OR2T27/OR2M2/OR10Z1/OR1D5/OR8H1/OR1L4/OR2T2/OR2T6/OR2S2/OR13C4/OR52A1/OR8K1/OR4A5/OR4D10/OR5AU1/OR2J2/OR2F1/OR4D9/OR52B4/OR2AK2/OR4M1/OR4L1/OR1I1/CALM1/OR8J1/OR1L1/OR4S1/OR8H3/OR4F5/OR9Q2/OR10C1/OR4M2/OR5P2/OR4K14/OR6C75/OR52J3/OR51F2/OR10G4/OR1B1/OR52A5/OR6B1/OR2J3/OR9A2/OR8G2P/OR10H4/OR13H1 |
| KEGG_CELL_ADHESION_MOLECULES_CAMS | KEGG_CELL_ADHESION_MOLECULES_CAMS | 127 | 0.418472 | 1.856642 | 1.15E-04 | 0.001176 | 0.000727 | 7372 | tags=37%, list=13%, signal=32% | CNTN1/SELE/CDH15/HLA-DRB5/VCAN/NRXN2/SELP/MPZ/HLA-F/HLA-B/HLA-DPA1/HLA-DQB1/ITGA9/ICAM1/HLA-DRB1/CD226/HLA-DRA/CD40LG/CD4/CDH5/HLA-DMA/HLA-DMB/CD28/SIGLEC1/HLA-DOA/NFASC/HLA-DPB1/CLDN7/JAM2/CD34/F11R/NCAM2/HLA-C/ESAM/HLA-A/SELL/CTLA4/CD6/CLDN5/PECAM1/ICOS/SDC4/HLA-E/HLA-DOB/SELPLG/HLA-DQA2/HLA-G |
| KEGG_CHEMOKINE_SIGNALING_PATHWAY | KEGG_CHEMOKINE_SIGNALING_PATHWAY | 185 | 0.372567 | 1.74315 | 0.000138 | 0.001339 | 0.000827 | 9472 | tags=32%, list=17%, signal=27% | CXCL2/PPBP/PF4/PF4V1/CXCL6/TIAM1/CXCL16/CCL19/XCR1/CXCL8/CXCL1/CXCR6/CCL13/CXCL3/CCL11/XCL2/CCL18/CCR8/CCR7/CXCR2/CCL24/CXCR1/STAT3/CCL21/CXCL12/ADCY4/CCL22/CCL4/CCR4/GNB5/ITK/LYN/CCR6/CCL23/CCL3/GNG12/CXCR4/CCL25/CCL3L3/CRK/PRKACA/STAT2/ADCY1/PLCB2/ARRB2/PRKCB/PIK3R5/DOCK2/CCL14/GRK5/CCL1/ADCY2/HCK/GNG11/NFKB1/GNGT2/NFKBIA/IKBKG/CCL2/CCR5 |
| KEGG_RETINOL_METABOLISM | KEGG_RETINOL_METABOLISM | 64 | 0.501802 | 1.964773 | 0.000184 | 0.001695 | 0.001047 | 4242 | tags=37%, list=8%, signal=34% | UGT2B17/UGT2A1/CYP2B6/UGT2B7/CYP2C18/ADH4/UGT2B15/DHRS9/CYP2C9/RDH16/BCO1/CYP2C8/UGT2B4/UGT2B10/ADH1A/ADH1C/ALDH1A2/CYP4A11/ADH7/CYP3A5/CYP2A6/UGT2A1/RDH10/DHRS3 |
| KEGG_DNA_REPLICATION | KEGG_DNA_REPLICATION | 36 | -0.65756 | -2.00257 | 0.000244 | 0.002138 | 0.001321 | 14456 | tags=75%, list=26%, signal=56% | PCNA/RPA2/RPA1/RFC1/RNASEH2B/POLE/POLD2/RFC5/RNASEH2C/POLA1/FEN1/POLA2/DNA2/PRIM2/RNASEH2A/POLE2/MCM4/LIG1/RFC4/RFC3/PRIM1/MCM6/MCM5/POLD1/MCM7/MCM3/MCM2 |
| KEGG_LINOLEIC_ACID_METABOLISM | KEGG_LINOLEIC_ACID_METABOLISM | 29 | 0.601055 | 2.001236 | 0.000605 | 0.004626 | 0.002858 | 6941 | tags=45%, list=12%, signal=39% | PLA2G2A/PLA2G2F/PLA2G1B/CYP2C18/CYP2C9/CYP2C8/PLA2G5/CYP3A5/PLA2G12A/PLA2G4A/AKR1B10/CYP2E1/PLA2G4B |
| KEGG_WNT_SIGNALING_PATHWAY | KEGG_WNT_SIGNALING_PATHWAY | 150 | -0.43595 | -1.66786 | 0.000614 | 0.004626 | 0.002858 | 10578 | tags=31%, list=19%, signal=25% | TCF7L1/WNT9A/DVL2/CSNK1E/CCND2/PLCB3/ROCK2/WNT8A/MMP7/VANGL1/PLCB1/CTNNBIP1/PLCB4/SMAD3/PPP2R5D/RAC3/DAAM2/NKD2/PRICKLE2/DVL3/TBL1Y/PRICKLE1/PRKCA/WNT6/PPP3R2/WNT7A/FZD7/FZD1/LEF1/TCF7/WNT5A/VANGL2/FZD2/CAMK2A/CHP2/FZD9/AXIN2/PRKCG/DKK4/WNT8B/NKD1/CER1/DKK1/WNT7B/SFRP5/WIF1 |
| KEGG_ASTHMA | KEGG_ASTHMA | 28 | 0.620331 | 2.038833 | 0.000619 | 0.004626 | 0.002858 | 7261 | tags=57%, list=13%, signal=50% | HLA-DRB5/PRG2/CCL11/HLA-DPA1/HLA-DQB1/HLA-DRB1/HLA-DRA/MS4A2/CD40LG/HLA-DMA/HLA-DMB/HLA-DOA/HLA-DPB1/IL10/HLA-DOB/HLA-DQA2 |
| KEGG_TRYPTOPHAN_METABOLISM | KEGG_TRYPTOPHAN_METABOLISM | 39 | 0.560475 | 2.018554 | 0.000629 | 0.004626 | 0.002858 | 4867 | tags=36%, list=9%, signal=33% | KMO/IDO2/KYNU/TDO2/ACMSD/AOC1/INMT/ACAT1/GCDH/AADAT/OGDHL/ALDH2/HAAO/AOX1 |
| KEGG_FATTY_ACID_METABOLISM | KEGG_FATTY_ACID_METABOLISM | 42 | 0.537321 | 1.949463 | 0.000699 | 0.004944 | 0.003055 | 11063 | tags=55%, list=20%, signal=44% | ACSL1/ADH4/ACSL4/ACAT1/CPT1A/GCDH/ADH1A/ACADL/ADH1C/CYP4A11/ADH7/ALDH2/ACADS/ACADVL/ACADSB/ACADM/HADH/CPT2/ACOX1/ALDH7A1/ALDH9A1/EHHADH/ADH1B |
| KEGG_CARDIAC_MUSCLE_CONTRACTION | KEGG_CARDIAC_MUSCLE_CONTRACTION | 78 | -0.49805 | -1.741 | 0.000914 | 0.006231 | 0.00385 | 3446 | tags=34%, list=6%, signal=32% | ATP1B3/CACNG8/COX7B2/CACNB1/FXYD2/CACNA2D3/CACNA1S/SLC9A1/ATP1A1/CACNB3/CACNA1F/ATP1A3/CACNG5/CACNG6/ATP1A2/CACNG2/CACNB4/CACNA2D2/ATP1A4/MYH7/CACNG7/TNNI3/CACNG1/COX8C/TNNT2/TNNC1/CACNG4 |
| KEGG_DILATED_CARDIOMYOPATHY | KEGG_DILATED_CARDIOMYOPATHY | 90 | -0.47555 | -1.70621 | 0.000973 | 0.006384 | 0.003945 | 8407 | tags=30%, list=15%, signal=26% | ITGB5/CACNA2D3/CACNA1S/CACNB3/ADRB1/ITGA2/TGFB2/CACNA1F/ITGA3/ITGA6/CACNG5/ITGB6/CACNG6/TGFB1/ITGB8/CACNG2/CACNB4/CACNA2D2/ITGB4/MYH7/CACNG7/TNNI3/CACNG1/TNNT2/TNNC1/ADCY8/CACNG4 |
| KEGG_ARRHYTHMOGENIC_RIGHT_VENTRICULAR_CARDIOMYOPATHY_ARVC | KEGG_ARRHYTHMOGENIC_RIGHT_VENTRICULAR_CARDIOMYOPATHY_ARVC | 74 | -0.50923 | -1.76992 | 0.001019 | 0.006384 | 0.003945 | 10578 | tags=39%, list=19%, signal=32% | TCF7L1/CACNG8/ITGA2B/CACNB1/DSC2/DSG2/ITGB5/CACNA2D3/CACNA1S/CACNB3/ITGA2/CACNA1F/ITGA3/ITGA6/CACNG5/ITGB6/LEF1/TCF7/CACNG6/ITGB8/CACNG2/CACNB4/CACNA2D2/ITGB4/CACNG7/CACNG1/ACTN3/ACTN2/CACNG4 |
| KEGG_PROPANOATE_METABOLISM | KEGG_PROPANOATE_METABOLISM | 32 | 0.59852 | 2.050932 | 0.001041 | 0.006384 | 0.003945 | 9551 | tags=56%, list=17%, signal=47% | LDHAL6B/ABAT/ACAT1/ALDH6A1/SUCLG2/SUCLG2P2/ALDH2/PCCB/LDHC/ACACB/MLYCD/PCCA/ACADM/LDHA/MCEE/HIBCH/ALDH7A1/ALDH9A1 |
| KEGG_VALINE_LEUCINE_AND_ISOLEUCINE_DEGRADATION | KEGG_VALINE_LEUCINE_AND_ISOLEUCINE_DEGRADATION | 43 | 0.552637 | 2.025643 | 0.001123 | 0.006664 | 0.004117 | 9551 | tags=56%, list=17%, signal=46% | ABAT/ACAT1/ALDH6A1/HMGCL/ALDH2/PCCB/HMGCS2/OXCT2/ACADS/AOX1/BCKDHB/PCCA/IVD/ACADSB/HSD17B10/MCCC2/ACADM/HADH/MCEE/BCKDHA/HIBCH/DBT/ALDH7A1/ALDH9A1 |
| KEGG_INTESTINAL_IMMUNE_NETWORK_FOR_IGA_PRODUCTION | KEGG_INTESTINAL_IMMUNE_NETWORK_FOR_IGA_PRODUCTION | 46 | 0.525488 | 1.944201 | 0.001218 | 0.007003 | 0.004327 | 7314 | tags=53%, list=13%, signal=46% | IL6/HLA-DRB5/IL2/HLA-DPA1/HLA-DQB1/CXCL12/HLA-DRB1/HLA-DRA/PIGR/CD40LG/HLA-DMA/HLA-DMB/CD28/HLA-DOA/HLA-DPB1/TNFSF13/TNFSF13B/CXCR4/CCL25/IL10/ICOS/HLA-DOB/TNFRSF13B/HLA-DQA2/IL15RA |
| KEGG_GAP_JUNCTION | KEGG_GAP_JUNCTION | 88 | -0.47858 | -1.70762 | 0.001339 | 0.007201 | 0.004449 | 13017 | tags=44%, list=23%, signal=34% | TUBA3D/GNAS/MAPK7/TUBB2B/TUBA1C/MAPK3/PDGFB/PRKX/PDGFRA/MAPK1/TUBB6/DRD1/PLCB3/PDGFD/PLCB1/TUBA8/PLCB4/TUBA1B/ADRB1/TUBB/TUBB3/PRKG2/GNAI1/PRKCA/HTR2B/LPAR1/SRC/TUBB7P/CDK1/TUBA3E/TUBB4A/ITPR3/GRM1/DRD2/TUBB8/PRKCG/ADCY8/GRM5/TUBA3C |
| KEGG_CALCIUM_SIGNALING_PATHWAY | KEGG_CALCIUM_SIGNALING_PATHWAY | 177 | -0.40954 | -1.58788 | 0.001349 | 0.007201 | 0.004449 | 6058 | tags=24%, list=11%, signal=21% | ADORA2A/ATP2B3/OXTR/PRKCA/CACNA1F/ITPKA/GRIN2A/HTR2B/PPP3R2/MYLK3/TRPC1/PLCG1/PTGFR/SLC8A2/P2RX7/MYLK2/CHRNA7/GRIN1/CHRM1/TACR2/CACNA1G/CACNA1B/CALML3/ADORA2B/CALML5/ITPR3/CAMK2A/CHP2/GRM1/ERBB4/PLCD3/PRKCG/CACNA1I/NOS1/TNNC1/AVPR1B/ADCY8/SPHK1/GRPR/GRM5/CCKBR/TACR3 |
| KEGG_SPLICEOSOME | KEGG_SPLICEOSOME | 126 | -0.44294 | -1.66217 | 0.00137 | 0.007201 | 0.004449 | 20675 | tags=65%, list=37%, signal=41% | PRPF40A/AQR/SF3B6/HNRNPA1P60/LSM8/SRSF2/DDX46/HSPA1A/PQBP1/DDX39B/SF3A3/CWC15/SNW1/THOC2/PPIE/U2AF1/SF3A1/SNRPG/NCBP1/PRPF6/SF3B3/SRSF9/XAB2/CRNKL1/PRPF38A/PPIH/SF3B1/TXNL4A/LSM7/SNRNP40/SNRNP70/HNRNPU/SNRPB2/CCDC12/THOC3/U2AF2/SF3B2/PHF5A/SNRPD2/DDX42/WBP11/BUD31/PPIL1/LSM4/EIF4A3/TCERG1/PRPF31/CHERP/SNRPA1/SNRPF/DHX16/HNRNPA3/PRPF19/RBMX/SNRPC/SNRPD3/RBM17/ACIN1/DHX8/SART1/THOC1/SNRNP200/HNRNPA1L2/LSM2/U2SURP/ISY1/PRPF3/NCBP2/SNRPD1/EFTUD2/USP39/HNRNPA1/CTNNBL1/SNRPA/ALYREF/PRPF40B/HSPA1L/SF3B4/HSPA2/SNRPB/SF3A2/HSPA6 |
| KEGG_PEROXISOME | KEGG_PEROXISOME | 78 | 0.433811 | 1.7623 | 0.00144 | 0.00736 | 0.004547 | 9303 | tags=31%, list=17%, signal=26% | SOD2/ACSL1/HAO2/SLC27A2/ACSL4/AGXT/HAO1/IDH2/HMGCL/XDH/NUDT12/BAAT/SOD1/MLYCD/PXMP2/MPV17L/PEX3/CAT/FAR2/DECR2/SCP2/ACOX1/PEX11G/CRAT |
| KEGG_CITRATE_CYCLE_TCA_CYCLE | KEGG_CITRATE_CYCLE_TCA_CYCLE | 31 | 0.547242 | 1.860718 | 0.002027 | 0.01008 | 0.006228 | 9152 | tags=45%, list=16%, signal=38% | PCK1/PC/ACO1/OGDHL/SUCLG2/IDH2/SUCLG2P2/FH/SDHB/SDHA/DLST/MDH2/SDHD/IDH3A |
| KEGG_O_GLYCAN_BIOSYNTHESIS | KEGG_O_GLYCAN_BIOSYNTHESIS | 29 | 0.551651 | 1.836745 | 0.002651 | 0.012837 | 0.007931 | 2972 | tags=21%, list=5%, signal=20% | GALNTL6/ST6GALNAC1/GALNT7/GALNT5/GALNT15/GALNT16 |
| KEGG_TYPE_I_DIABETES_MELLITUS | KEGG_TYPE_I_DIABETES_MELLITUS | 41 | 0.511821 | 1.853883 | 0.002899 | 0.013677 | 0.00845 | 7372 | tags=56%, list=13%, signal=49% | HLA-DRB5/IL2/CPE/HLA-F/HLA-B/HLA-DPA1/HLA-DQB1/HLA-DRB1/HLA-DRA/HLA-DMA/IL12B/HLA-DMB/CD28/HLA-DOA/HLA-DPB1/HLA-C/FAS/HLA-A/PRF1/HLA-E/HLA-DOB/HLA-DQA2/HLA-G |
| KEGG_LONG_TERM_POTENTIATION | KEGG_LONG_TERM_POTENTIATION | 70 | -0.48498 | -1.66866 | 0.003619 | 0.016646 | 0.010285 | 5402 | tags=21%, list=10%, signal=19% | PRKCA/GRIN2A/PPP3R2/GRIN1/CALML3/CALML5/ITPR3/CAMK2A/CHP2/GRM1/PRKCG/RPS6KA6/GRIA2/ADCY8/GRM5 |
| KEGG_DRUG_METABOLISM_CYTOCHROME_P450 | KEGG_DRUG_METABOLISM_CYTOCHROME_P450 | 71 | 0.413979 | 1.644567 | 0.00375 | 0.016788 | 0.010373 | 5078 | tags=35%, list=9%, signal=32% | UGT2B17/UGT2A1/CYP2B6/UGT2B7/CYP2C18/FMO2/ADH4/UGT2B15/CYP2C9/CYP2C8/UGT2B4/UGT2B10/MGST1/GSTM5/ADH1A/GSTZ1/GSTA2/ADH1C/ADH7/CYP3A5/CYP2A6/UGT2A1/GSTA1/AOX1/ALDH1A3 |
| KEGG_HYPERTROPHIC_CARDIOMYOPATHY_HCM | KEGG_HYPERTROPHIC_CARDIOMYOPATHY_HCM | 83 | -0.46161 | -1.62723 | 0.003832 | 0.016788 | 0.010373 | 8407 | tags=31%, list=15%, signal=27% | ITGB5/CACNA2D3/CACNA1S/CACNB3/ITGA2/TGFB2/CACNA1F/ITGA3/ITGA6/CACNG5/ITGB6/CACNG6/TGFB1/ITGB8/CACNG2/CACNB4/CACNA2D2/PRKAA2/ITGB4/MYH7/CACNG7/TNNI3/CACNG1/TNNT2/TNNC1/CACNG4 |
| KEGG_JAK_STAT_SIGNALING_PATHWAY | KEGG_JAK_STAT_SIGNALING_PATHWAY | 153 | 0.333944 | 1.507071 | 0.00396 | 0.016945 | 0.01047 | 9428 | tags=30%, list=17%, signal=25% | IL6/IL20/SOCS3/SOCS1/CSF3/SOCS2/IL2/CRLF2/TSLP/EPO/IL7R/OSMR/STAT3/LIFR/TPO/GHR/IFNGR1/IL7/IL13RA1/IL12B/SPRY1/IL4R/IL2RB/IL24/IL6ST/IL3RA/IL2RA/IFNK/IL10/LIF/STAT2/CISH/IL10RA/IFNAR1/SPRY3/IL15RA/IL23R/PIK3R5/IL12RB1/OSM/IRF9/IL19/IFNA6/CNTFR/LEPR/IL20RB |
| KEGG_NOD_LIKE_RECEPTOR_SIGNALING_PATHWAY | KEGG_NOD_LIKE_RECEPTOR_SIGNALING_PATHWAY | 61 | 0.427348 | 1.673037 | 0.004754 | 0.019881 | 0.012284 | 5587 | tags=20%, list=10%, signal=18% | IL6/CXCL2/CXCL8/PYDC1/CXCL1/BIRC3/CCL13/CCL11/CASP1/CARD6/NLRP3/HSP90B1 |
| KEGG_ECM_RECEPTOR_INTERACTION | KEGG_ECM_RECEPTOR_INTERACTION | 83 | -0.45612 | -1.60785 | 0.00492 | 0.020117 | 0.012429 | 4598 | tags=22%, list=8%, signal=20% | SV2C/ITGA3/LAMC1/CD36/LAMA5/ITGA6/COL4A6/ITGB6/COMP/IBSP/ITGB8/ITGB4/LAMA3/SV2A/LAMC2/LAMA1/COL11A2/COL2A1 |
| KEGG_NITROGEN_METABOLISM | KEGG_NITROGEN_METABOLISM | 23 | 0.571949 | 1.80047 | 0.00558 | 0.02232 | 0.01379 | 5664 | tags=39%, list=10%, signal=35% | GLS2/CTH/HAL/CPS1/CA12/CA5A/CA2/CA6/CA13 |
| KEGG_HEMATOPOIETIC_CELL_LINEAGE | KEGG_HEMATOPOIETIC_CELL_LINEAGE | 84 | 0.403321 | 1.664549 | 0.00635 | 0.024437 | 0.015099 | 5601 | tags=30%, list=10%, signal=27% | IL6/GYPA/HLA-DRB5/CSF3/EPO/IL7R/CR1/CD14/TPO/HLA-DRB1/IL7/CD1D/HLA-DRA/CD44/GP5/FLT3/THPO/CD4/CD55/IL4R/CD34/IL3RA/CD59/CD38/IL2RA |
| KEGG_STARCH_AND_SUCROSE_METABOLISM | KEGG_STARCH_AND_SUCROSE_METABOLISM | 52 | 0.43734 | 1.674889 | 0.006375 | 0.024437 | 0.015099 | 4029 | tags=26%, list=7%, signal=25% | SI/UGT2B17/UGT2A1/UGT2B7/UGT2B15/GBA3/UGT2B4/GYS2/UGT2B10/MGAM/G6PC/PYGM/G6PC2/UGT2A1 |
| KEGG_OOCYTE_MEIOSIS | KEGG_OOCYTE_MEIOSIS | 111 | -0.41799 | -1.54711 | 0.006754 | 0.024872 | 0.015367 | 15387 | tags=43%, list=27%, signal=31% | RPS6KA3/MAD2L1/BTRC/YWHAQ/IGF1R/RBX1/RPS6KA1/CDC23/ANAPC11/CAMK2B/PPP1CC/CAMK2G/MAPK3/PRKX/PPP2R1A/ANAPC4/ANAPC1/MAPK1/SMC3/ANAPC7/FBXO5/CDK2/PPP2R5D/SPDYA/MAPK12/CDC25C/ESPL1/CCNB1/INS/BUB1/PKMYT1/PPP3R2/CCNE1/CPEB1/CDK1/PTTG1/CCNB2/FBXO43/PLK1/CDC20/SMC1B/CALML3/CALML5/ITPR3/CAMK2A/CHP2/RPS6KA6/ADCY8 |
| KEGG_CYSTEINE_AND_METHIONINE_METABOLISM | KEGG_CYSTEINE_AND_METHIONINE_METABOLISM | 34 | 0.519834 | 1.798123 | 0.006759 | 0.024872 | 0.015367 | 6686 | tags=34%, list=12%, signal=30% | SDS/CTH/TAT/CBS/GOT1/LDHAL6B/MAT1A/GOT2/LDHC/LDHA/CDO1/CBS |
| KEGG_ALANINE_ASPARTATE_AND_GLUTAMATE_METABOLISM | KEGG_ALANINE_ASPARTATE_AND_GLUTAMATE_METABOLISM | 30 | 0.523531 | 1.759694 | 0.00776 | 0.027461 | 0.016967 | 2639 | tags=37%, list=5%, signal=35% | GLS2/ASS1/AGXT2/GPT/GOT1/CPS1/ASL/GPT2/ABAT/AGXT/ALDH4A1 |
| KEGG_PORPHYRIN_AND_CHLOROPHYLL_METABOLISM | KEGG_PORPHYRIN_AND_CHLOROPHYLL_METABOLISM | 40 | 0.470812 | 1.705425 | 0.007864 | 0.027461 | 0.016967 | 5519 | tags=29%, list=10%, signal=26% | UGT2B17/UGT2A1/UGT2B7/UGT2B15/CP/UGT2B4/UGT2B10/UGT2A1/HMOX1/HMOX2/BLVRB/ALAS1 |
| KEGG_DRUG_METABOLISM_OTHER_ENZYMES | KEGG_DRUG_METABOLISM_OTHER_ENZYMES | 51 | 0.44841 | 1.700717 | 0.00791 | 0.027461 | 0.016967 | 4897 | tags=31%, list=9%, signal=28% | UGT2B17/UGT2A1/UGT2B7/NAT2/TYMP/UGT2B15/UGT2B4/UGT2B10/DPYD/XDH/CYP3A5/CYP2A6/UGT2A1/DPYS/CDA/NAT1 |
| KEGG_LEISHMANIA_INFECTION | KEGG_LEISHMANIA_INFECTION | 70 | 0.396965 | 1.580319 | 0.009188 | 0.031306 | 0.019342 | 9612 | tags=36%, list=17%, signal=30% | FOS/HLA-DRB5/PTGS2/C3/HLA-DPA1/CR1/HLA-DQB1/IFNGR1/HLA-DRB1/HLA-DRA/HLA-DMA/IL12B/HLA-DMB/HLA-DOA/HLA-DPB1/TGFB3/IL10/HLA-DOB/PRKCB/HLA-DQA2/ITGA4/NFKB1/NFKBIA/IL1A/TLR2 |
| KEGG_FOCAL_ADHESION | KEGG_FOCAL_ADHESION | 197 | -0.36538 | -1.43889 | 0.00992 | 0.033188 | 0.020505 | 4882 | tags=15%, list=9%, signal=14% | FLNA/ITGA3/PIK3R2/MYLK3/SRC/LAMC1/LAMA5/ITGA6/COL4A6/ITGB6/MYLK2/COMP/IBSP/VAV3/ITGB8/ITGB4/MYL7/PIK3R2/CAV3/PAK6/LAMA3/PRKCG/LAMC2/ACTN3/ACTN2/LAMA1/COL11A2/SHC4/RASGRF1/COL2A1 |
| KEGG_TOLL_LIKE_RECEPTOR_SIGNALING_PATHWAY | KEGG_TOLL_LIKE_RECEPTOR_SIGNALING_PATHWAY | 100 | 0.335047 | 1.428756 | 0.010638 | 0.034953 | 0.021596 | 3724 | tags=11%, list=7%, signal=10% | IL6/LBP/FOS/LY96/CXCL8/CD14/TLR3/CCL4/IL12B/IRF7/SPP1 |
| KEGG_GLYCOSPHINGOLIPID_BIOSYNTHESIS_LACTO_AND_NEOLACTO_SERIES | KEGG_GLYCOSPHINGOLIPID_BIOSYNTHESIS_LACTO_AND_NEOLACTO_SERIES | 26 | -0.5947 | -1.70344 | 0.012625 | 0.040753 | 0.025179 | 9463 | tags=50%, list=17%, signal=42% | B3GNT3/ST8SIA1/B3GNT4/FUT4/FUT2/FUT1/B4GAT1/B3GALT2/FUT7/FUT3/FUT9/B3GALT5/B3GALT1 |
| KEGG_METABOLISM_OF_XENOBIOTICS_BY_CYTOCHROME_P450 | KEGG_METABOLISM_OF_XENOBIOTICS_BY_CYTOCHROME_P450 | 69 | 0.397362 | 1.578063 | 0.014744 | 0.046775 | 0.0289 | 5078 | tags=33%, list=9%, signal=30% | UGT2B17/UGT2A1/CYP2B6/UGT2B7/CYP2C18/ADH4/UGT2B15/CYP2C9/CYP2C8/UGT2B4/UGT2B10/MGST1/GSTM5/AKR1C4/ADH1A/GSTZ1/GSTA2/ADH1C/ADH7/CYP3A5/UGT2A1/GSTA1/ALDH1A3 |
| KEGG_TYPE_II_DIABETES_MELLITUS | KEGG_TYPE_II_DIABETES_MELLITUS | 47 | -0.49697 | -1.59438 | 0.015799 | 0.049272 | 0.030442 | 5033 | tags=29%, list=9%, signal=27% | INS/PIK3R2/PRKCD/PKLR/HK1/ADIPOQ/KCNJ11/CACNA1G/CACNA1B/PDX1/PIK3R2/PKM/ABCC8/GCK |

**Supplementary Figures**


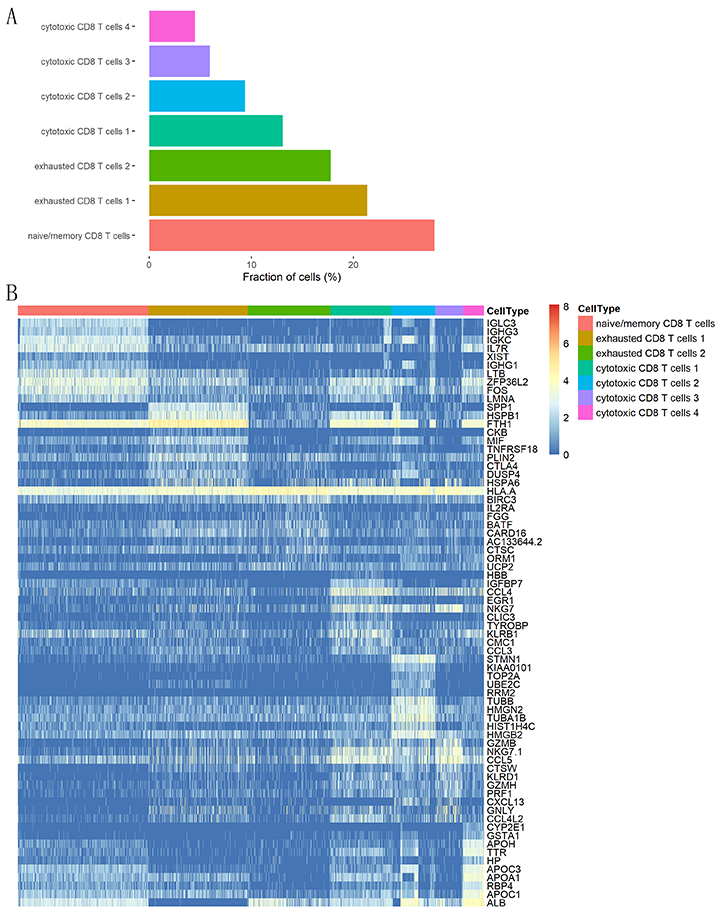


**Figure S1.** The proportion and marker expression of CD8+T subtypes. **(A)** Proportion bar chart of 7 CD8+T subtypes. **(B)** Heat maps of 7 types of CD8+T subtypes marker.


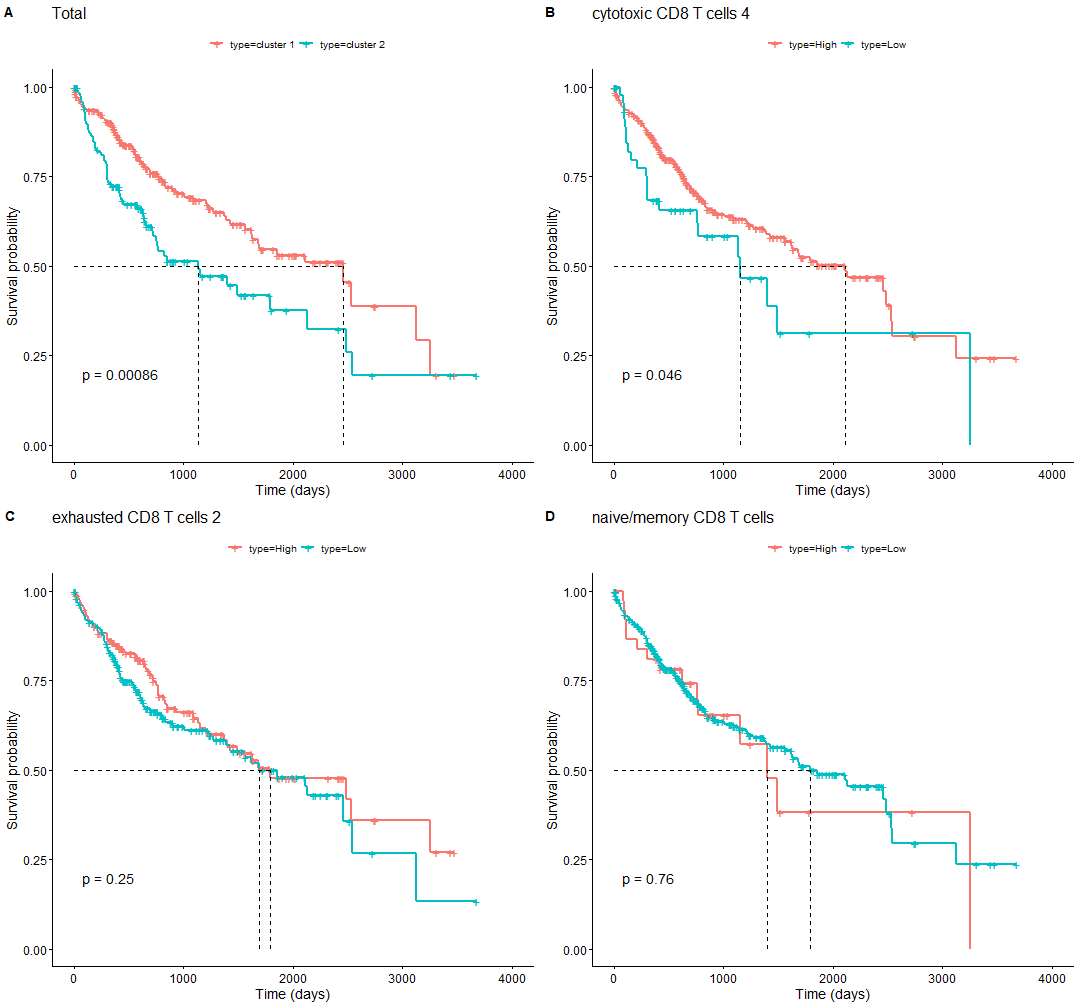


**Figure S2.** Cell subpopulation survival analysis. **(A)** The KM curve of the whole immune cell proportion grouping. **(B)** The KM curves of cytotoxic CD8 T cells 4. **(C)** The KM curves of exhausted CD8 T cells 2. **(D)** The KM curves of naive/memory CD8 T cells.


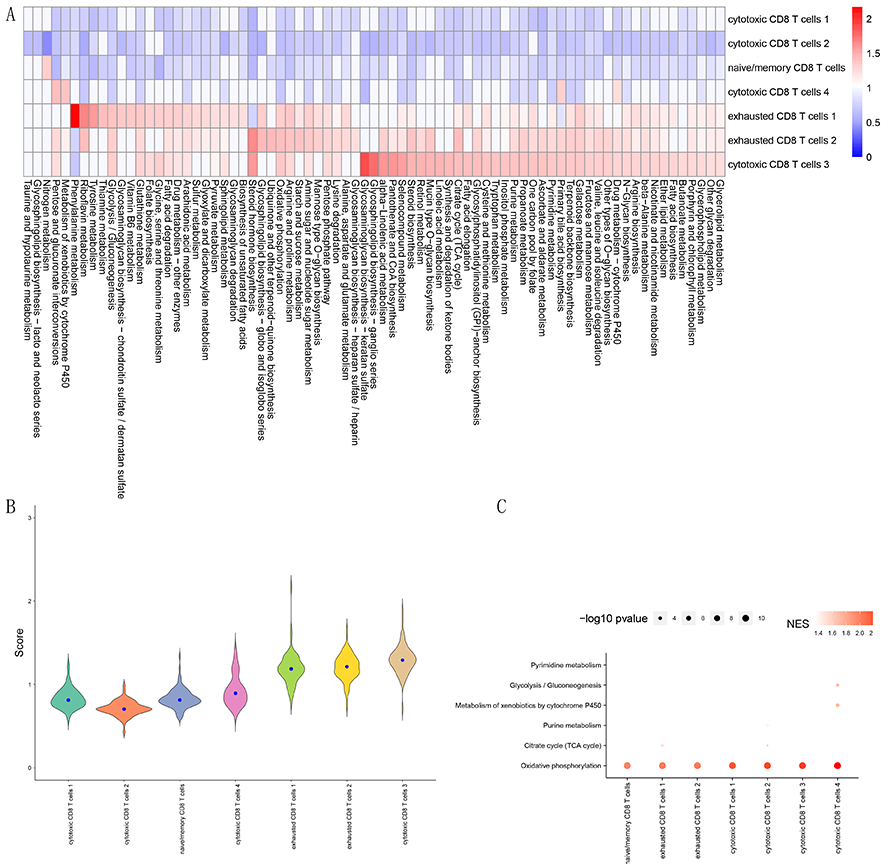


**Figure S3.** Metabolic pathway activity analysis of CD8+T subsets. **(A)** Heat map of metabolic pathway activity of CD8+T subsets. **(B)** Violin diagram of metabolic pathway activity of CD8+T subsets. **(C)** GSEA enrichment fractional point diagram of CD8+T subgroup.

**
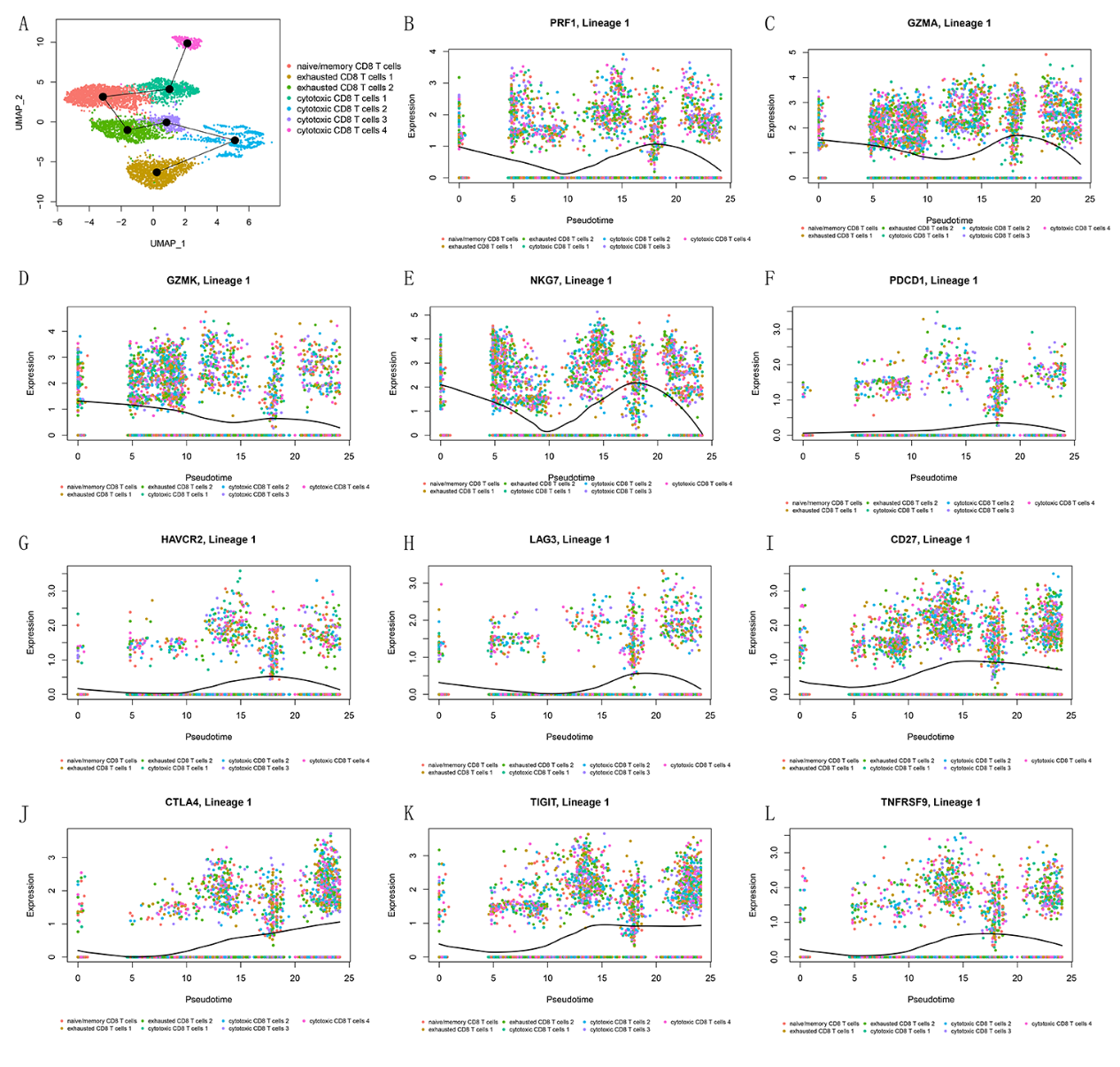
**

**Figure S4.** Cell differentiation trajectory analysis and marker gene distribution. (A) Cell differentiation trajectories of seven CD8+T subtypes. (B)-(L) Expression profile of marker genes in Lineage 1.


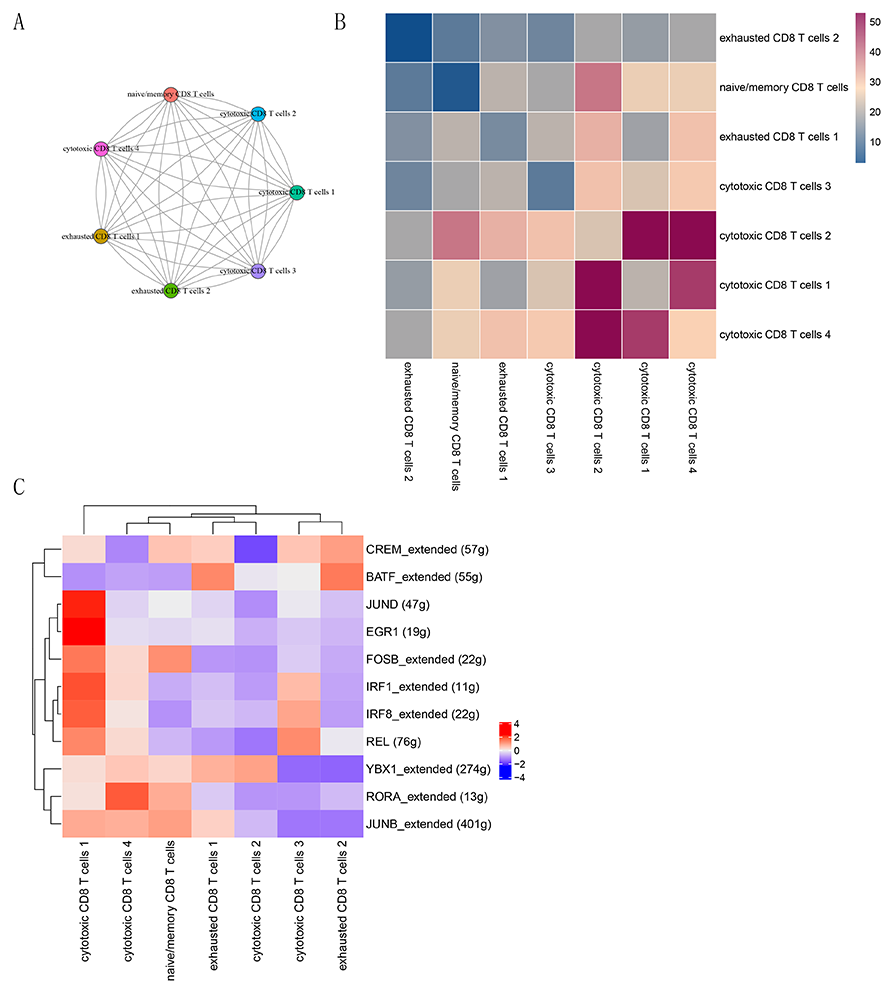


**Figure S5.** Cell-cell interaction network analysis. **(A)** Cell interaction network diagram. **(B)** Heat map of ligand-receptor pairs between seven subgroups. **(C)** Regulators of seven subgroups.


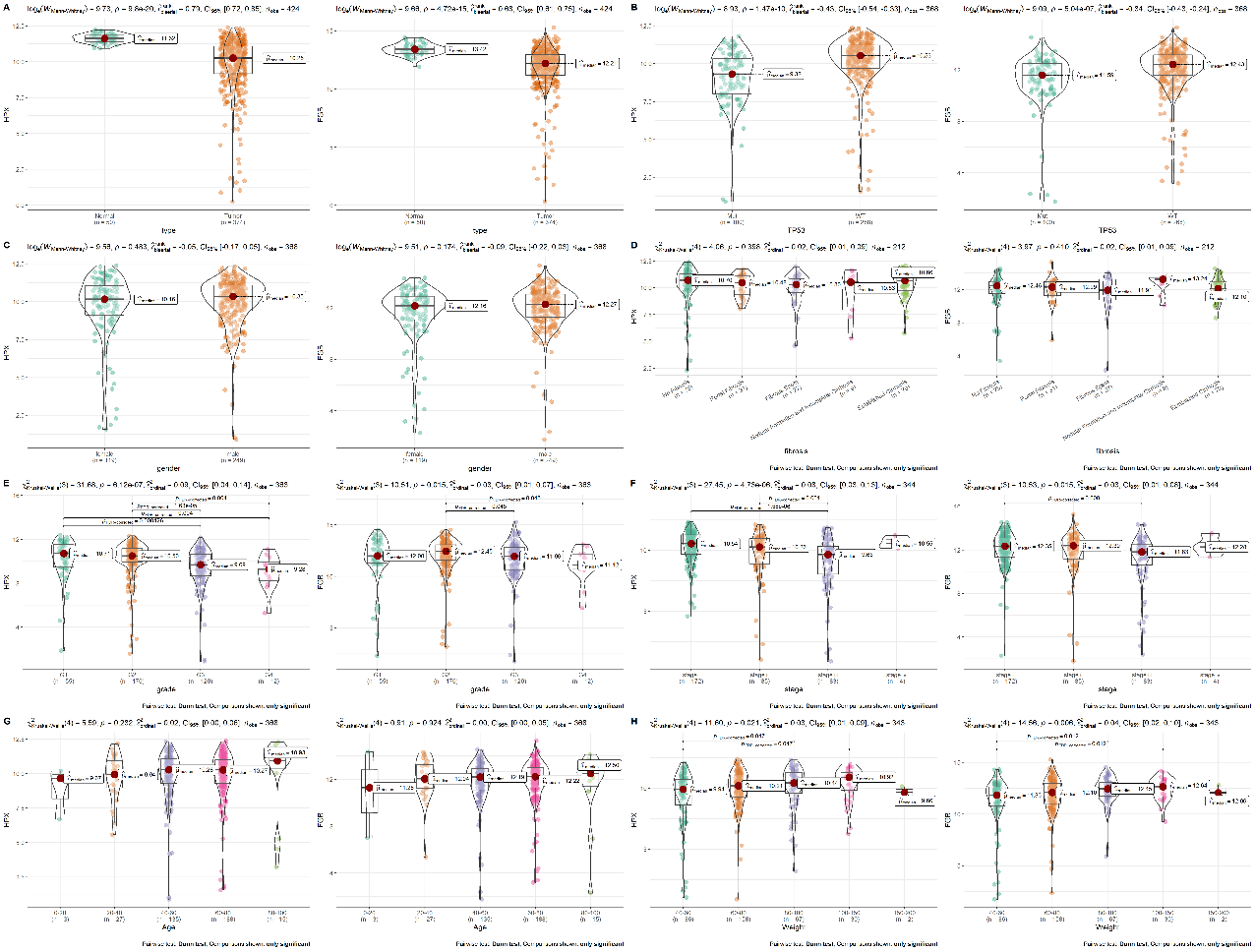


**Figure S6.** Expression of genes HPX and FGB under different clinical characteristics. **(A)-(H)** are violin diagrams showing the expression differences of genes HPX (left) and FGB (right) in normal and tumor tissues, TP53 mutant and non-mutant patients, gender, degree of liver cirrhosis, cancer grade, cancer stage, different ages and different body weights; The degree of liver cirrhosis was divided into no fibrosis, portal fibrosis, nodural formation and incomplete cirrhosis and established cirrhosis; Age: 0-20, 20-40, 40-60, 60-80 and 80-100; Body weight is divided into 40-60, 60-80, 80-100, 100-150 and 150-200.


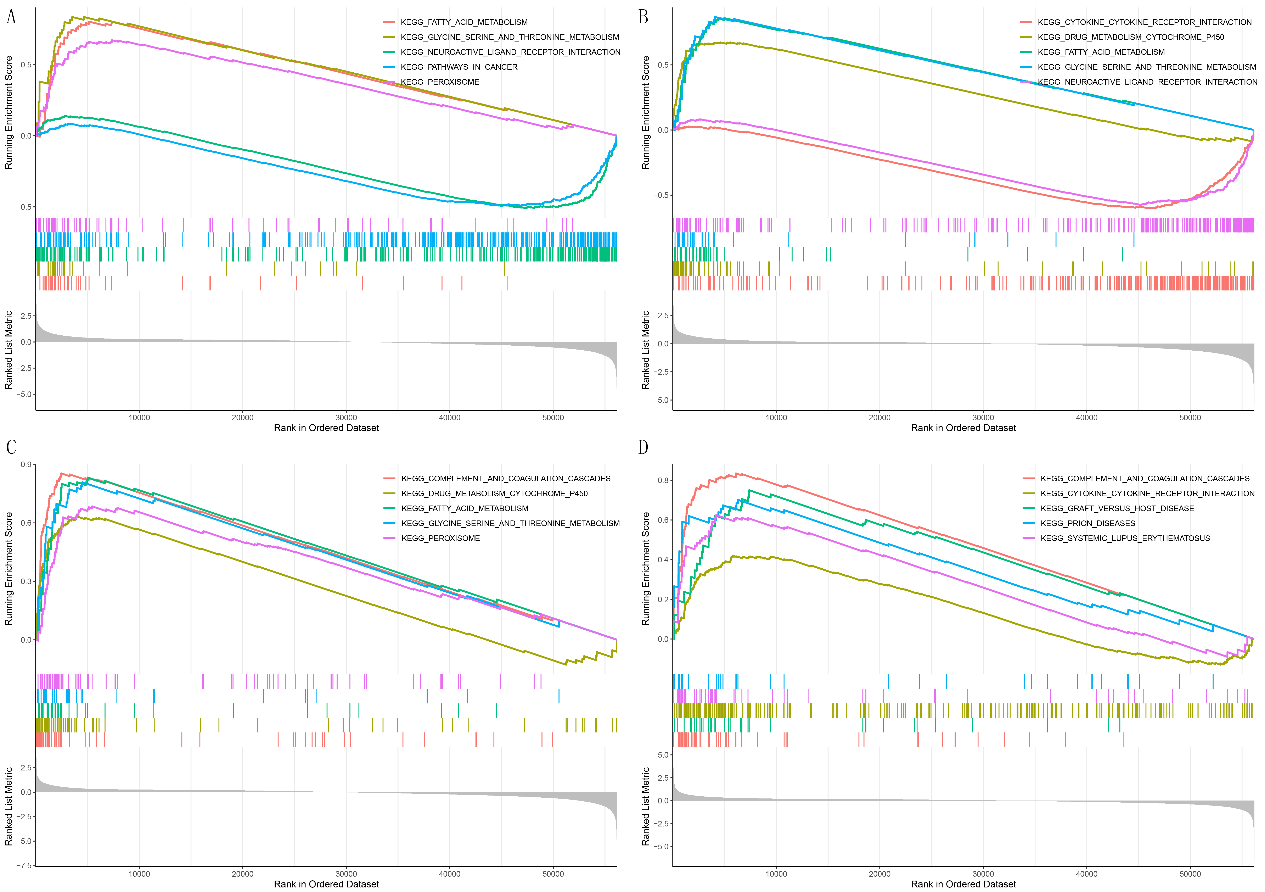


**Figure S7.** Single-gene GSEA analysis of HUB genes. **(A)** APOC3 single-gene GSEA analysis, showing the first 5 enrichment pathways. **(B)** ApoH single gene GSEA analysis, display diagram of the first 5 enrichment pathways. **(C)** HPX single-gene GSEA analysis, display diagram of the first 5 enrichment pathways. **(D)** FGB single gene GSEA analysis, display diagram of the first 5 enrichment pathways.

**
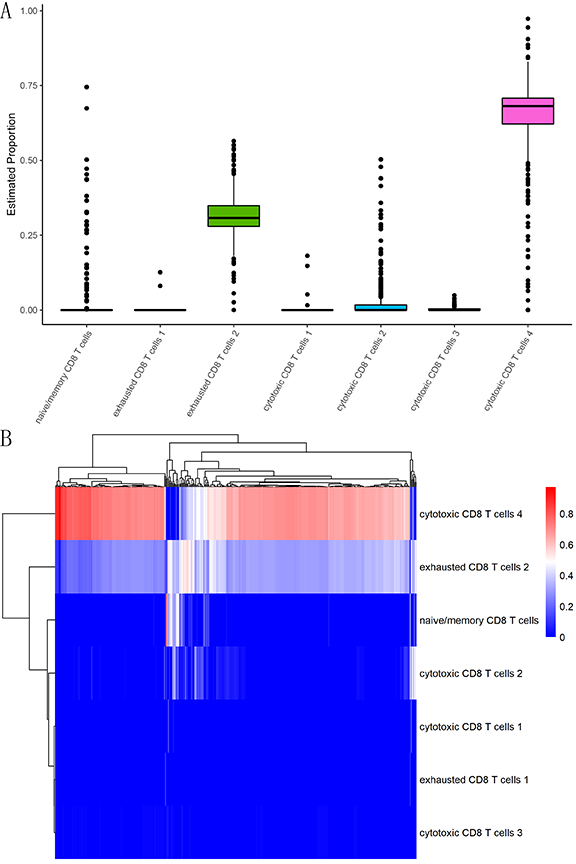
**

**Figure S8.** Proportion of TCGA-LIHC immune cells. **(A)** Box plot of the proportion of cells of the seven CD8+T subtypes. **(B)** Heat map of the proportion of the seven CD8+T subtypes.

**
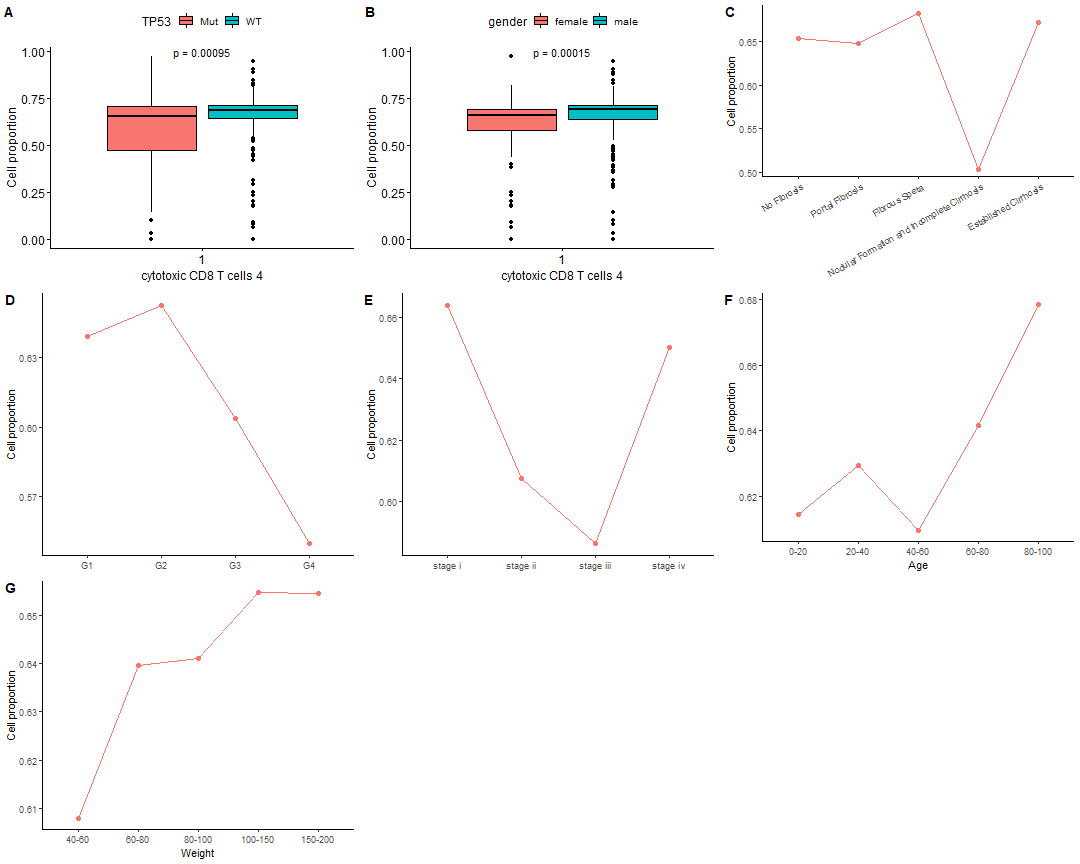
**

**Figure S9.** Proportion of cytotoxic CD8 T cells 4 in different clinical features. **(A), (B)** Box plots show the ratio of CD8 T cells 4 with or without TP53 mutation and sex, respectively. **(C)-(G)**: Line plots show the proportion of cytotoxic CD8 T cells 4 in cirrhosis degree, grade, stage, age, and body weight.


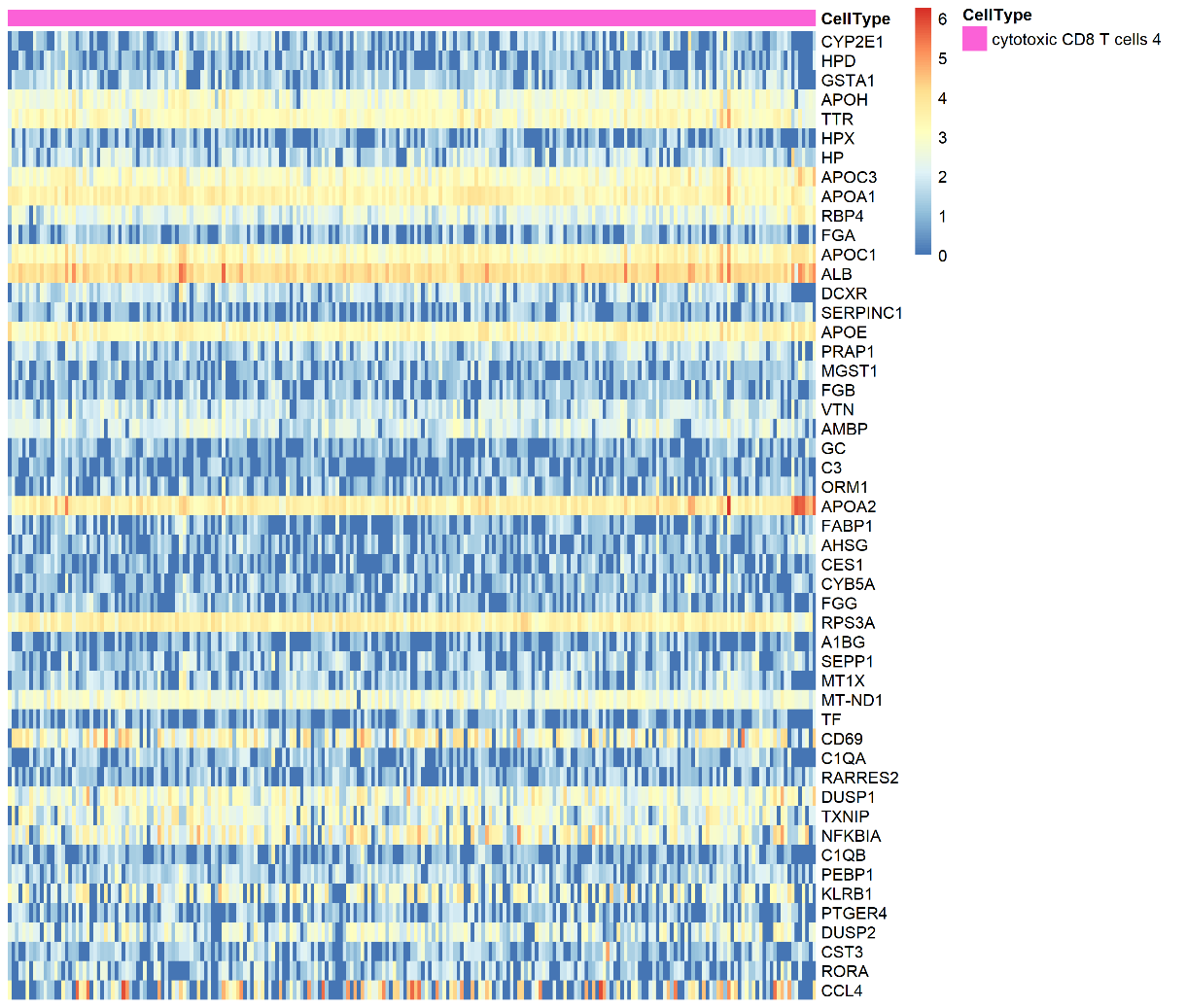


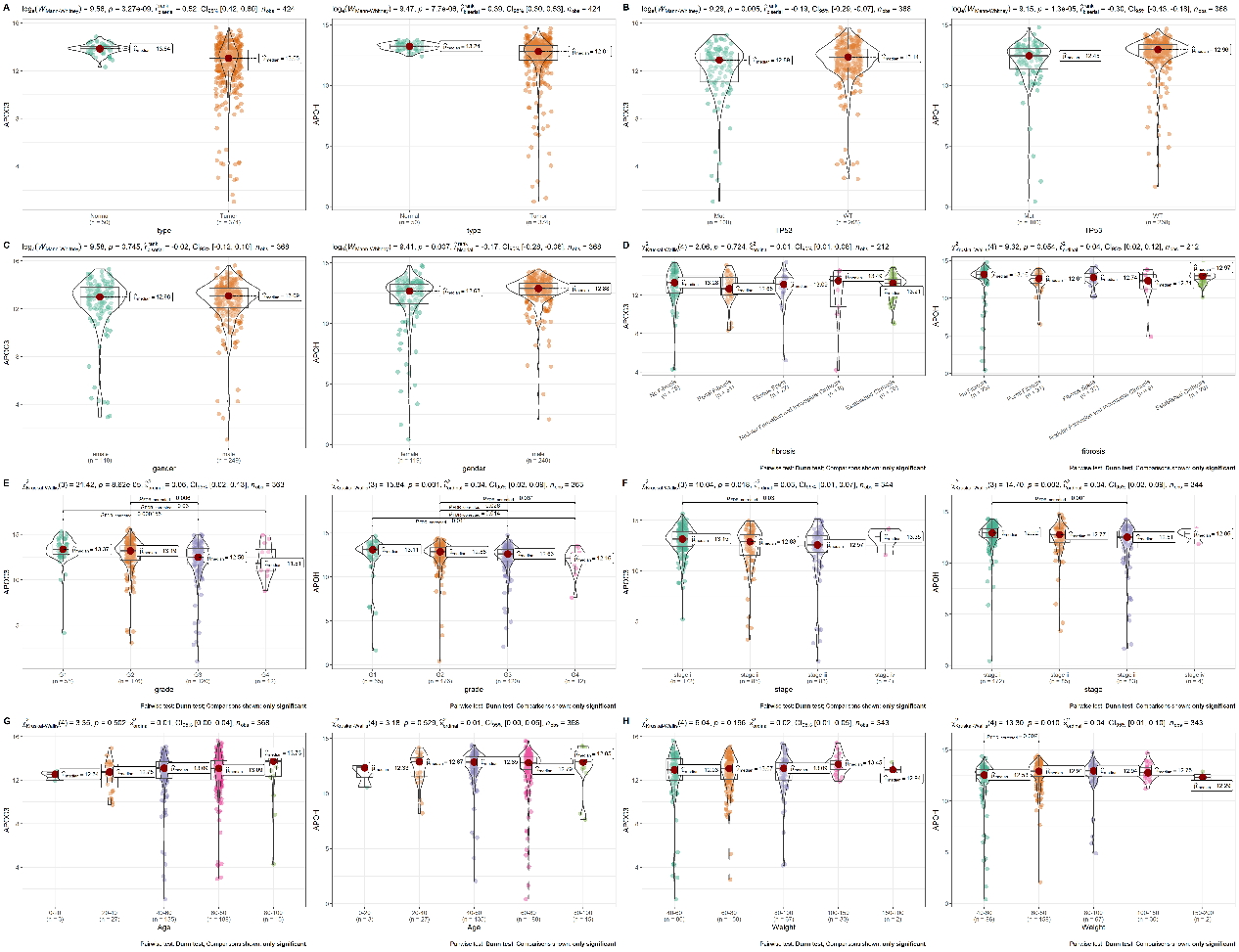
**Figure S10.** Differential analysis of CD8+T subsets

**Figure S11.** Expression of genes APOC3 and APOH in different clinical characteristics. **(A)-(H)** are violin diagrams showing the expression differences of APOC3 (left) and APOH (right) in normal and tumor tissues, TP53 mutant and non-mutant patients, gender, degree of liver cirrhosis, cancer grade, cancer stage, different ages and different body weights; The degree of liver cirrhosis was divided into no fibrosis, portal fibrosis, nodural formation and incomplete cirrhosis and established cirrhosis; Age: 0-20, 20-40, 40-60, 60-80 and 80-100; Body weight is divided into 40-60, 60-80, 80-100, 100-150 and 150-200


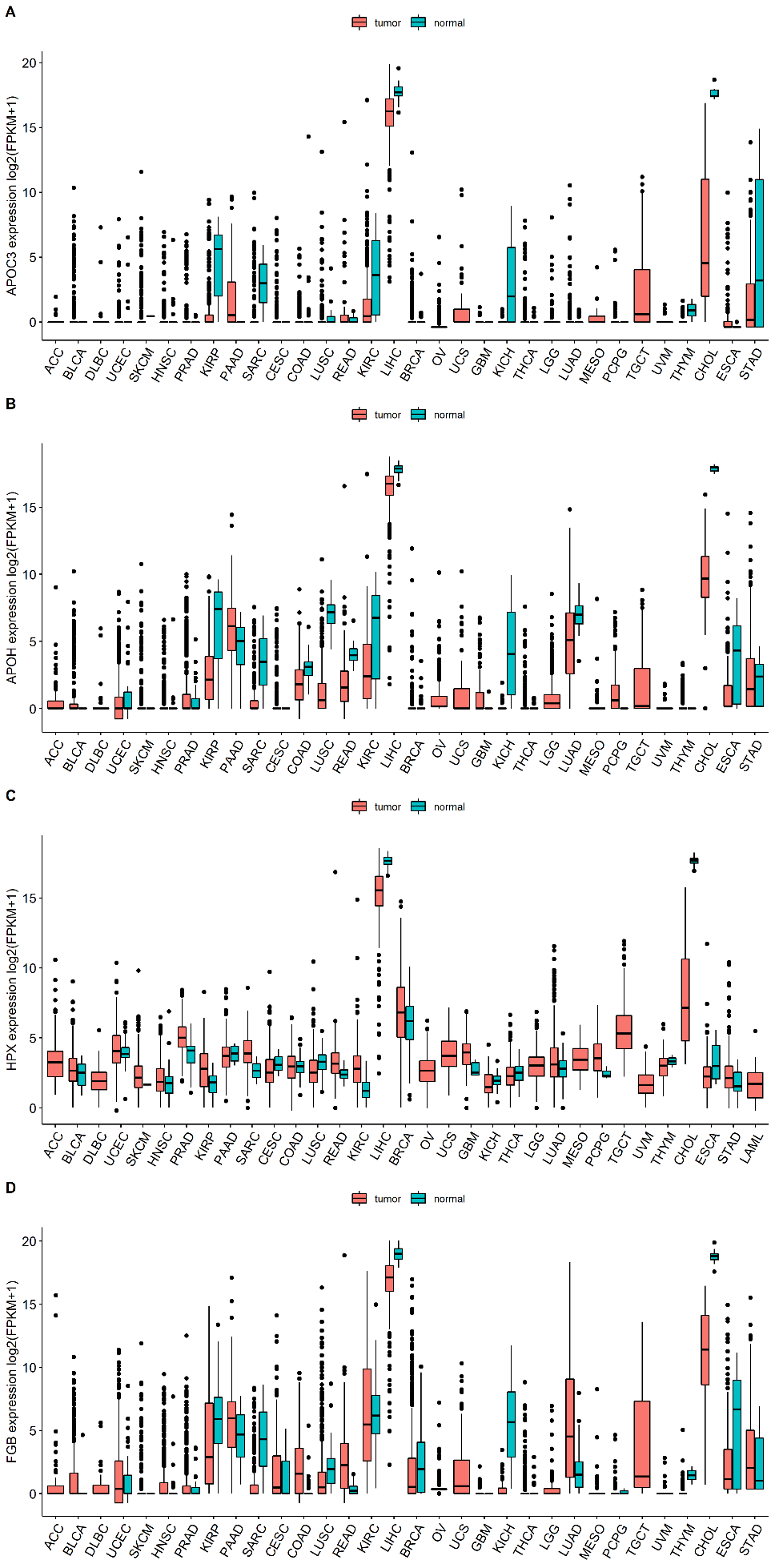


**Figure S12.** Hub gene expression in different cancer types. **(A)** Expression of APOC3 in different cancer types. **(B)** Expression of ApoH in different types of cancer. **(C)** Expression of HPX in different types of cancer. **(D)** Expression of FGB in different cancer types.
